# Supplementary material for: Quantification of heterogeneity in human CD8+ T cell responses to vaccine antigens: an HLA-guided perspective
Source: Front Immunol. 2024 Nov 18;15:1420284. doi: 10.3389/fimmu.2024.1420284 (PMC11608996; doi:10.3389/fimmu.2024.1420284)
Supplement: Supplementary file 1 [file DataSheet1.pdf]

Supplementary Material

1 COVERAGE METRICS $\mathcal{C}_k$ AND $\mathcal{I}_k$, AND THE ROLE OF HLA ALLELE ASSOCIATIONS

1.1 $\mathcal{C}_k$ and $\mathcal{I}_k$ are equal in the absence of HLA allele associations

We now show that in the absence of HLA allele associations, one has $\mathcal{C}_k = \mathcal{I}_k$. We present the proof for the case of a population with three alleles (of a given type). The arguments of the proof can be generalized to any number of alleles. Without lack of generality and to simplify the notation, we drop the regional index and the normalization symbol for the allele frequencies. We denote the three alleles by a_1, a_2, a_3, and their individual frequencies by f_1, f_2, f_3, respectively. Thus, the mean regional coverage metric (see Eq. (5) in the main text) is given by

$$\mathcal{C} \quad = \quad \frac{f_1\sigma_1 + f_2\sigma_2 + f_3\sigma_3}{f_1 + f_2 + f_3} \; . \tag{S1}$$

In a population with three alleles, $Q = \frac{3\times(3+1)}{2} = 6$, so that there are six different allele pairs given by: $(1,1), (1,2), (1,3), (2,2), (2,3), (3,3)$. Let us denote by $\rho_{q=(i,i')}$ the frequency of allele pair $q = (a_i, a_{i'})$, with $i \leq i'$ and $1 \leq i, i' \leq 3$. In the absence of HLA allele associations these frequencies are given by

$$\rho_{(1,1)} = f_1^2 \; , \quad \rho_{(1,2)} = 2f_1f_2 \; , \quad \rho_{(1,3)} = 2f_1f_3 \; , \quad \rho_{(2,2)} = f_2^2 \; , \quad \rho_{(2,3)} = 2f_2f_3 \; , \quad \rho_{(3,3)} = f_3^2 \; . \tag{S2}$$

We now make use of Eq. (7) in the main text to write

$$\mathcal{I}_{(1,1)} = \sigma_1 \; , \quad \mathcal{I}_{(1,2)} = \frac{\sigma_1+\sigma_2}{2} \; , \quad \mathcal{I}_{(1,3)} = \frac{\sigma_1+\sigma_3}{2} \; , \quad \mathcal{I}_{(2,2)} = \sigma_2 \; , \quad \mathcal{I}_{(2,3)} = \frac{\sigma_2+\sigma_3}{2} \; , \quad \mathcal{I}_{(3,3)} = \sigma_3 \; . \tag{S3}$$

We note that the denominator of Eq. (8) in the main text is equal to $(f_1 + f_2 + f_3)^2$, so that we can write

$$\mathcal{I} \quad = \quad \frac{f_1^2\sigma_1 + f_1f_2(\sigma_1+\sigma_2) + f_1f_3(\sigma_1+\sigma_3) + f_2^2\sigma_2 + f_2f_3(\sigma_2+\sigma_3) + f_3^2\sigma_3}{(f_1+f_2+f_3)^2} \; .$$

We now collect the factors of $\sigma_1, \sigma_2, \sigma_3$ as follows

$$\mathcal{I} = \frac{f_1\sigma_1(f_1+f_2+f_3) + f_2\sigma_2(f_1+f_2+f_3) + f_3\sigma_3(f_1+f_2+f_3)}{(f_1+f_2+f_3)^2} = \frac{f_1\sigma_1 + f_2\sigma_2 + f_3\sigma_3}{(f_1+f_2+f_3)} = \mathcal{C} \; ,$$

as we wanted to show. The arguments of the proof can also be generalized to M alleles, making use of an induction argument.

1.2 $\mathcal{C}_k$ and $\mathcal{I}_k$ are equal in the presence of HLA allele associations

In the presence of HLA allele associations, and for the case of two alleles, the frequencies are given by [1]

$$\rho_{(1,1)} = f_1^2 + \epsilon \; , \quad \rho_{(1,2)} = 2(f_1f_2 - \epsilon) \; , \quad \rho_{(2,2)} = f_2^2 + \epsilon \; , \tag{S4}$$

[1] One can show that for the case of two discrete and identically distributed random variables (with two possible states each, a_1 and a_2), the joint probability distribution can be written as in Eq. (S4), so that the correlations are encoded in the parameter ϵ.

with ϵ characterizing allele correlations. We note that $\epsilon \in \mathbb{R}$, so that it can be positive or negative. If $\epsilon = 0$, allele correlations vanish. The values of $\mathcal{I}_{q=(i,i')}$ for pair $q = (a_i, a_{i'})$ are those shown above (see Eq. (S3) in Section 1.1 of the Supplementary Material). Therefore, we have

$$\mathcal{I}_{(1,1)} = \sigma_1 \ , \quad \mathcal{I}_{(1,2)} = \frac{\sigma_1 + \sigma_2}{2} \ , \quad \mathcal{I}_{(2,2)} = \sigma_2 \ . \tag{S5}$$

Thus, we can write

$$\mathcal{I} \;\; = \;\; \frac{(f_1^2 + \epsilon)\sigma_1 + (f_1 f_2 - \epsilon)(\sigma_1 + \sigma_2) + (f_2^2 + \epsilon)\sigma_2}{(f_1 + f_2)^2} \ .$$

We now collect the factors with and without ϵ as follows

$$\mathcal{I} \;\; = \;\; \frac{(f_1\sigma_1 + f_2\sigma_2)(f_1 + f_2) + \epsilon(\sigma_1 - \sigma_1 - \sigma_2 + \sigma_2)}{(f_1 + f_2)^2} = \frac{f_1\sigma_1 + f_2\sigma_2}{(f_1 + f_2)} = \mathcal{C} \ ,$$

as we wanted to show. The arguments of the proof can also be generalized to M alleles, making use of an induction argument. We note that the reason HLA allele associations (terms proportional to ϵ) do not appear in $\mathcal{I}$ is the fact that Eq. (7) in the main text is linear in its entries; that is, our choice of the individual regional coverage metric, $\mathcal{I}_q^{(k)}$, for allele pair $q = (a_i, a_{i'})$ (for a given region) is the arithmetic mean of σ_i and σ_i'. This choice for the variable $\mathcal{I}_q^{(k)}$ implies HLA allele correlations play no role in the mean individual regional coverage metric $\mathcal{I}_k$ (Eq. (8) in the main text).

We now ask ourselves, in what follows, if this is a reasonable choice for $\mathcal{I}_q^{(k)}$. To that end we explore other (non-linear) choices for this variable.

1.3 First choice for $\mathcal{I}_q^{(k)}$: dominance of one allele

Let us imagine that in the presence of two alleles in a given individual, there exists a dominance of one of them to bind the peptide (nonamer) of choice, and thus, one can suggest the following choice for $\mathcal{I}_q$ [2]:

$$\mathcal{I'}_q \;\; = \;\; \max\{\sigma_i, \sigma_i'\} \ , \tag{S6}$$

where $q = (a_i, a_{i'})$. We make use of the allele frequencies described in Section 1.2 of the Supplementary Material (see Eq. (S4)), and of the values for $\mathcal{I}_q$ defined by Eq. (S6),

$$\mathcal{I'}_{(1,1)} = \sigma_1 \ , \quad \mathcal{I'}_{(1,2)} = \max\{\sigma_1, \sigma_2\} \ , \quad \mathcal{I'}_{(2,2)} = \sigma_2 \ , \tag{S7}$$

to compute $\mathcal{I}'$ as follows

$$\begin{aligned} \mathcal{I}' \;\; &= \;\; \frac{(f_1^2 + \epsilon)\sigma_1 + 2(f_1 f_2 - \epsilon)\max\{\sigma_1, \sigma_2\} + (f_2^2 + \epsilon)\sigma_2}{(f_1 + f_2)^2} \\ &= \;\; \frac{f_1^2\sigma_1 + 2f_1 f_2 \max\{\sigma_1, \sigma_2\} + f_2^2\sigma_2 + \epsilon(\sigma_1 + \sigma_2 - 2\max\{\sigma_1, \sigma_2\})}{(f_1 + f_2)^2} \ . \end{aligned}$$

The previous equation clearly shows that $\mathcal{I}' \neq \mathcal{C}$, even in the absence of correlations (if $\epsilon = 0$), and that the mean individual regional coverage metric, $\mathcal{I}'$, depends on ϵ. The term in $\mathcal{I}'$ proportional to ϵ,

[2] We remind the reader that we drop the regional upper index for ease of notation in what follows, and we will write $\mathcal{I}_q$ instead of $\mathcal{I}_q^{(k)}$.

$(\sigma_1 + \sigma_2 - 2\max\{\sigma_1, \sigma_2\})$, is always negative. This means $\mathcal{I}'_{\epsilon>0} < \mathcal{I}'_{\epsilon=0} < \mathcal{I}'_{\epsilon<0}$, which in turn implies that if there is a positive correlation for homozygous alleles, so that $\epsilon > 0$, and therefore a negative correlation for heterozygous alleles, the mean individual regional coverage metric, $\mathcal{I}'$, will be lower than in the case when $\epsilon < 0$, and there is a negative correlation for homozygous alleles (and therefore a positive correlation for heterozygous alleles). Our results indicate that if the individual regional coverage metric, $mathcalI'_q$, is defined by Eq. (S6), then the presence of a positive (negative) correlation for homozygous alleles leads to a lower (greater) value for the mean individual regional coverage metric, $\mathcal{I}'$, when compared to $\mathcal{I}'_{\epsilon=0}$; that is, neglecting the presence of a positive (negative) correlation for homozygous alleles leads to over (under) estimating the value of the mean individual regional coverage metric.

1.4 Second choice for $\mathcal{I}_q^{(k)}$: chemical reaction analysis

We now provide a second choice for $\mathcal{I}_q$ based on a chemical reaction analysis. For the purposes of this analysis, let us assume that there is only one peptide, p, that can be presented by allele a. The binding score for the pair (a, p) correlates with the affinity constant, $\mathcal{K}_A$, of the binding. The affinity (equilibrium) constant is determined by the ratio of the *on* and *off* rates of the following chemical reactions between the allele and the peptide

$$a + p \underset{k_{off}}{\overset{k_{on}}{\rightleftharpoons}} a : p \; ; \tag{S8}$$

that is $\mathcal{K}_A = \frac{k_{\text{on}}}{k_{\text{off}}}$. It is more convenient to make use of the dissociation (equilibrium) constant (with dimensions of concentration), $\mathcal{K}_D = \frac{k_{\text{off}}}{k_{\text{on}}} = \frac{1}{\mathcal{K}_A}$, which is the inverse of the association (equilibrium) constant. We note that a greater binding score, and thus, a greater affinity constant, imply a lower dissociation constant. One can show that $\mathcal{K}_D$ corresponds to the value of the peptide concentration for which half of the allele molecules are bound. We now want to determine the value of $\mathcal{K}_D$ for the system of two alleles, a_1 and a_2, which compete to bind p. The following chemical reactions between the alleles and the peptide describe the system

$$a_1 + p \underset{k_{off,1}}{\overset{k_{on,1}}{\rightleftharpoons}} a_1 : p \; ; \tag{S9}$$

$$a_2 + p \underset{k_{off,2}}{\overset{k_{on,2}}{\rightleftharpoons}} a_2 : p \; ; \tag{S10}$$

with $\mathcal{K}_{D,1} = \frac{k_{\text{off},1}}{k_{\text{on},1}}$ and $\mathcal{K}_{D,2} = \frac{k_{\text{off},2}}{k_{\text{on},2}}$. Let us assume the number of peptides, N_p, is much larger than the number of alleles, $\frac{N_a}{2}$, for each a_1 and a_2, so that the number of peptides is not limiting, and the total number of alleles is N_a. In equilibrium the number of complexes of type $a_1 : p$ and $a_2 : p$ is given then by $\frac{N_a}{2}\frac{N_p}{\mathcal{K}_{D,1}+N_p}$ and $\frac{N_a}{2}\frac{N_p}{\mathcal{K}_{D,2}+N_p}$, respectively. The total number of bound alleles, that is the total number of $a_1 : p$ and $a_2 : p$ complexes is given by $\frac{N_a}{2}\frac{N_p}{\mathcal{K}_{D,1}+N_p} + \frac{N_a}{2}\frac{N_p}{\mathcal{K}_{D,2}+N_p}$. In order to obtain the $\mathcal{K}_D$ for the system of two alleles and one peptide, we need to identify the value of the peptide concentration, N_p, for which half of the allele molecules are bound. We must then solve the following equation

$$\frac{N_a}{2} = \frac{N_a}{2}\frac{N_p}{\mathcal{K}_{D,1} + N_p} + \frac{N_a}{2}\frac{N_p}{\mathcal{K}_{D,2} + N_p} \; , \tag{S11}$$

for N_p, which is equivalent to

$$1 \;=\; N_p\left(\frac{1}{\mathcal{K}_{D,1}+N_p}+\frac{1}{\mathcal{K}_{D,2}+N_p}\right)\;. \tag{S12}$$

The solution to the previous equation is given by

$$N_p^2 = \mathcal{K}_{D,1}\mathcal{K}_{D,2}\;, \tag{S13}$$

which implies $\mathcal{K}_D = \sqrt{\mathcal{K}_{D,1}\mathcal{K}_{D,2}}$, or equivalently, $\mathcal{K}_A = \sqrt{\mathcal{K}_{A,1}\mathcal{K}_{A,2}}$. We have, thus, shown, that the affinity constant for this system is the geometric mean of the individual affinities. Since the binding score correlates with the affinity constant, the previous analysis based on chemical reactions between the peptide and the alleles leads to the following choice

$$\mathcal{I}''_q \;=\; \sqrt{\sigma_i\,\sigma'_i}\;, \tag{S14}$$

where $q = (a_i, a_{i'})$. With this choice for $\mathcal{I}''_q$, we can now compute $\mathcal{I}''$. We have

$$\begin{aligned}\mathcal{I}'' &= \frac{(f_1^2+\epsilon)\sigma_1 + 2(f_1 f_2-\epsilon)\sqrt{\sigma_1\,\sigma_2} + (f_2^2+\epsilon)\sigma_2}{(f_1+f_2)^2} \\ &= \frac{(f_1\sqrt{\sigma_1}+f_2\sqrt{\sigma_2})^2 + \epsilon(\sqrt{\sigma_1}-\sqrt{\sigma_2})^2}{(f_1+f_2)^2}\;.\end{aligned}$$

The previous equation shows that $\mathcal{I}'$" $\neq \mathcal{C}$, even in the absence of correlations (if $\epsilon = 0$), and that the mean individual regional coverage metric, $\mathcal{I}''$, depends on ϵ. The term in $\mathcal{I}''$ proportional to ϵ, $(\sqrt{\sigma_1}-\sqrt{\sigma_2})^2$, is always positive. This means $\mathcal{I}''_{\epsilon<0} < \mathcal{I}''_{\epsilon=0} < \mathcal{I}''_{\epsilon>0}$, which in turn implies that (contrary to the previous choice for $\mathcal{I}'_q$ is defined by Eq. (S6)) if there is a positive correlation for homozygous alleles, so that $\epsilon > 0$, and therefore a negative correlation for heterozygous alleles, the mean individual regional coverage metric, $\mathcal{I}''$, will be greater than in the case when $\epsilon < 0$, and there is a negative correlation for homozygous alleles (and therefore a positive correlation for heterozygous alleles). Our results indicate that if the individual regional coverage metric, $\mathcal{I}''_q$, is defined by Eq. (S14), then the presence of a positive (negative) correlation for homozygous alleles leads to a greater (lower) value for the mean individual regional coverage metric, $\mathcal{I}''$, when compared to $\mathcal{I}''_{\epsilon=0}$; that is, neglecting the presence of a positive (negative) correlation for homozygous alleles leads to under (over) estimating the value of the mean individual regional coverage metric.

2 ANALYSIS OF IMMUNO-DOMINANT EPITOPES: PROBABILITY DISTRIBUTIONS FOR g_j AND ϕ_j

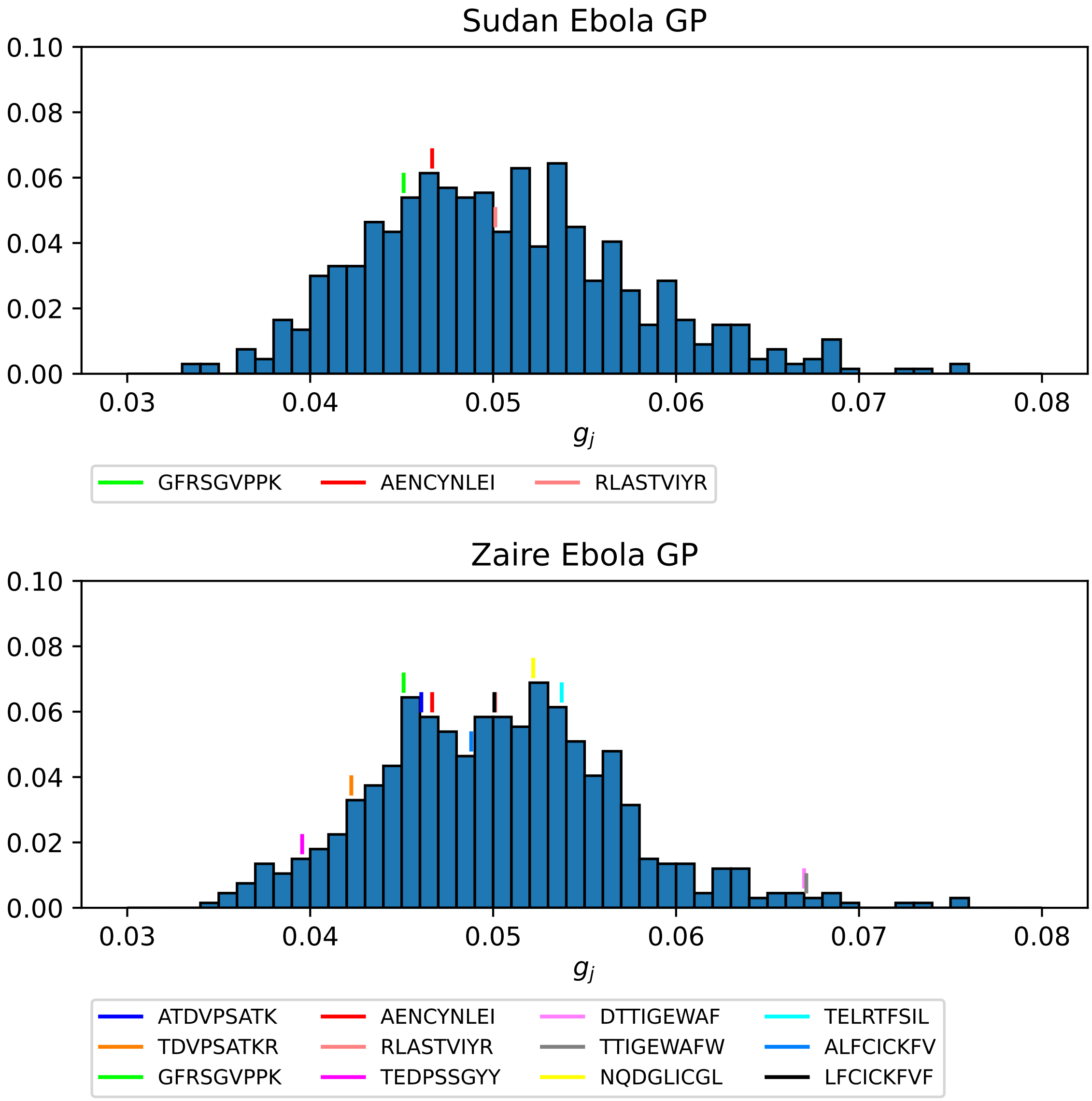

Figure S1. Top: Ebola GP (Sudan), Bottom: Ebola GP (Zaire)

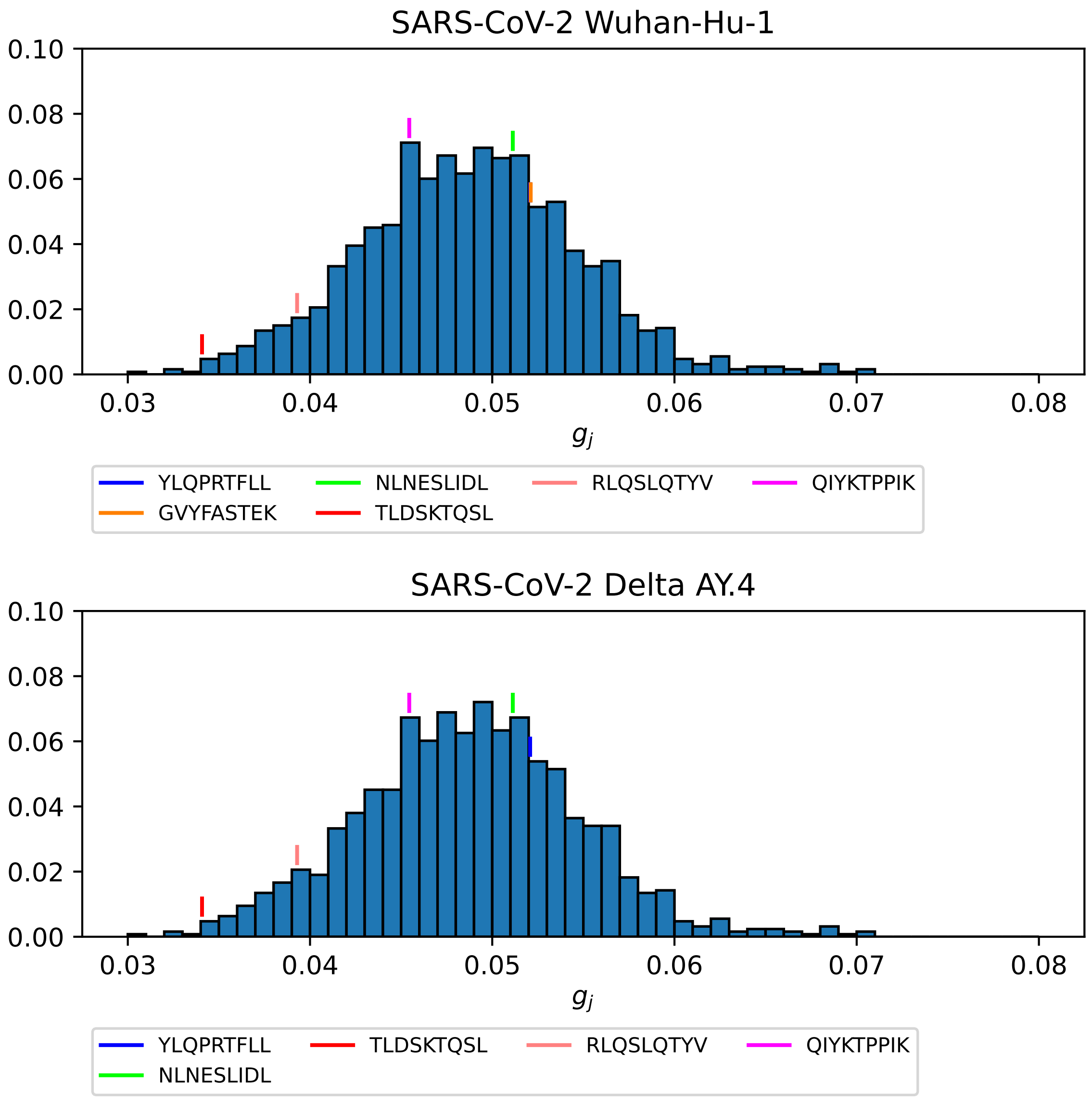

Figure S2. Top: SARS-CoV-2 Wuhan-Hu-1, Bottom: SARS-CoV-2 Delta AY.4

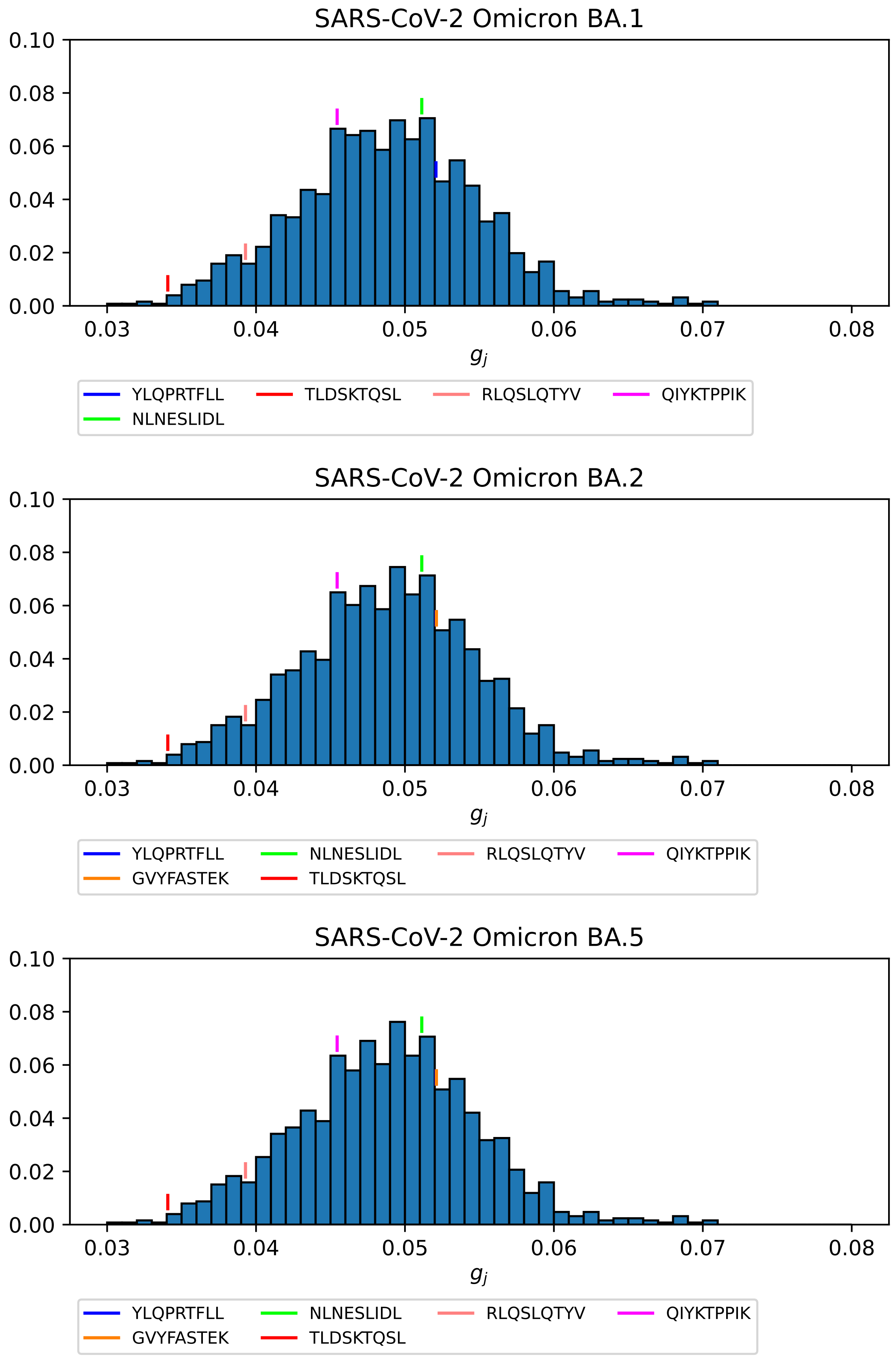

Figure S3. Top: SARS-CoV-2 Omicron BA.1, Middle: SARS-CoV-2 Omicron BA.2, Bottom: SARS-CoV-2 Omicron BA.5

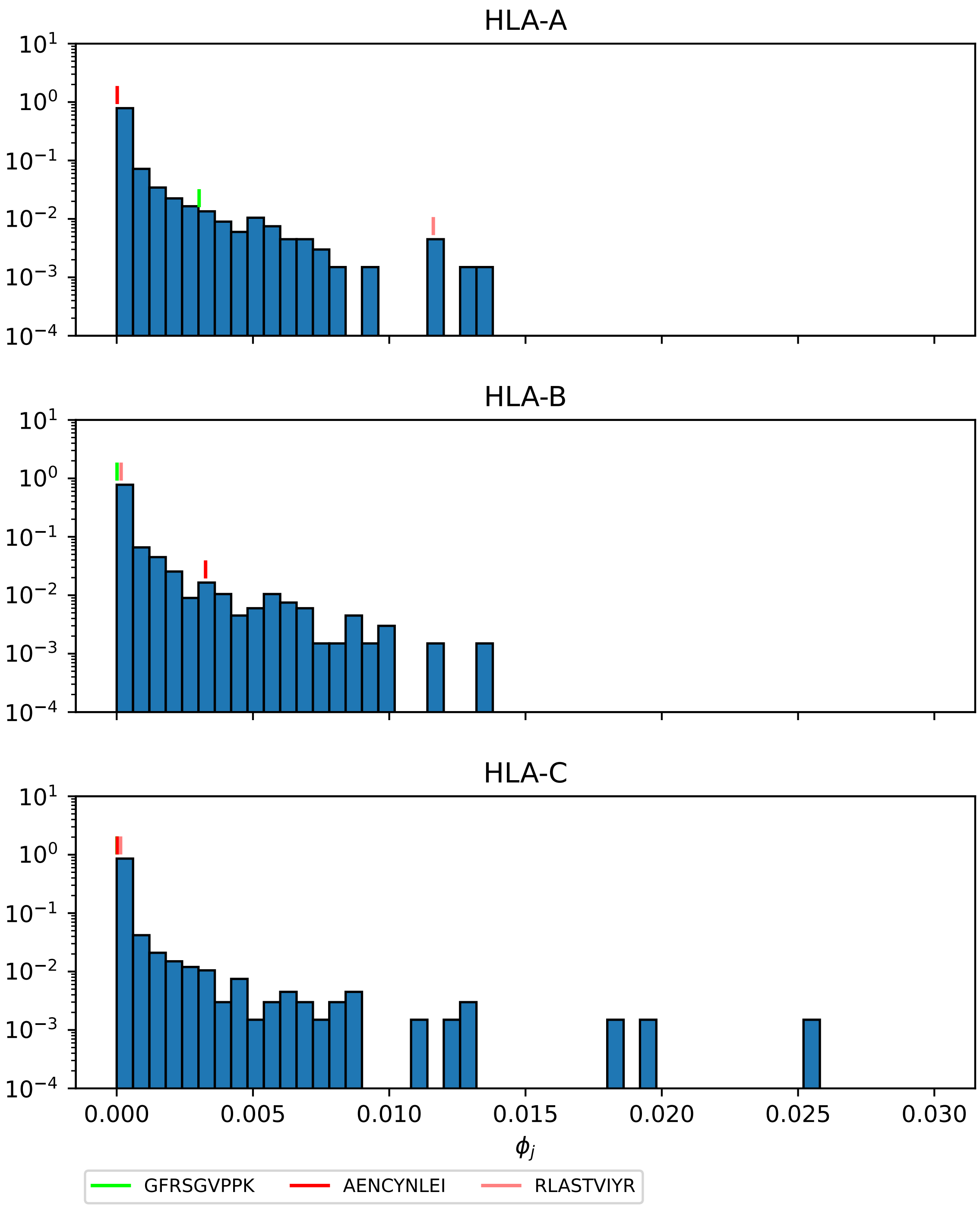

Figure S4. North America, Ebola GP (Sudan).

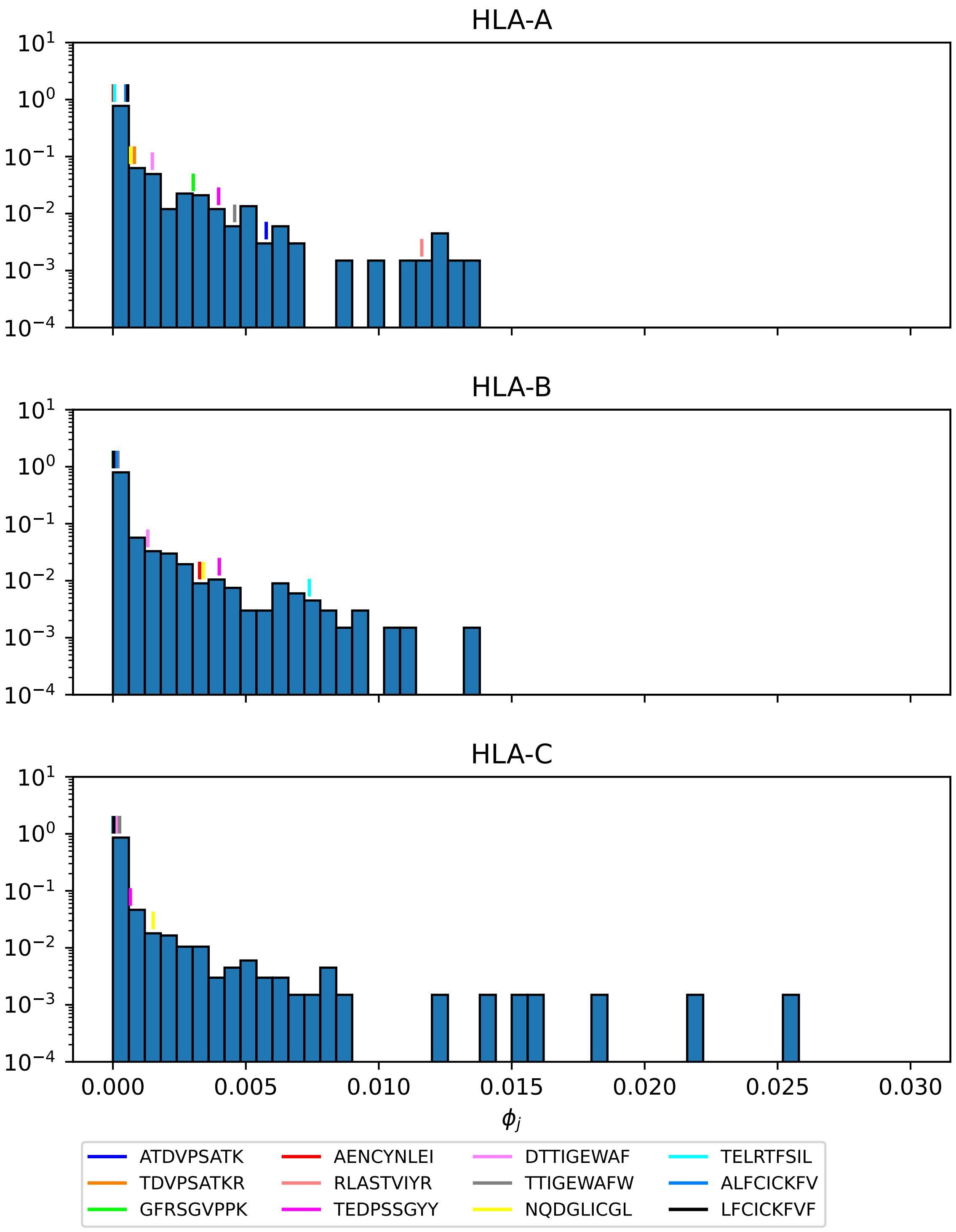

Figure S5. North America, Ebola GP (Zaire).

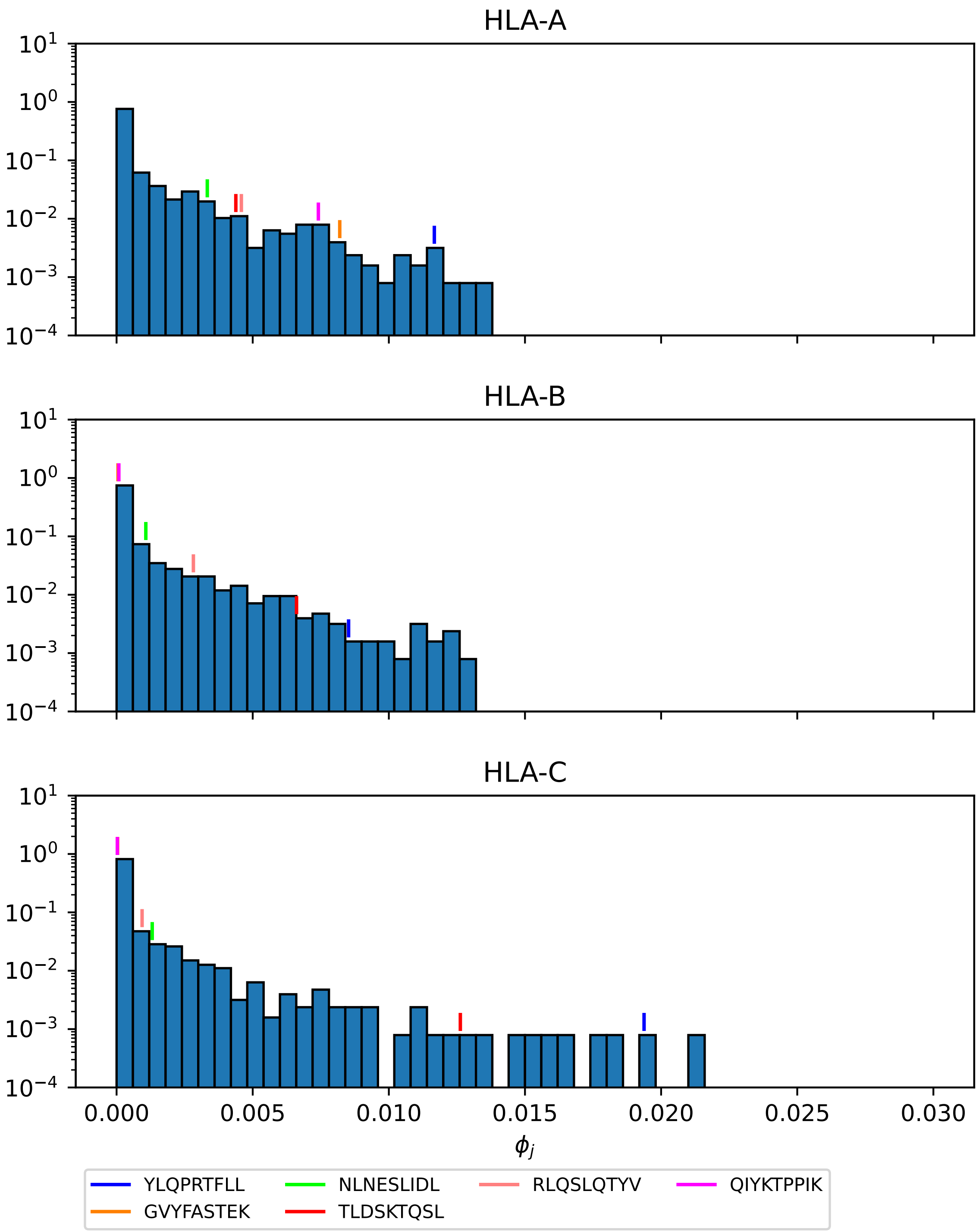

Figure S6. North America, SARS-CoV-2 Wuhan-Hu-1.

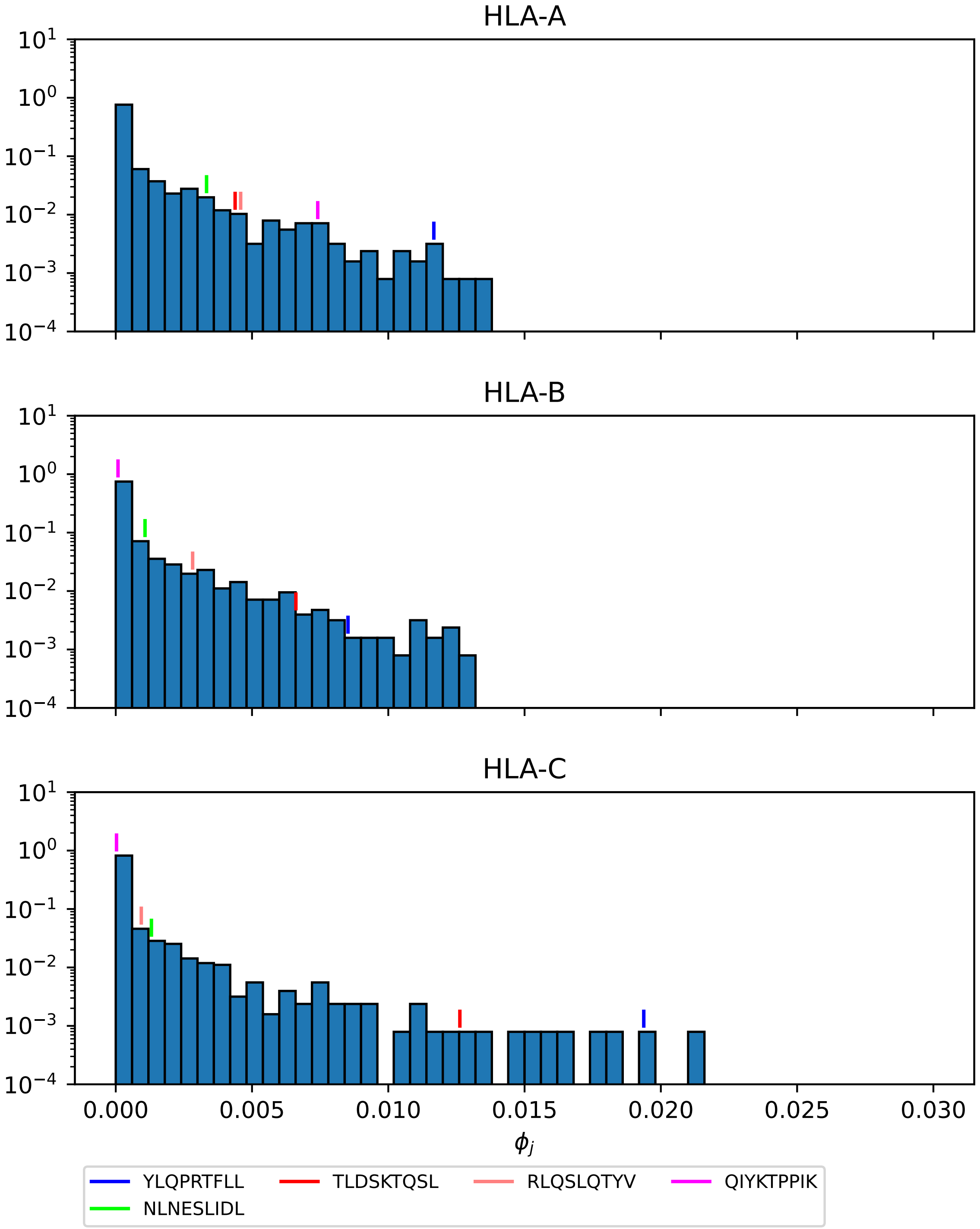

Figure S7. North America, SARS-CoV-2 Delta AY.4.

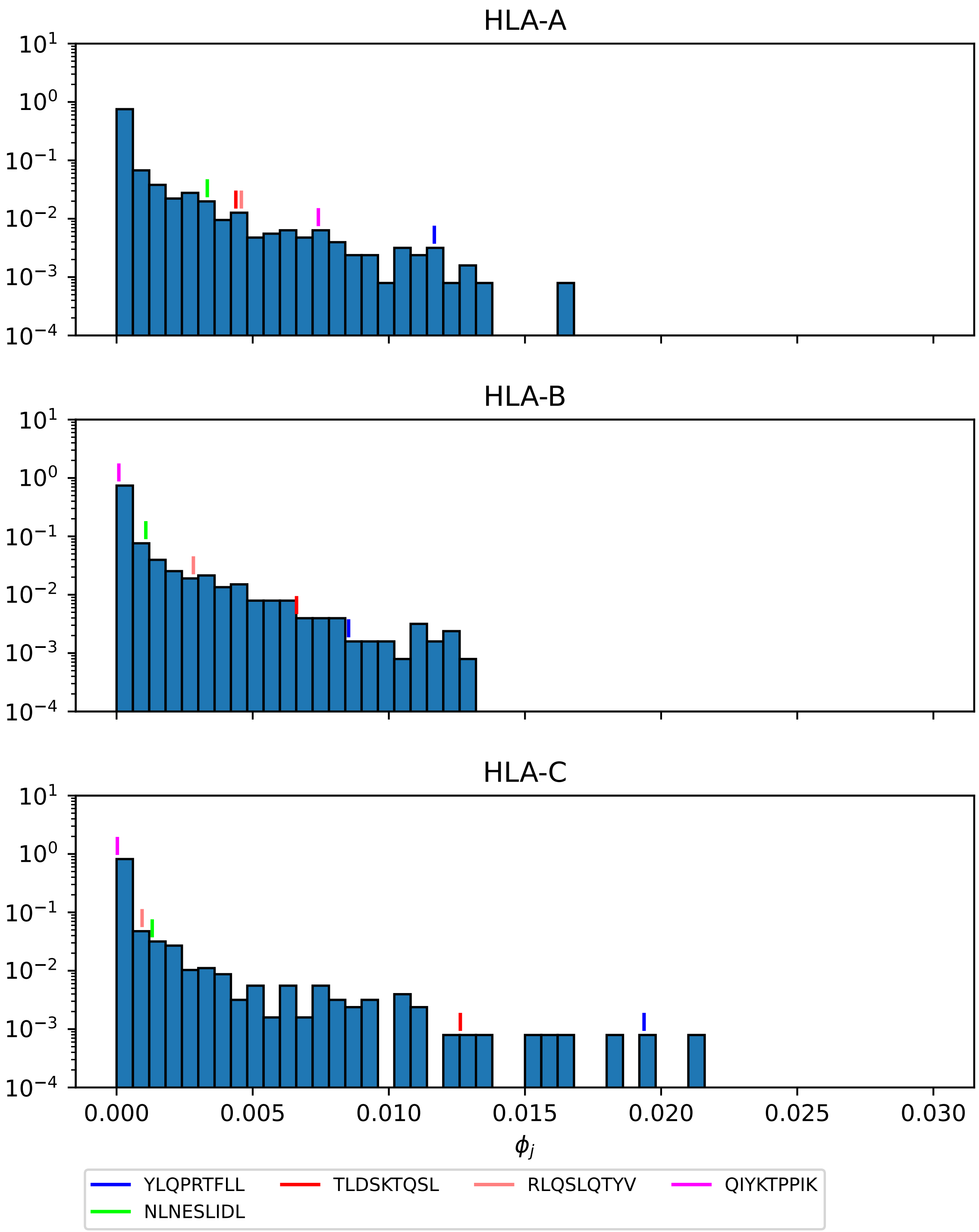

Figure S8. North America, SARS-CoV-2 Omicron BA.1.

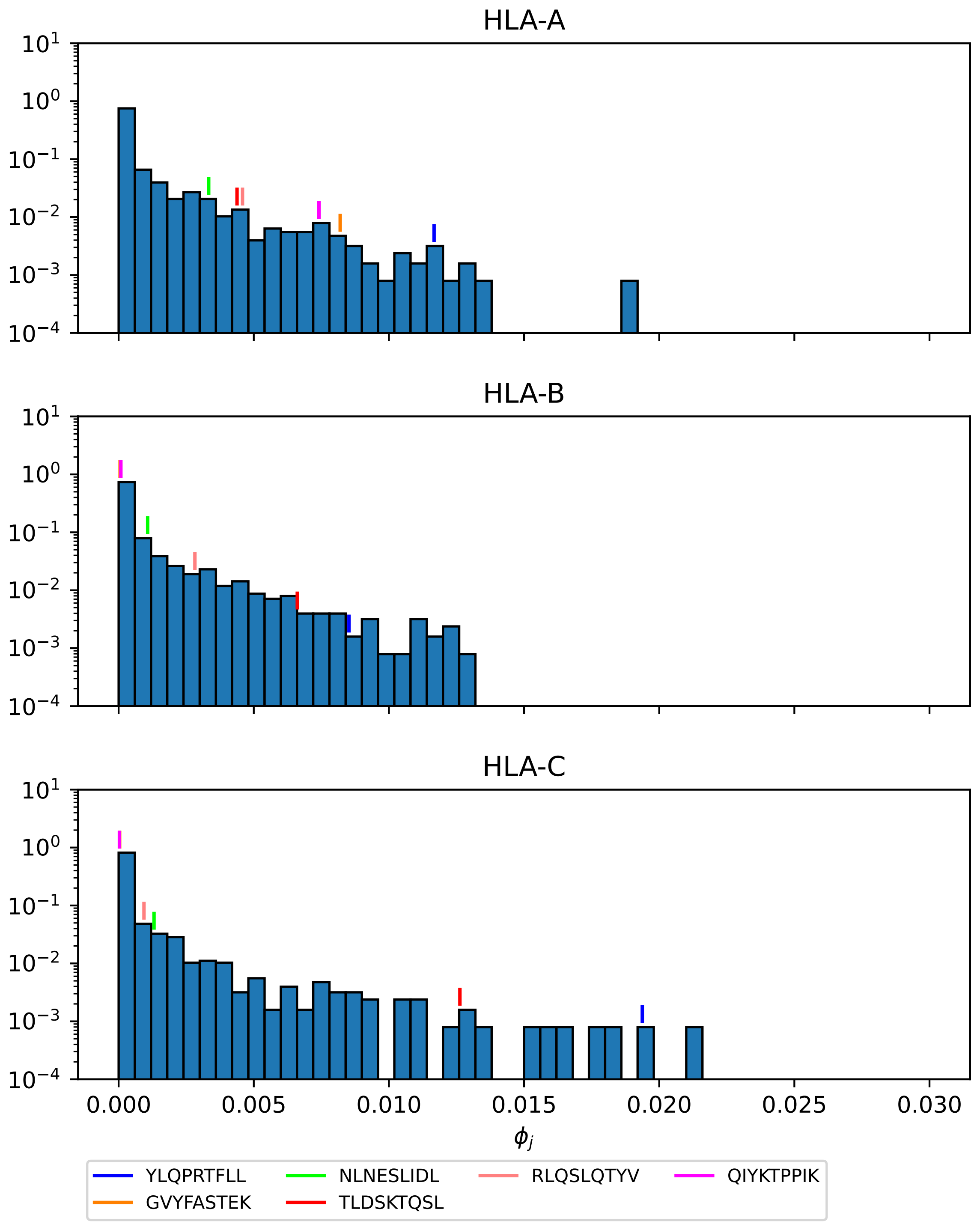

Figure S9. North America, SARS-CoV-2 Omicron BA.2.

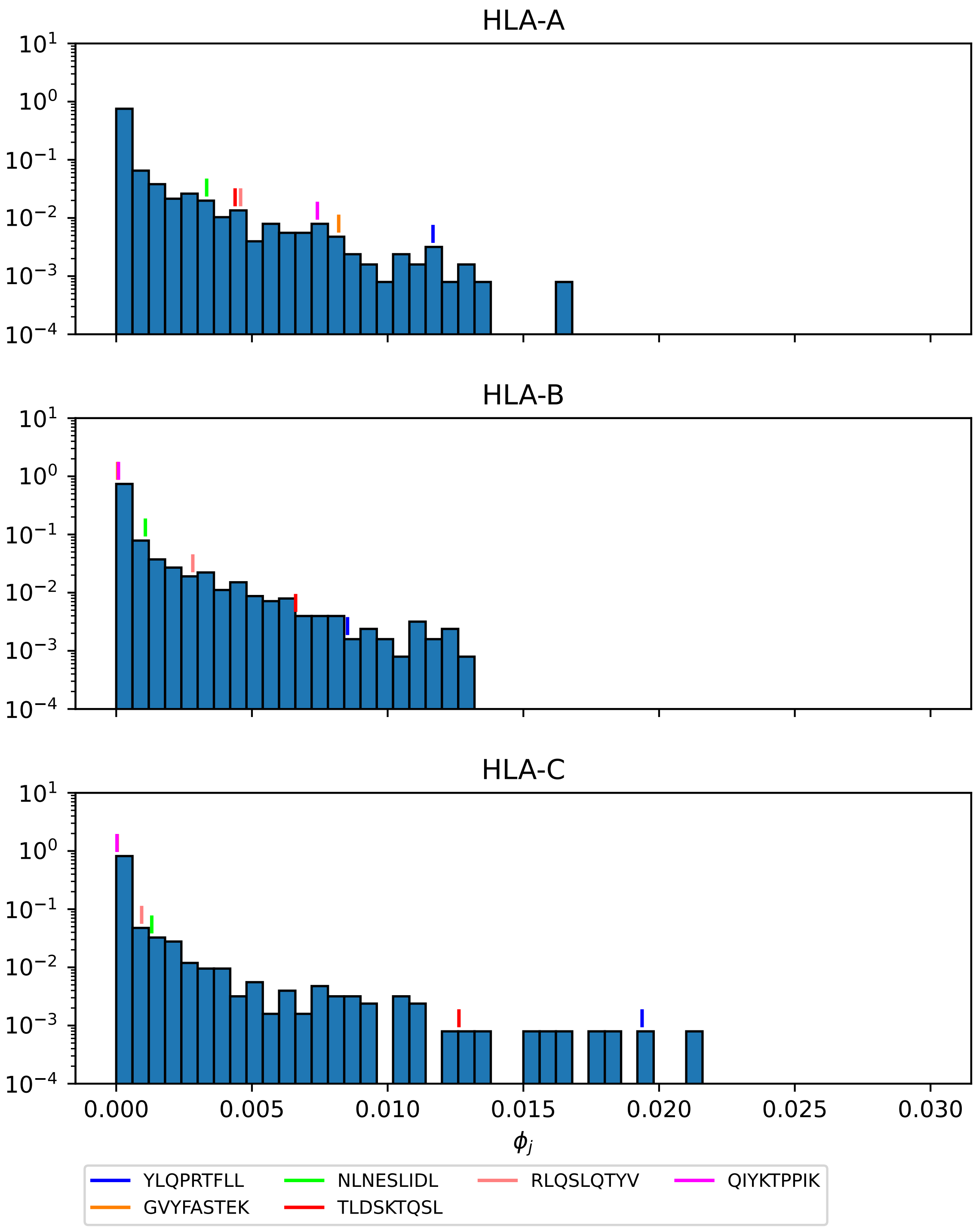

Figure S10. North America, SARS-CoV-2 Omicron BA.5.

3 DISSECTING THE MEAN REGIONAL COVERAGE METRIC: ALL REGIONS

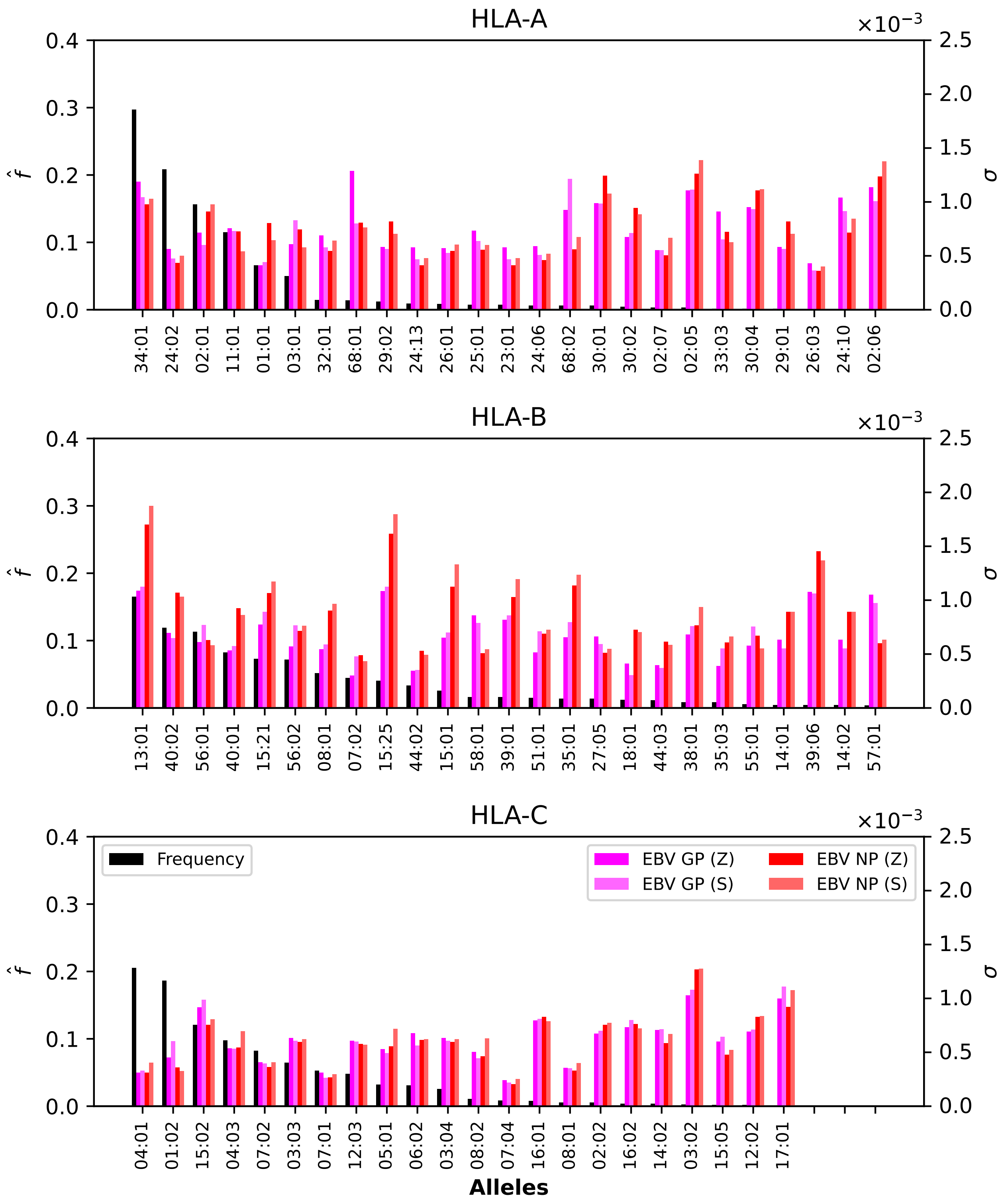

Figure S11. Normalized regional frequencies ($\hat{f}_i^{(1)}$) and Ebola σ_i values for the top 25 (22 for HLA-C) most frequent alleles of each type in Australia. The top panel represents HLA-A alleles, the middle HLA-B, and the bottom HLA-C. From left to right, the bars in each group represent frequency, Ebola GP1 (Zaire), Ebola GP1 (Sudan), Ebola NP (Zaire), and Ebola NP (Sudan).

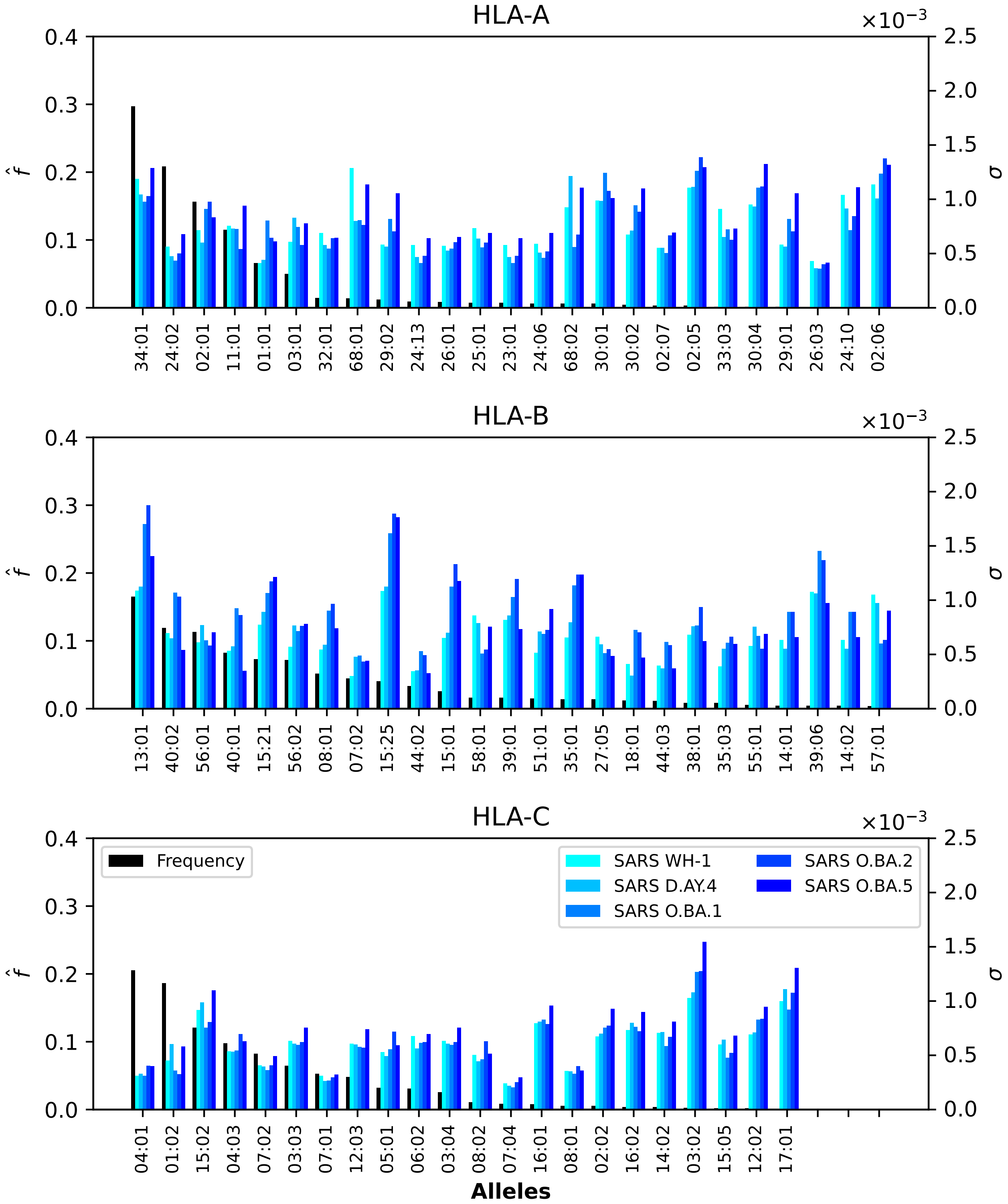

Figure S12. Normalized regional frequencies ($\hat{f}_i^{(1)}$) and SARS-CoV-2 σ_i values for the top 25 (22 for HLA-C) most frequent alleles of each type in Australia. The top panel represents HLA-A alleles, the middle HLA-B, and the bottom HLA-C. From left to right, the bars in each group represent frequency, SARS-CoV-2 Wuhan-Hu-1, SARS-CoV-2 Delta AY.4, SARS-CoV-2 Omicron BA.1, SARS-CoV-2 Omicron BA.2, SARS-CoV-2 Omicron BA.5.

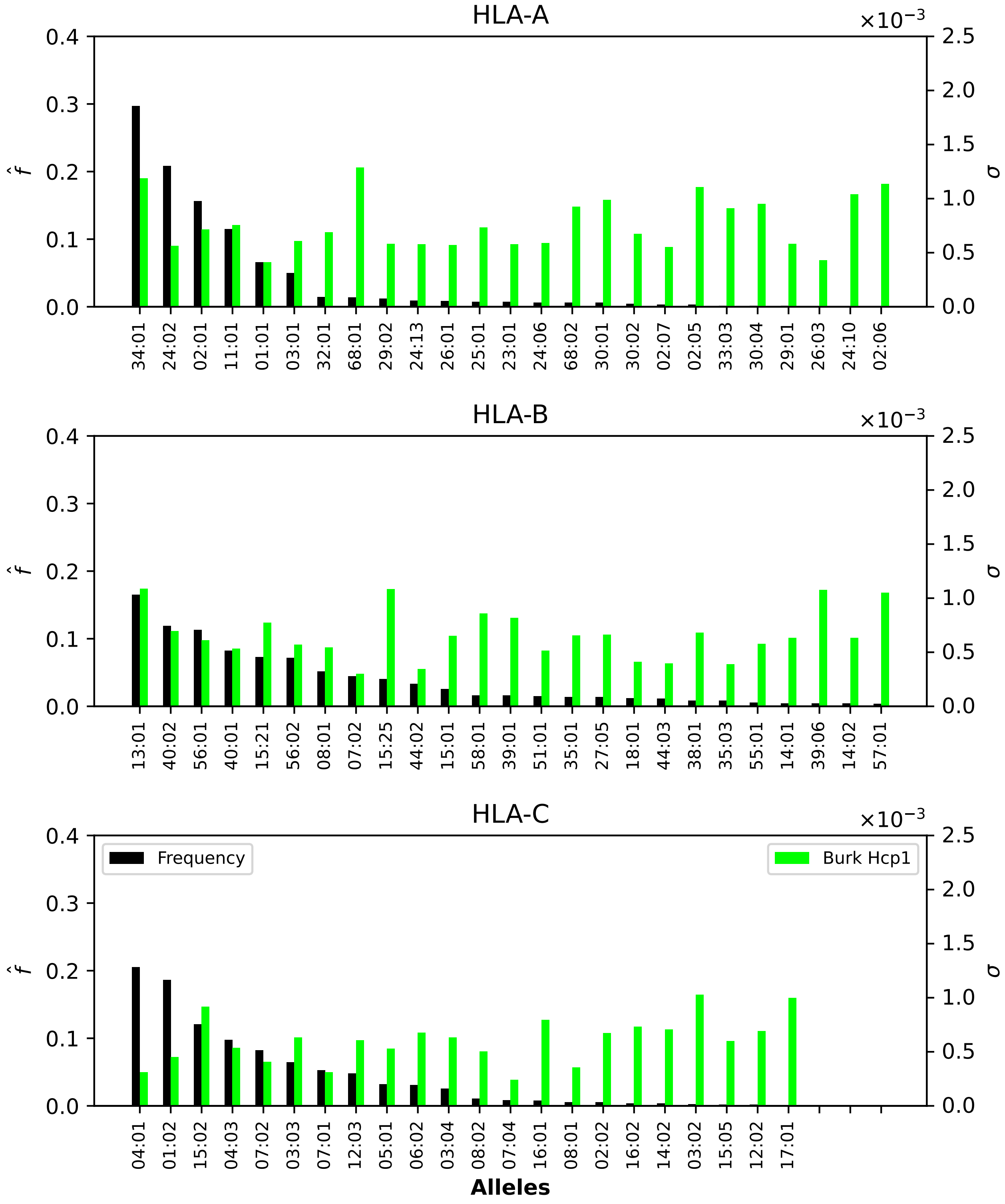

Figure S13. Normalized regional frequencies ($\hat{f}_i^{(1)}$) and Burkholderia σ_i values for the top 25 (22 for HLA-C) most frequent alleles of each type in Australia. The top panel represents HLA-A alleles, the middle HLA-B, and the bottom HLA-C. From left to right, the bars in each group represent frequency and Burkholderia Hcp1.

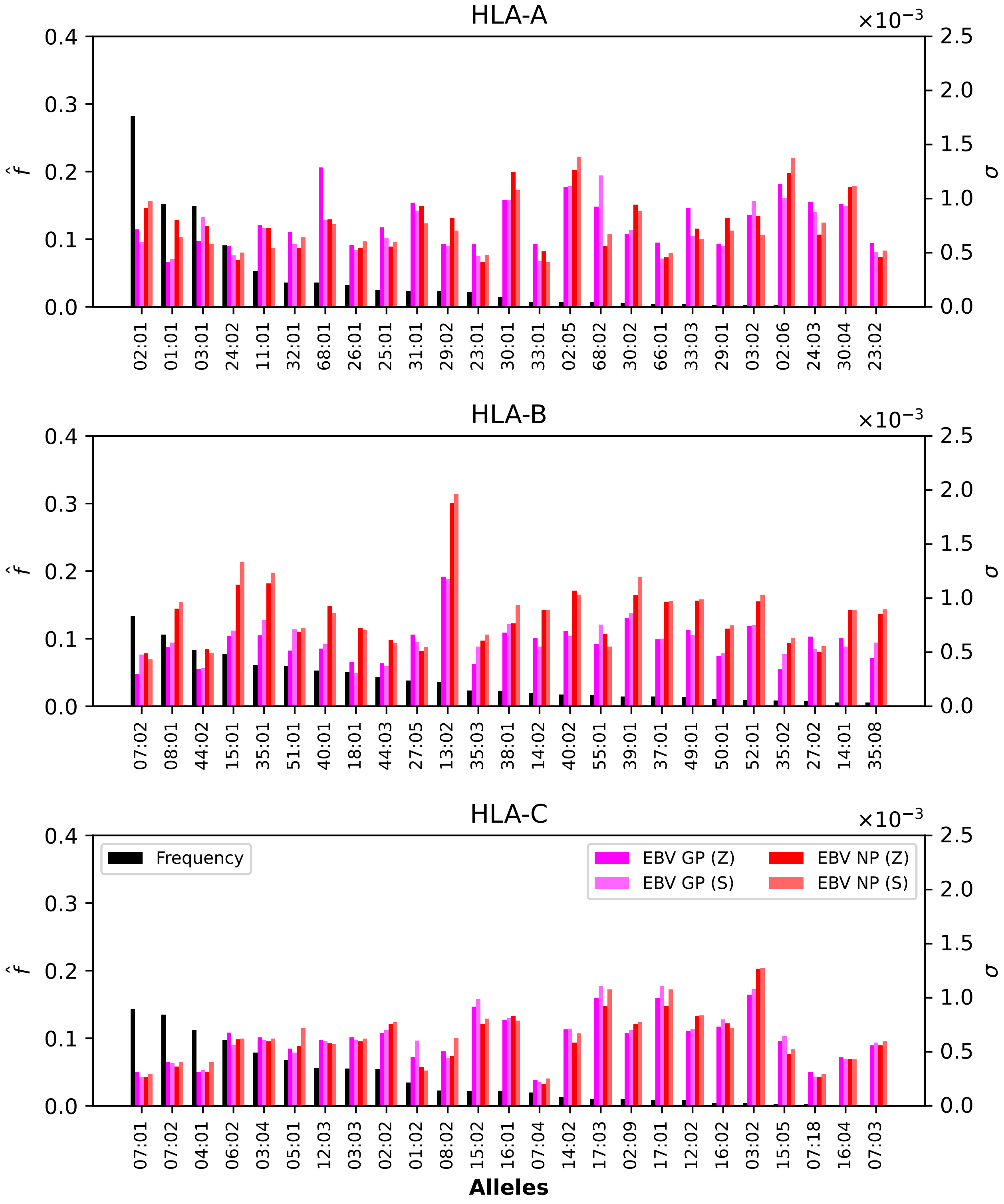

Figure S14. Normalized regional frequencies ($\hat{f}_i^{(2)}$) and Ebola σ_i values for the top 25 most frequent alleles of each type in Europe. The top panel represents HLA-A alleles, the middle HLA-B, and the bottom HLA-C. From left to right, the bars in each group represent frequency, Ebola GP1 (Zaire), Ebola GP1 (Sudan), Ebola NP (Zaire), and Ebola NP (Sudan).

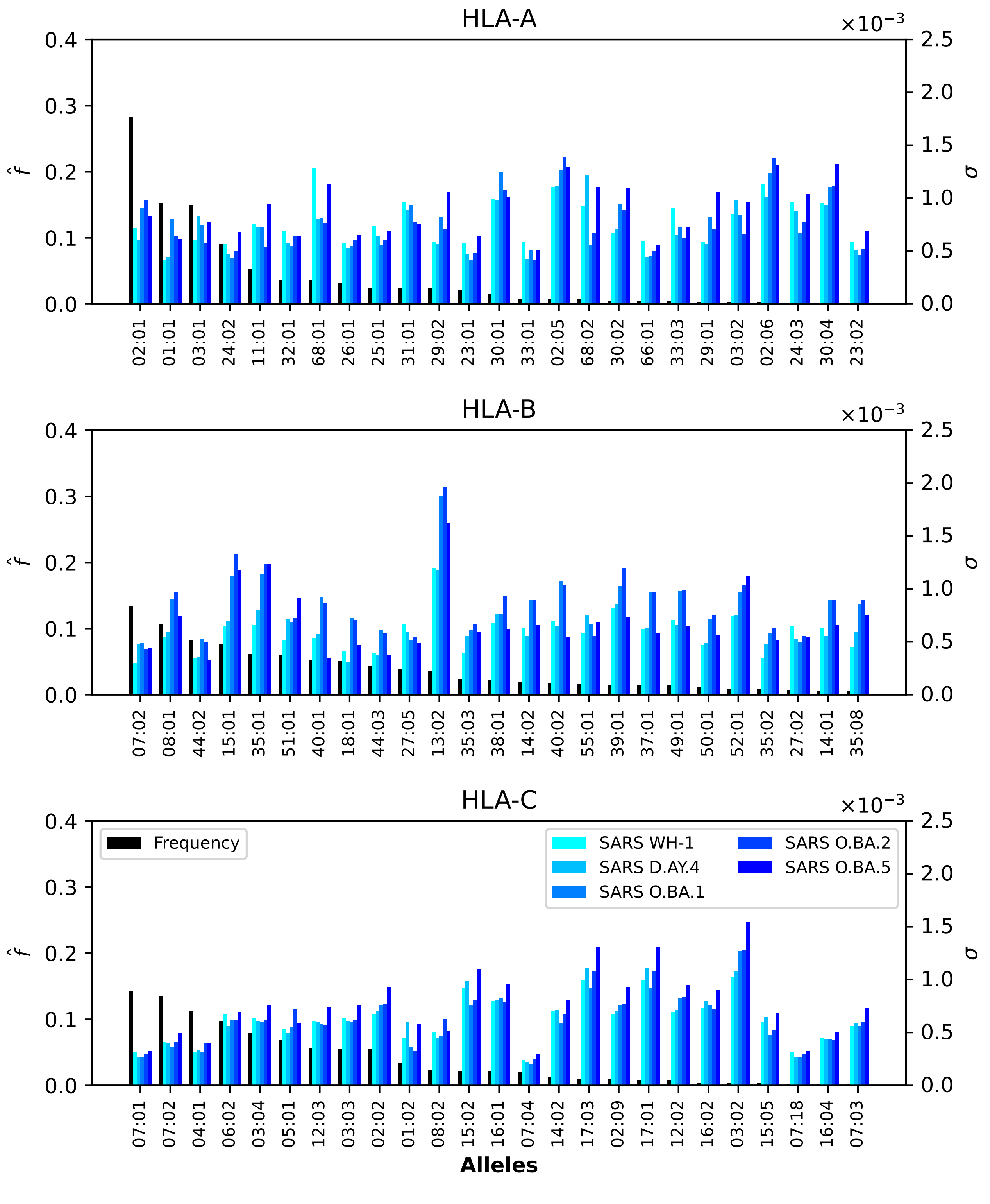

Figure S15. Normalized regional frequencies ($\hat{f}_i^{(2)}$) and SARS-CoV-2 σ_i values for the top 25 most frequent alleles of each type in Europe. The top panel represents HLA-A alleles, the middle HLA-B, and the bottom HLA-C. From left to right, the bars in each group represent frequency, SARS-CoV-2 Wuhan-Hu-1, SARS-CoV-2 Delta AY.4, SARS-CoV-2 Omicron BA.1, SARS-CoV-2 Omicron BA.2, SARS-CoV-2 Omicron BA.5.

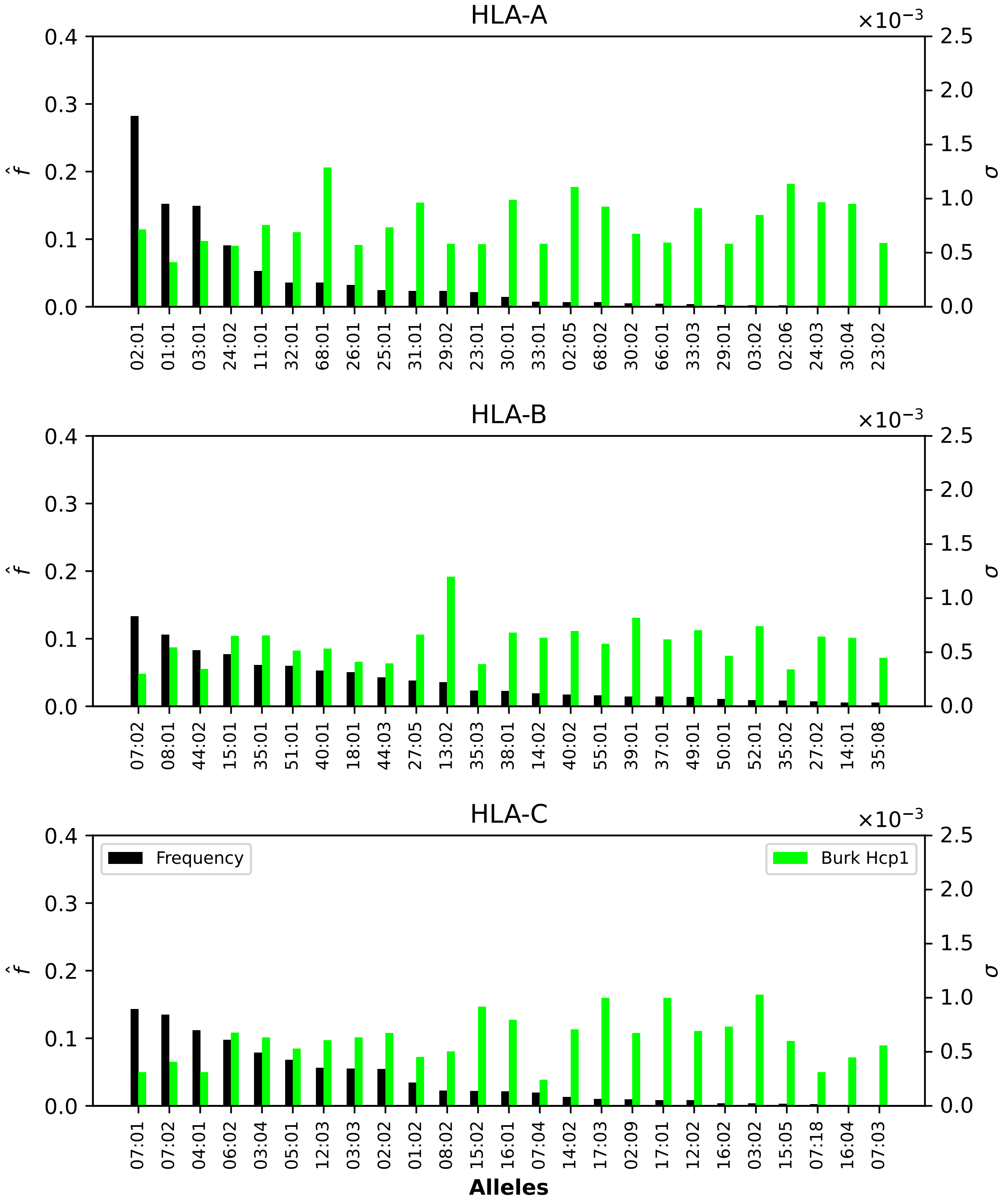

Figure S16. Normalized regional frequencies ($\hat{f}_i^{(2)}$) and Burkholderia σ_i values for the top 25 most frequent alleles of each type in Europe. The top panel represents HLA-A alleles, the middle HLA-B, and the bottom HLA-C. From left to right, the bars in each group represent frequency and Burkholderia Hcp1.

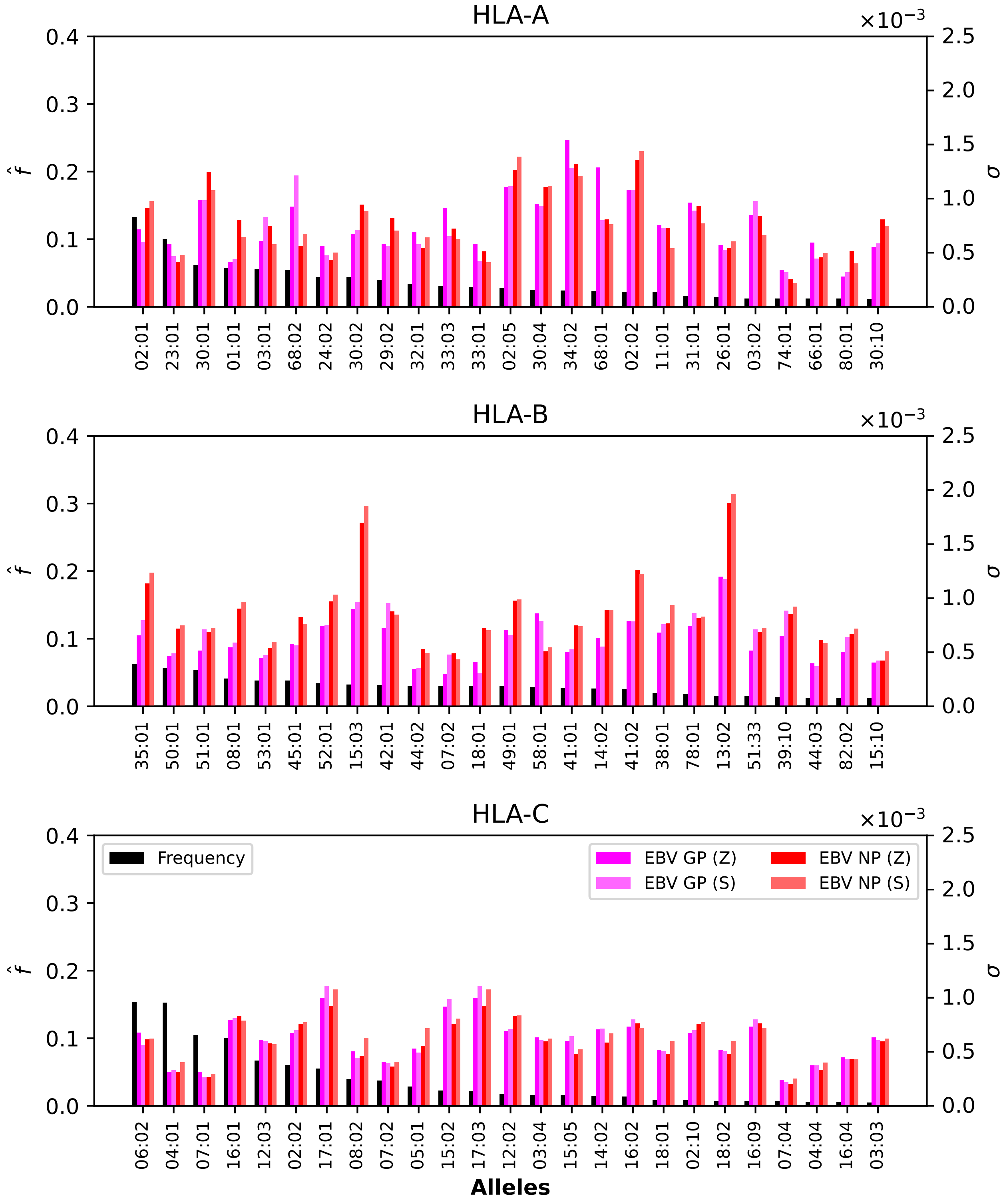

Figure S17. Normalized regional frequencies ($\hat{f}_i^{(3)}$) and Ebola σ_i values for the top 25 most frequent alleles of each type in North Africa. The top panel represents HLA-A alleles, the middle HLA-B, and the bottom HLA-C. From left to right, the bars in each group represent frequency, Ebola GP1 (Zaire), Ebola GP1 (Sudan), Ebola NP (Zaire), and Ebola NP (Sudan).

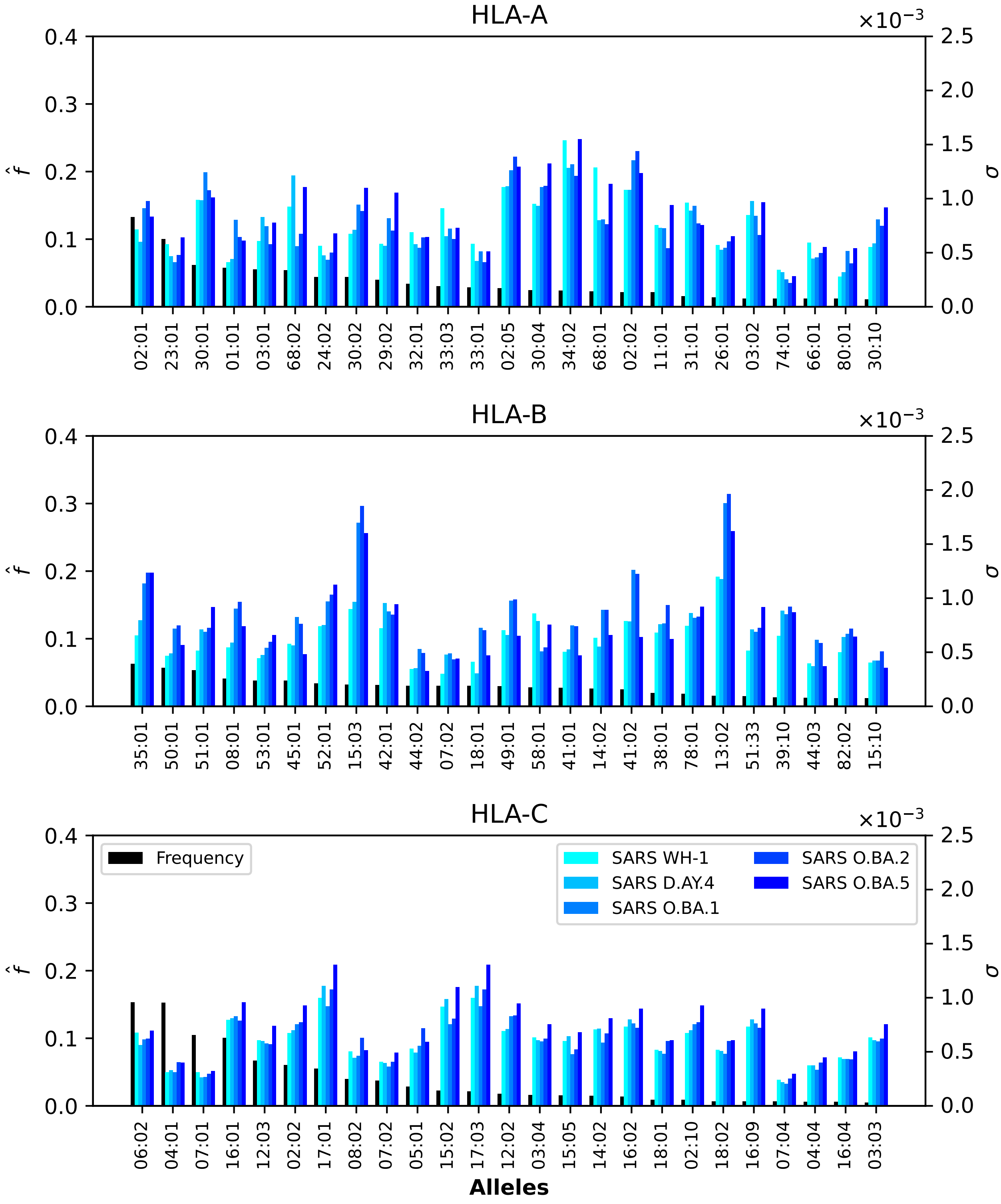

Figure S18. Normalized regional frequencies ($\hat{f}_i^{(3)}$) and SARS-CoV-2 σ_i values for the top 25 most frequent alleles of each type in North Africa. The top panel represents HLA-A alleles, the middle HLA-B, and the bottom HLA-C. From left to right, the bars in each group represent frequency, SARS-CoV-2 Wuhan-Hu-1, SARS-CoV-2 Delta AY.4, SARS-CoV-2 Omicron BA.1, SARS-CoV-2 Omicron BA.2, SARS-CoV-2 Omicron BA.5.

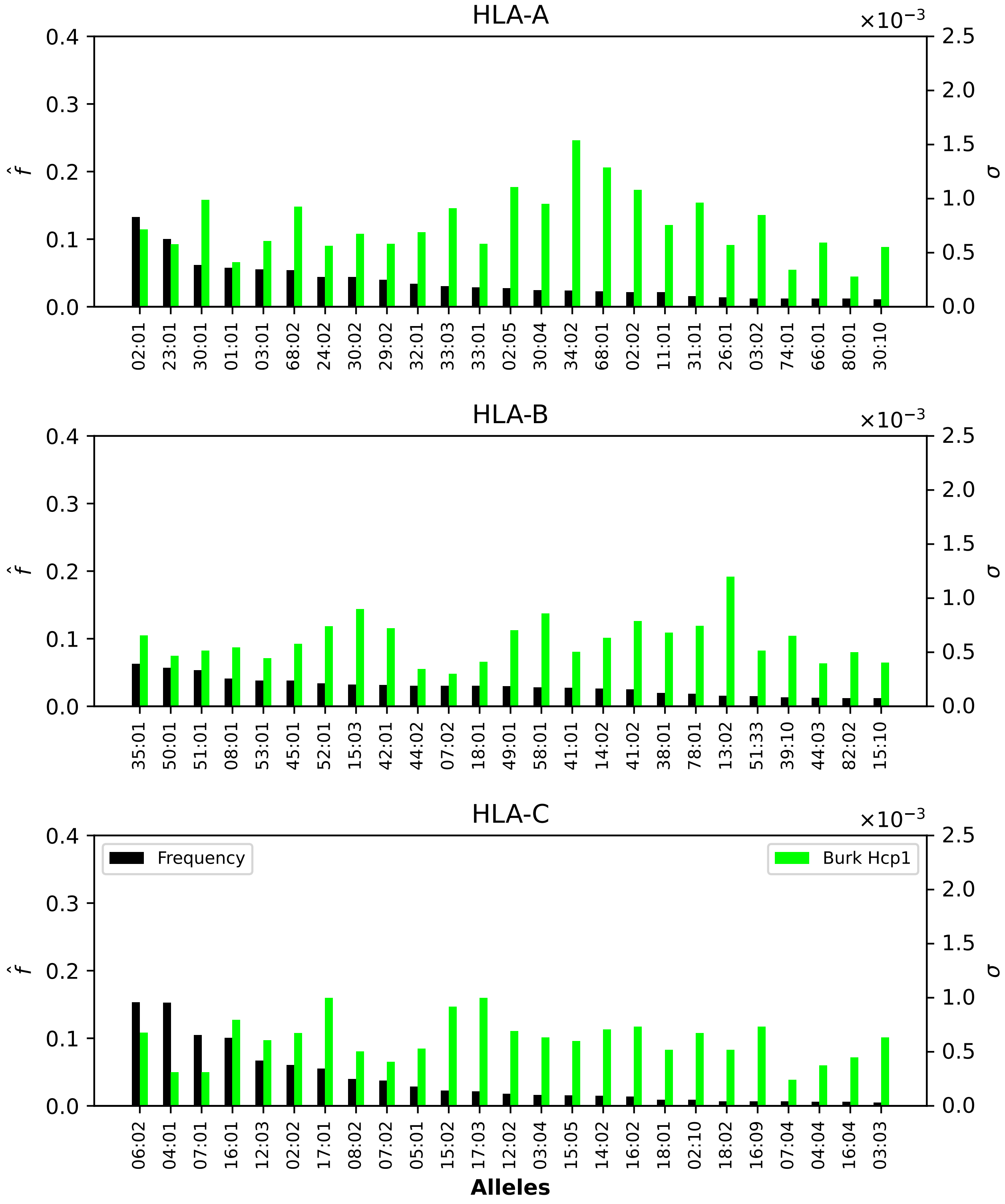

Figure S19. Normalized regional frequencies ($\hat{f}_i^{(3)}$) and Burkholderia σ_i values for the top 25 most frequent alleles of each type in North Africa. The top panel represents HLA-A alleles, the middle HLA-B, and the bottom HLA-C. From left to right, the bars in each group represent frequency and Burkholderia Hcp1.

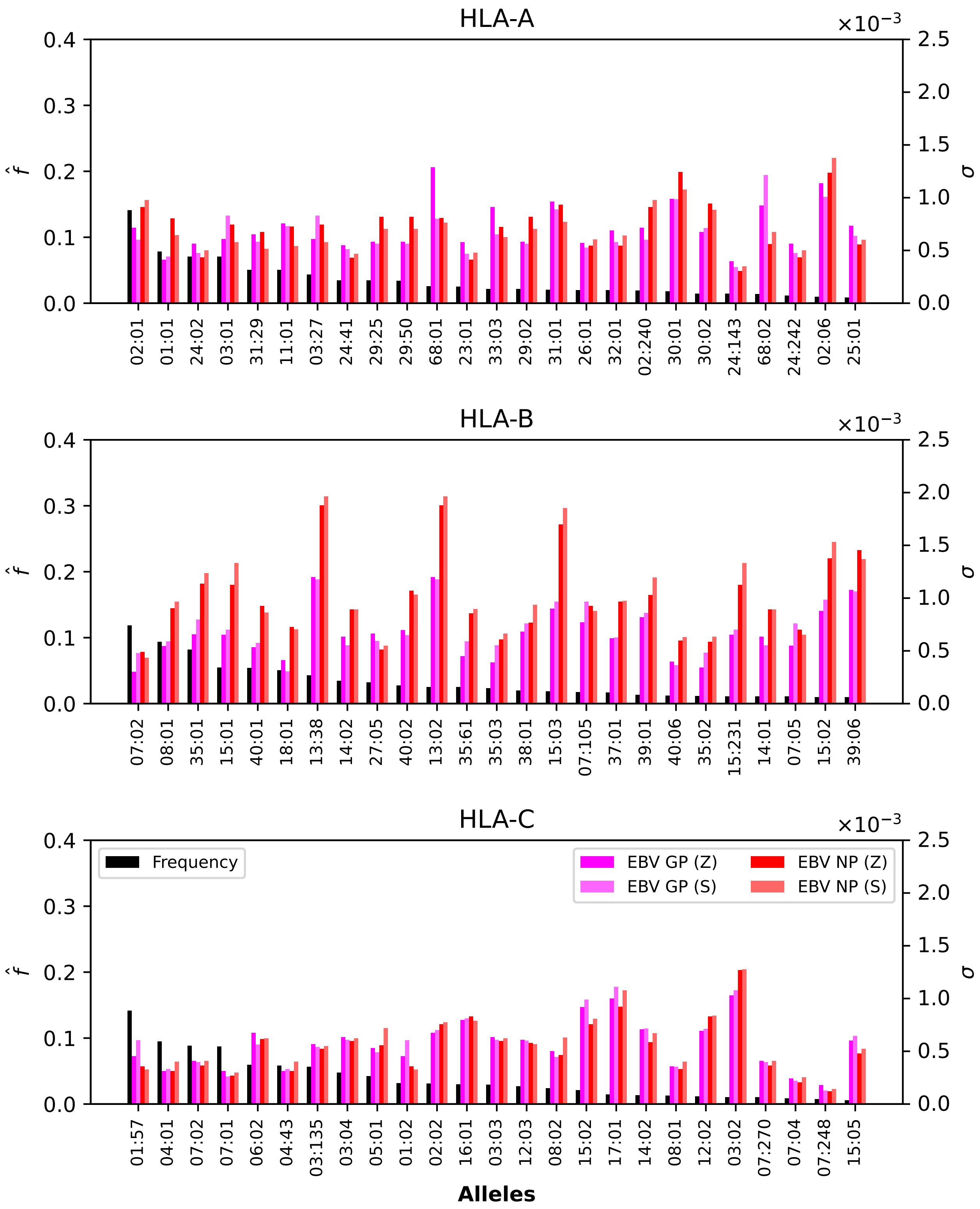

Figure S20. Normalized regional frequencies ($\hat{f}_i^{(4)}$) and Ebola σ_i values for the top 25 most frequent alleles of each type in North America. The top panel represents HLA-A alleles, the middle HLA-B, and the bottom HLA-C. From left to right, the bars in each group represent frequency, Ebola GP1 (Zaire), Ebola GP1 (Sudan), Ebola NP (Zaire), and Ebola NP (Sudan).

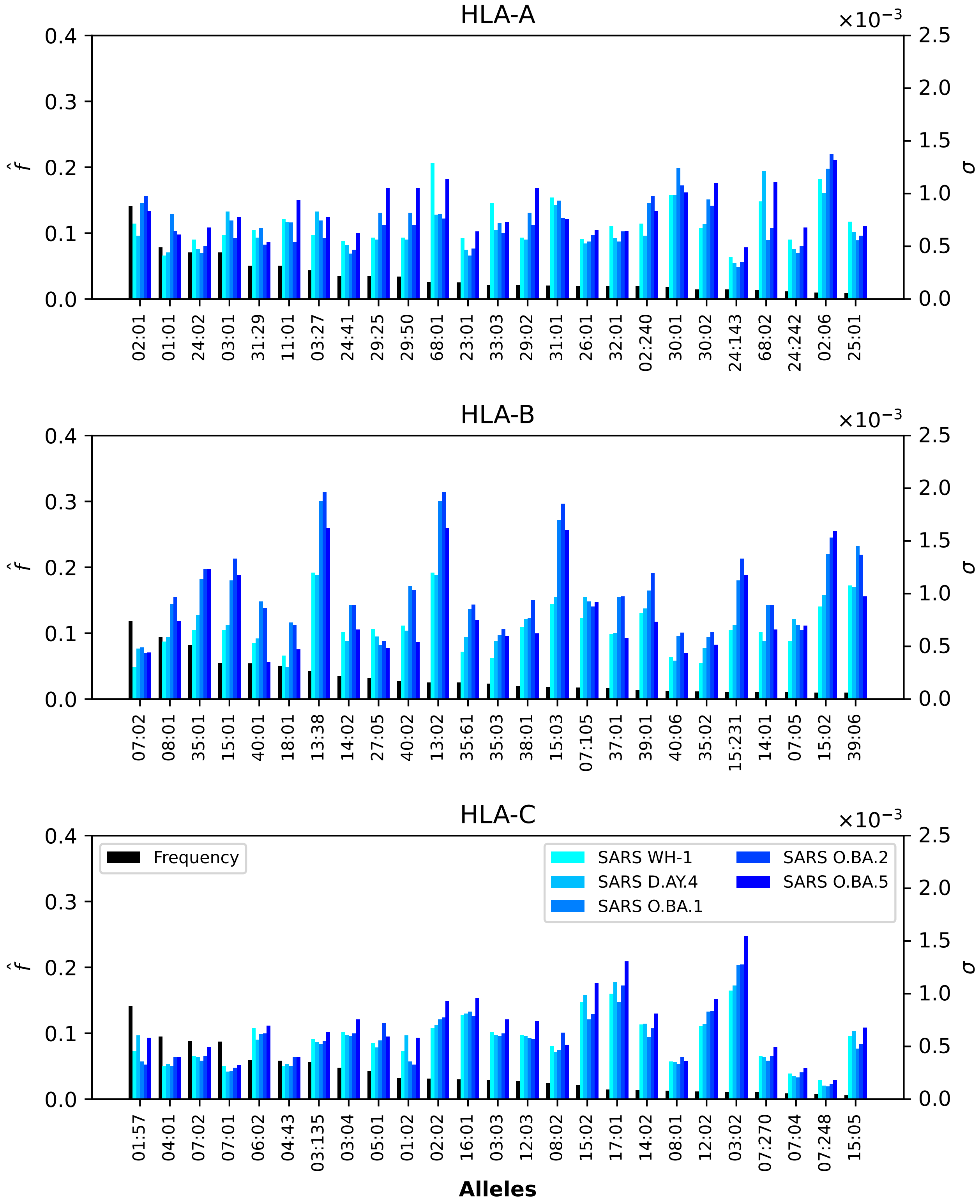

Figure S21. Normalized regional frequencies ($\hat{f}_i^{(4)}$) and SARS-CoV-2 σ_i values for the top 25 most frequent alleles of each type in North America. The top panel represents HLA-A alleles, the middle HLA-B, and the bottom HLA-C. From left to right, the bars in each group represent frequency, SARS-CoV-2 Wuhan-Hu-1, SARS-CoV-2 Delta AY.4, SARS-CoV-2 Omicron BA.1, SARS-CoV-2 Omicron BA.2, SARS-CoV-2 Omicron BA.5.

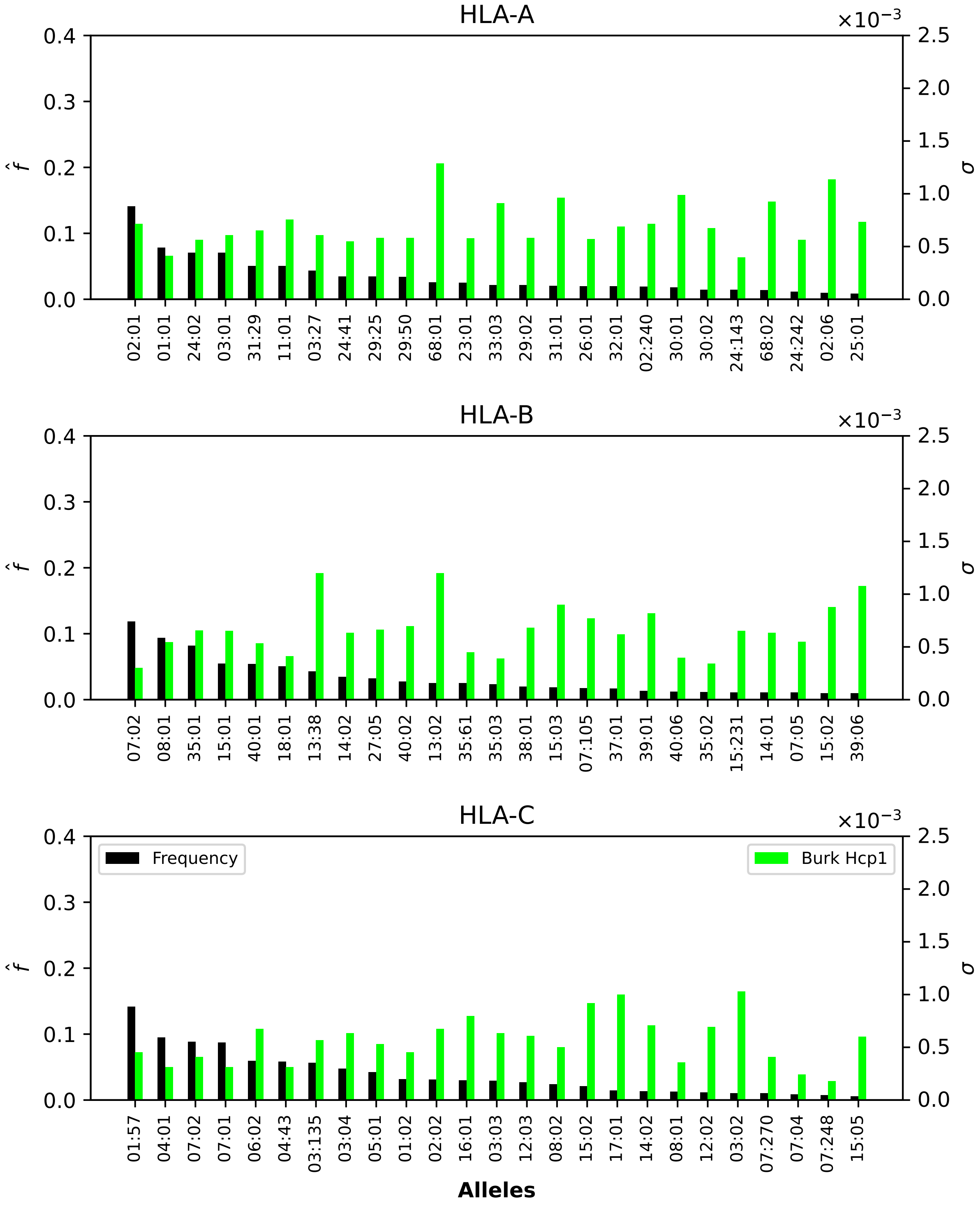

Figure S22. Normalized regional frequencies ($\hat{f}_i^{(4)}$) and Burkholderia σ_i values for the top 25 most frequent alleles of each type in North America. The top panel represents HLA-A alleles, the middle HLA-B, and the bottom HLA-C. From left to right, the bars in each group represent frequency and Burkholderia Hcp1.

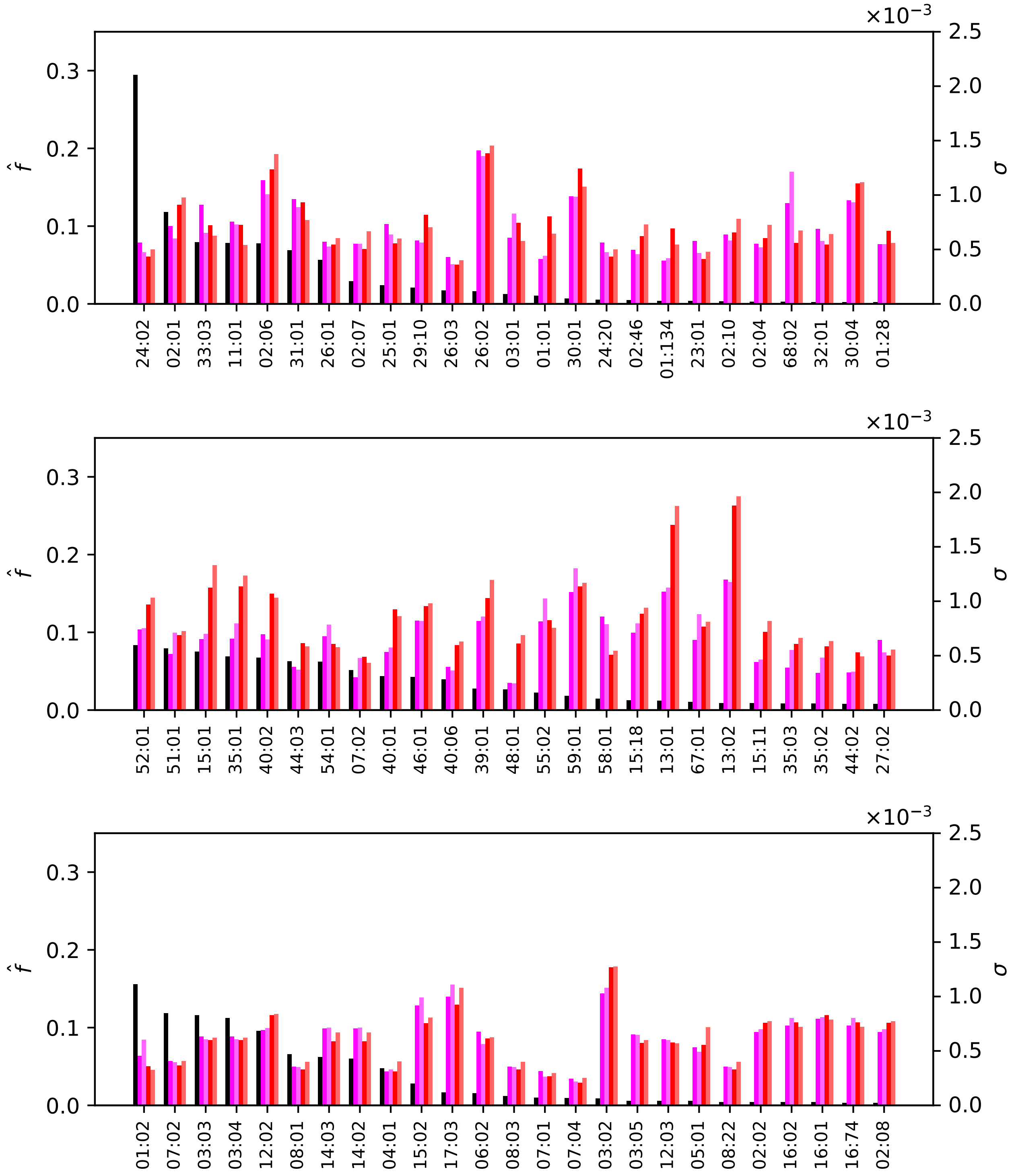

Figure S23. Normalized regional frequencies ($\hat{f}_i^{(5)}$) and Ebola σ_i values for the top 25 most frequent alleles of each type in Northeast Asia. The top panel represents HLA-A alleles, the middle HLA-B, and the bottom HLA-C. From left to right, the bars in each group represent frequency, Ebola GP1 (Zaire), Ebola GP1 (Sudan), Ebola NP (Zaire), and Ebola NP (Sudan).

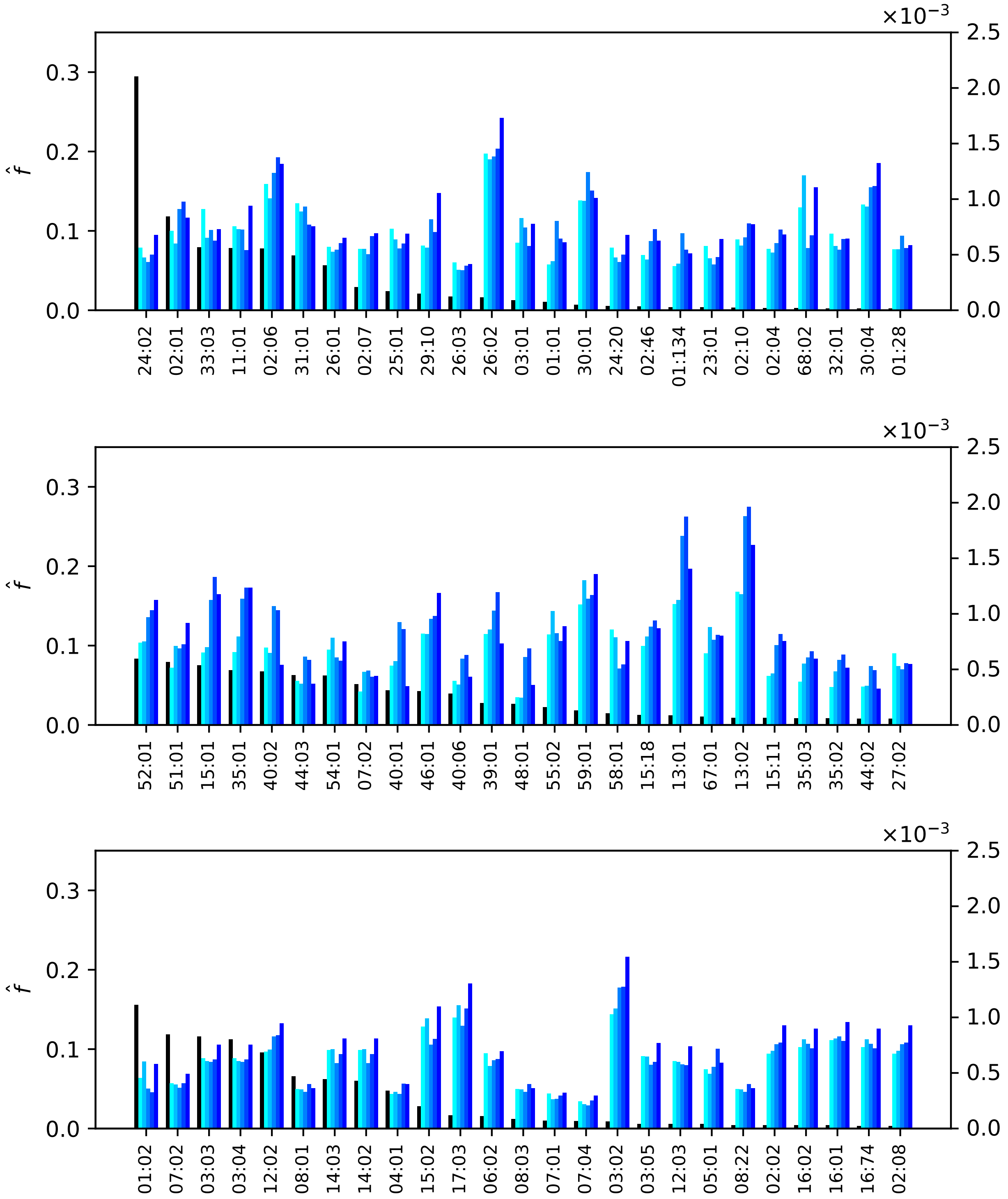

Figure S24. Normalized regional frequencies ($\hat{f}_i^{(5)}$) and SARS-CoV-2 σ_i values for the top 25 most frequent alleles of each type in Northeast Asia. The top panel represents HLA-A alleles, the middle HLA-B, and the bottom HLA-C. From left to right, the bars in each group represent frequency, SARS-CoV-2 Wuhan-Hu-1, SARS-CoV-2 Delta AY.4, SARS-CoV-2 Omicron BA.1, SARS-CoV-2 Omicron BA.2, SARS-CoV-2 Omicron BA.5.

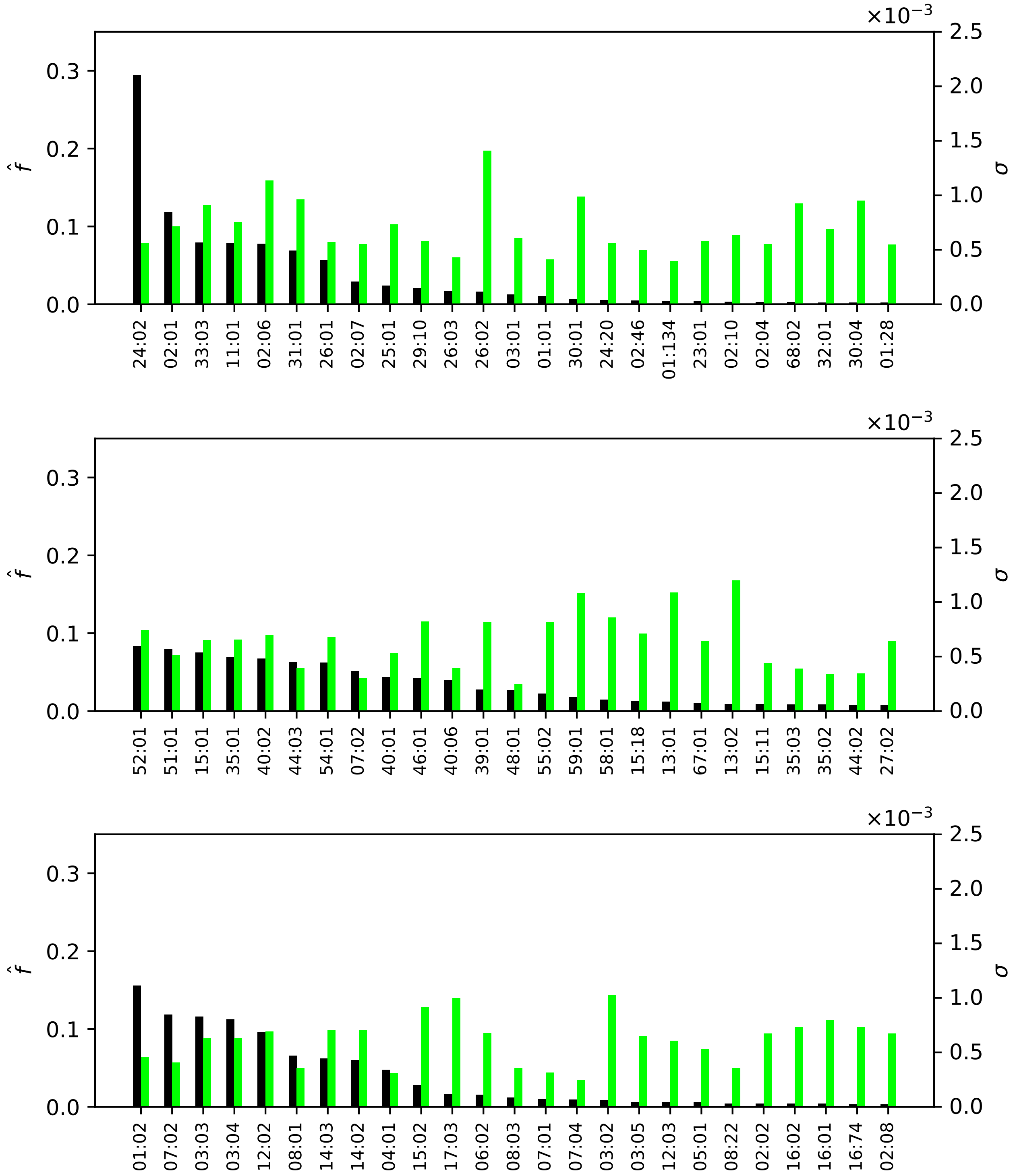

Figure S25. Normalized regional frequencies ($\hat{f}_i^{(5)}$) and Burkholderia σ_i values for the top 25 most frequent alleles of each type in Northeast Asia. The top panel represents HLA-A alleles, the middle HLA-B, and the bottom HLA-C. From left to right, the bars in each group represent frequency and Burkholderia Hcp1.

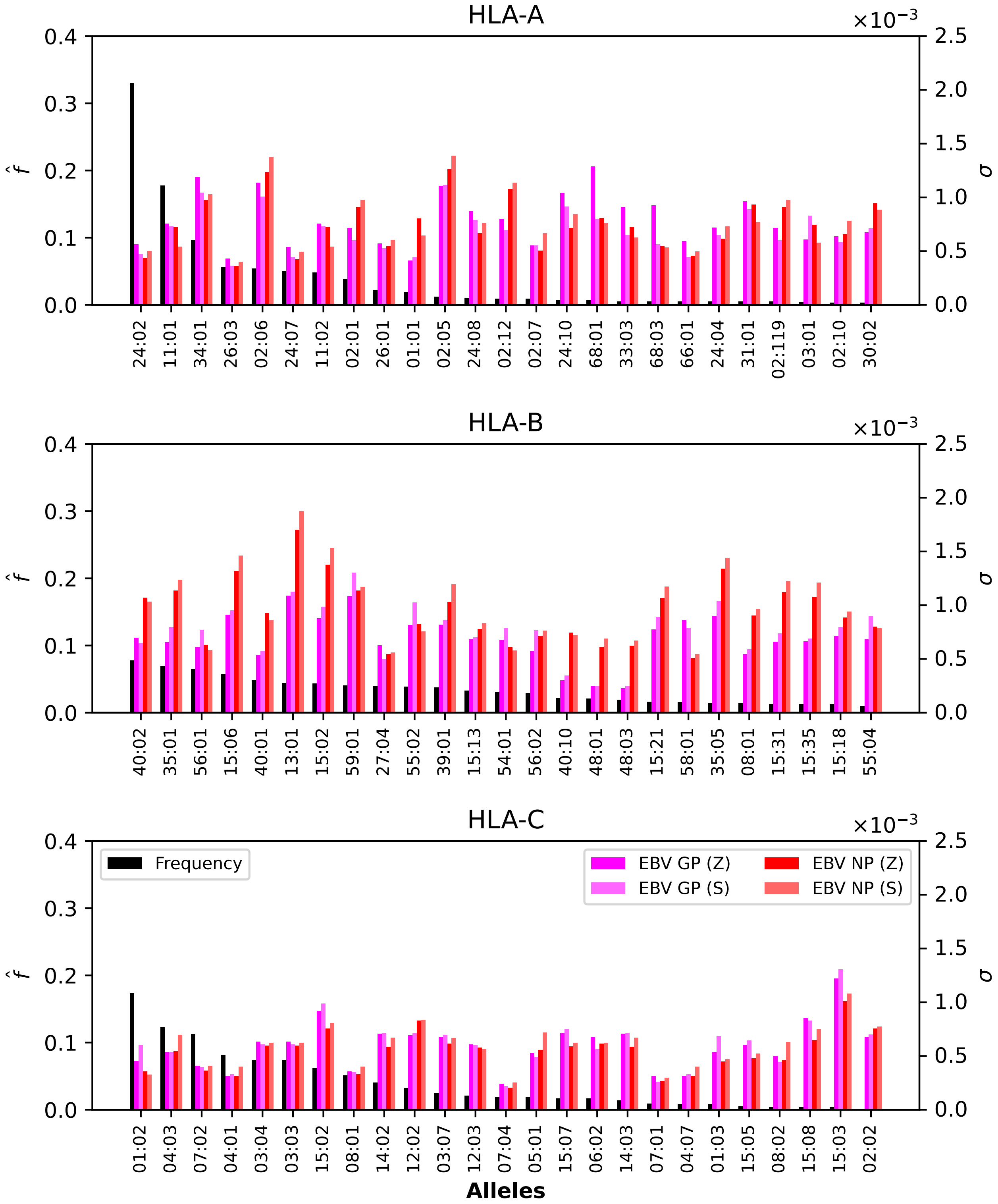

Figure S26. Normalized regional frequencies ($\hat{f}_i^{(6)}$) and Ebola σ_i values for the top 25 most frequent alleles of each type in Oceania. The top panel represents HLA-A alleles, the middle HLA-B, and the bottom HLA-C. From left to right, the bars in each group represent frequency, Ebola GP1 (Zaire), Ebola GP1 (Sudan), Ebola NP (Zaire), and Ebola NP (Sudan).

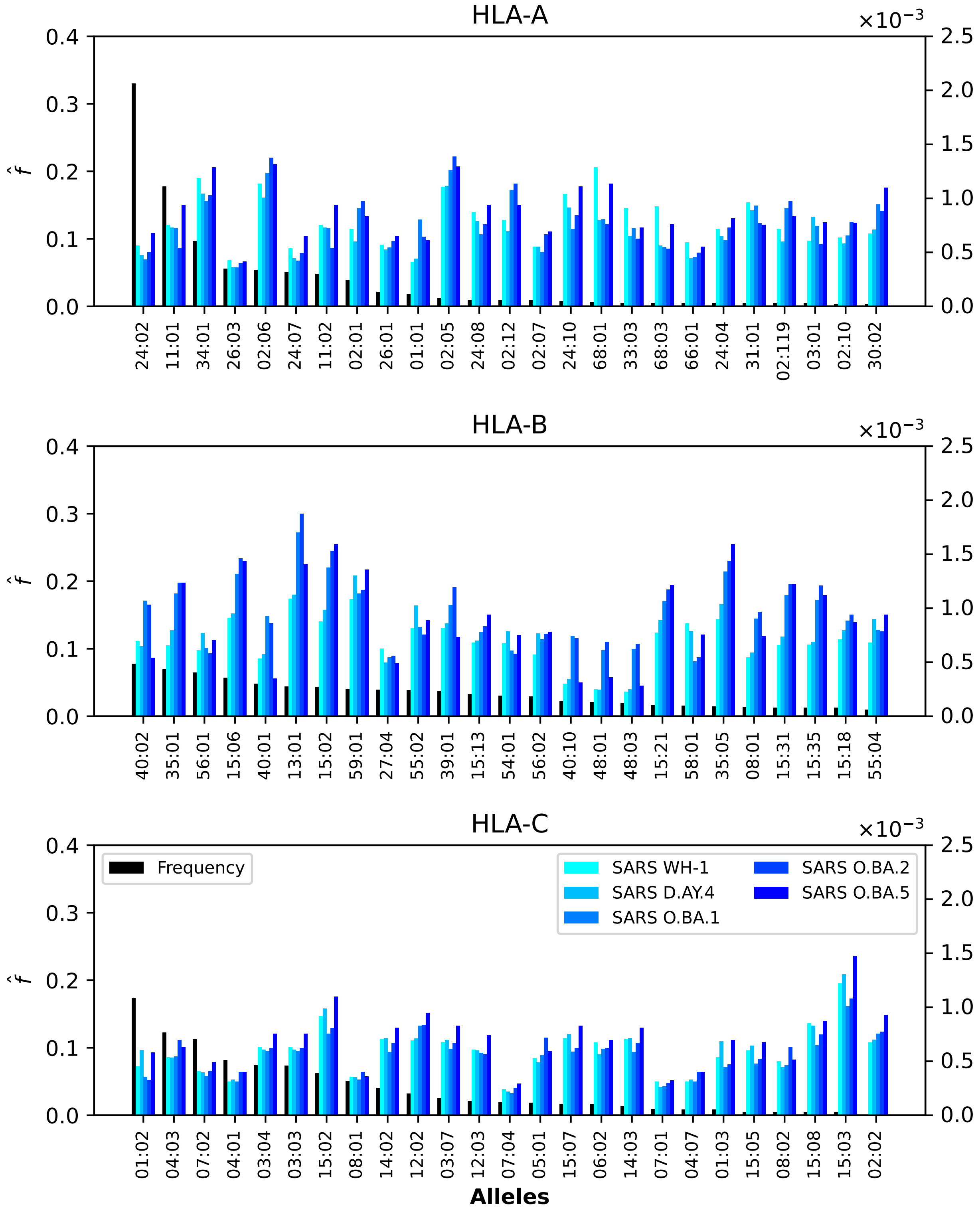

Figure S27. Normalized regional frequencies ($\hat{f}_i^{(6)}$) and SARS-CoV-2 σ_i values for the top 25 most frequent alleles of each type in Oceania. The top panel represents HLA-A alleles, the middle HLA-B, and the bottom HLA-C. From left to right, the bars in each group represent frequency, SARS-CoV-2 Wuhan-Hu-1, SARS-CoV-2 Delta AY.4, SARS-CoV-2 Omicron BA.1, SARS-CoV-2 Omicron BA.2, SARS-CoV-2 Omicron BA.5.

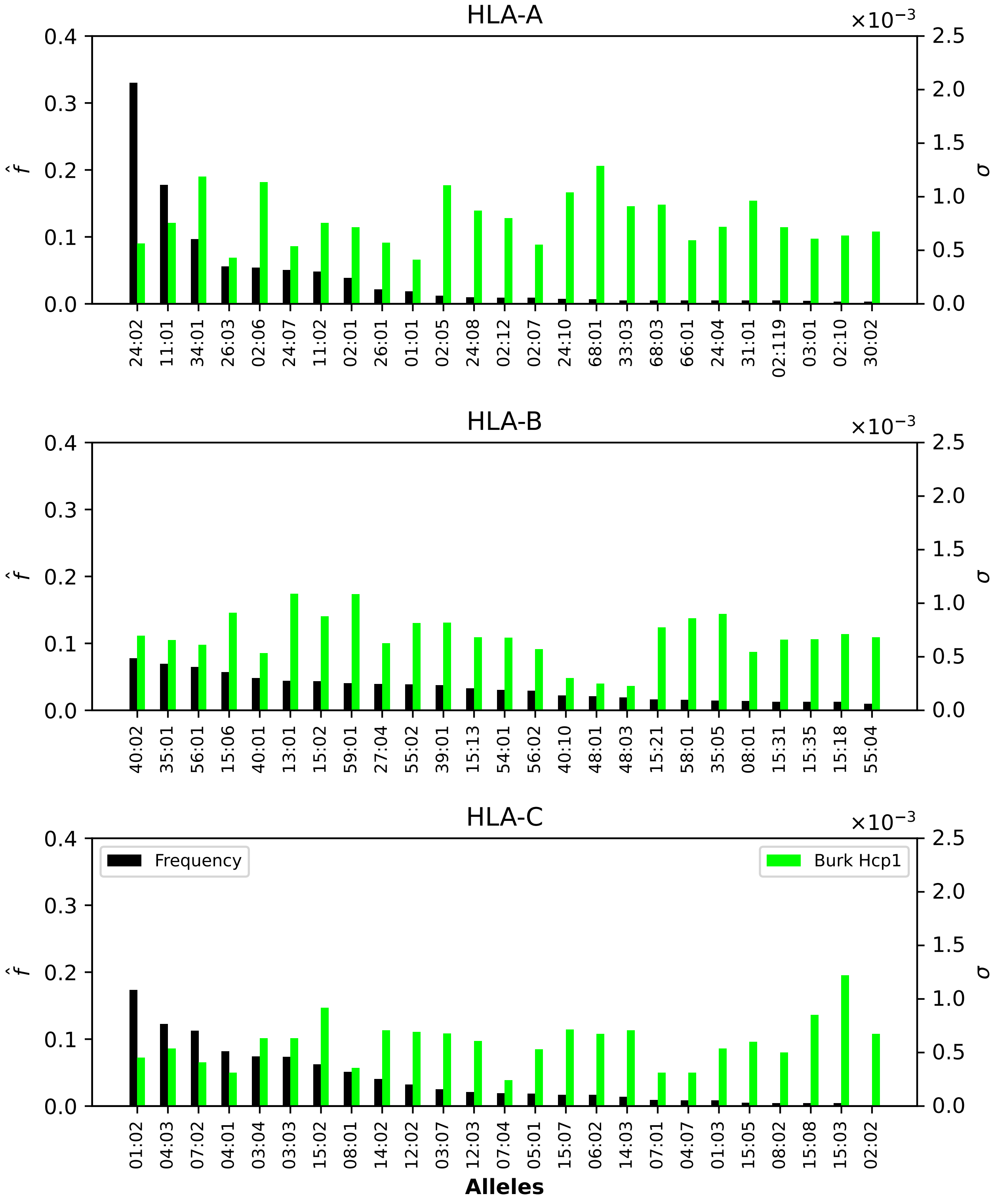

Figure S28. Normalized regional frequencies ($\hat{f}_i^{(6)}$) and Burkholderia σ_i values for the top 25 most frequent alleles of each type in Oceania. The top panel represents HLA-A alleles, the middle HLA-B, and the bottom HLA-C. From left to right, the bars in each group represent frequency and Burkholderia Hcp1.

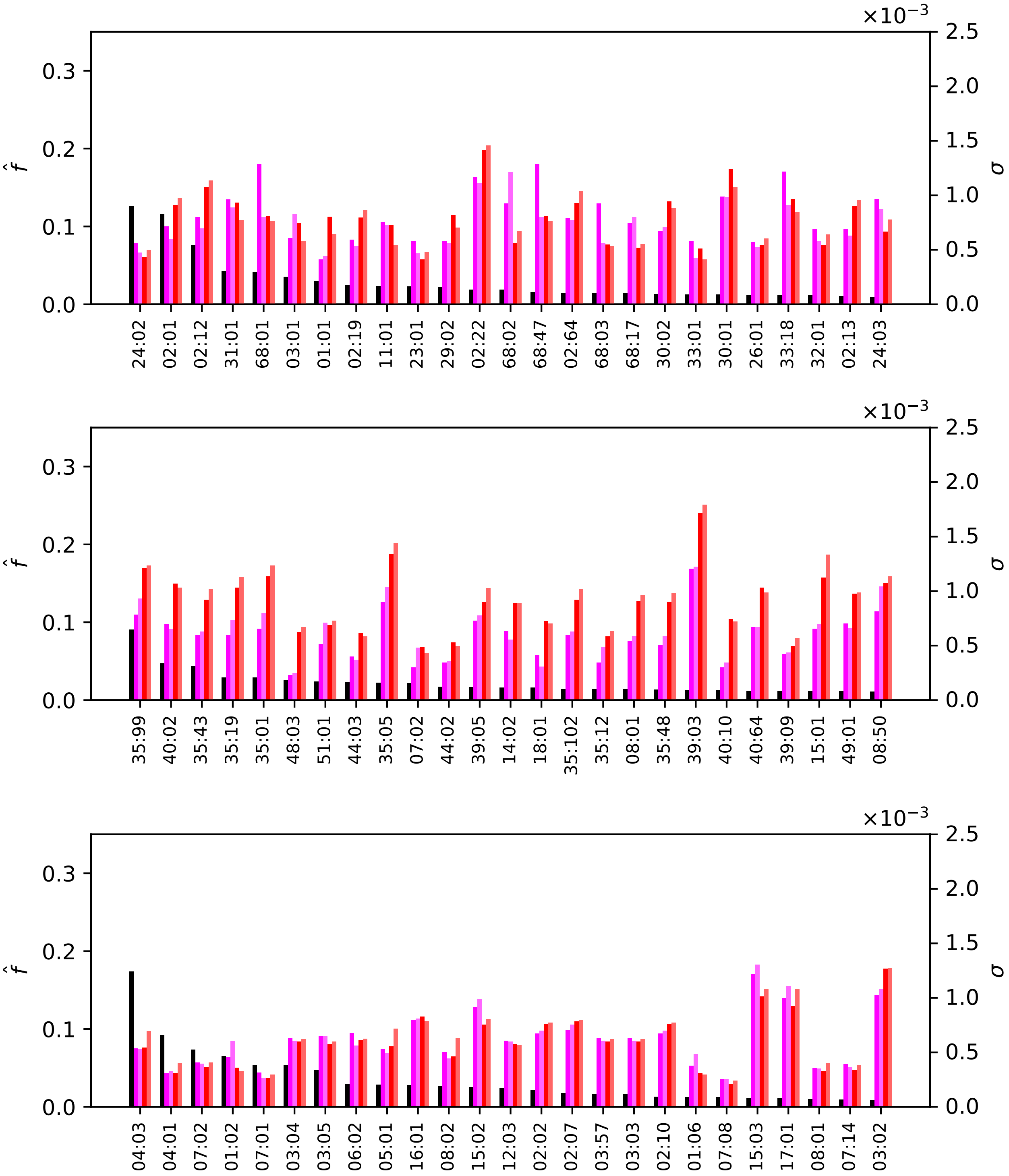

Figure S29. Normalized regional frequencies ($\hat{f}_i^{(7)}$) and Ebola σ_i values for the top 25 most frequent alleles of each type in South and Central America. The top panel represents HLA-A alleles, the middle HLA-B, and the bottom HLA-C. From left to right, the bars in each group represent frequency, Ebola GP1 (Zaire), Ebola GP1 (Sudan), Ebola NP (Zaire), and Ebola NP (Sudan).

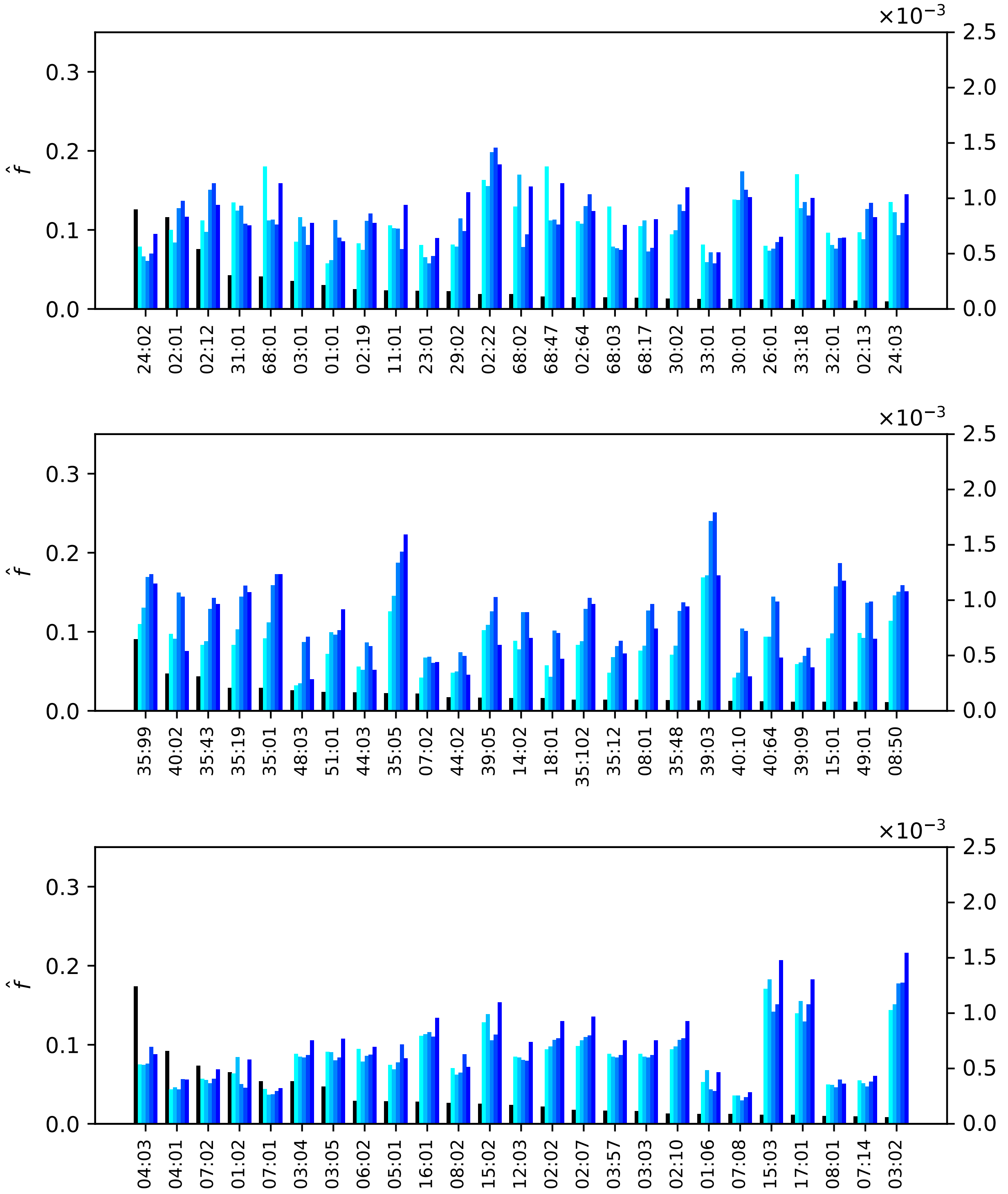

Figure S30. Normalized regional frequencies ($\hat{f}_i^{(7)}$) and SARS-CoV-2 σ_i values for the top 25 most frequent alleles of each type in South and Central America. The top panel represents HLA-A alleles, the middle HLA-B, and the bottom HLA-C. From left to right, the bars in each group represent frequency, SARS-CoV-2 Wuhan-Hu-1, SARS-CoV-2 Delta AY.4, SARS-CoV-2 Omicron BA.1, SARS-CoV-2 Omicron BA.2, SARS-CoV-2 Omicron BA.5.

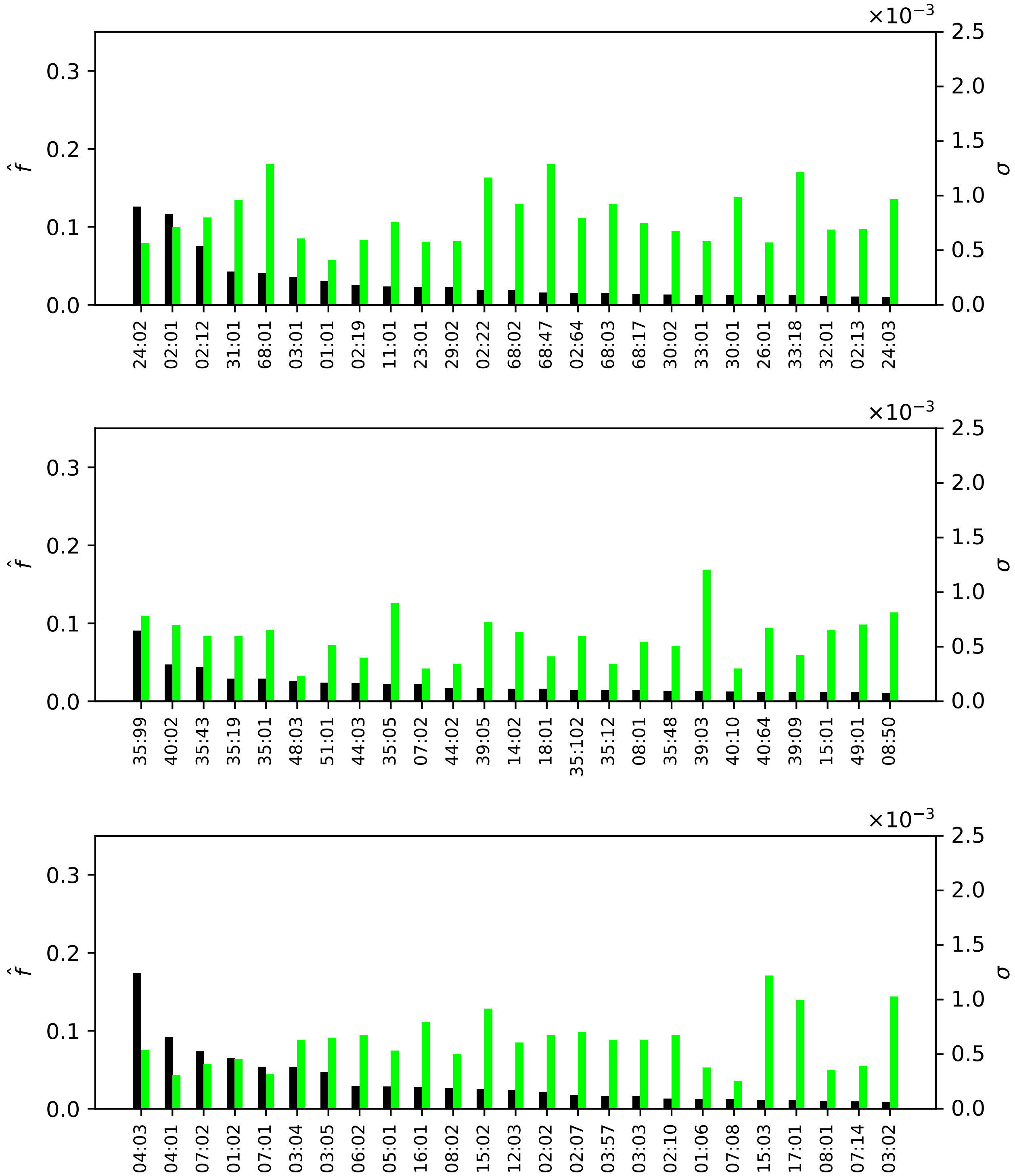

Figure S31. Normalized regional frequencies ($\hat{f}_i^{(7)}$) and Burkholderia σ_i values for the top 25 most frequent alleles of each type in South and Central America. The top panel represents HLA-A alleles, the middle HLA-B, and the bottom HLA-C. From left to right, the bars in each group represent frequency and Burkholderia Hcp1.

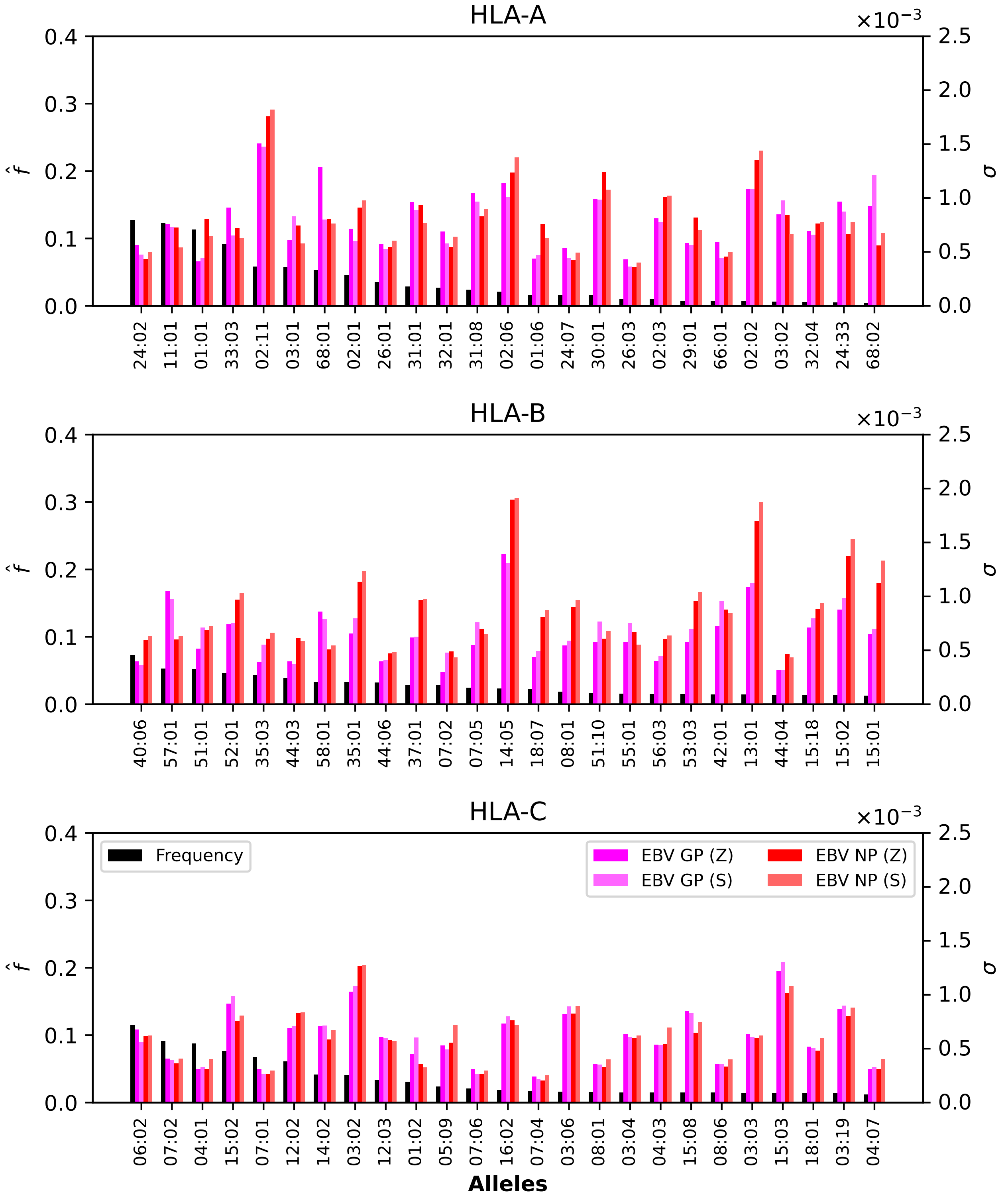

Figure S32. Normalized regional frequencies ($\hat{f}_i^{(8)}$) and Ebola σ_i values for the top 25 most frequent alleles of each type in South Asia. The top panel represents HLA-A alleles, the middle HLA-B, and the bottom HLA-C. From left to right, the bars in each group represent frequency, Ebola GP1 (Zaire), Ebola GP1 (Sudan), Ebola NP (Zaire), and Ebola NP (Sudan).

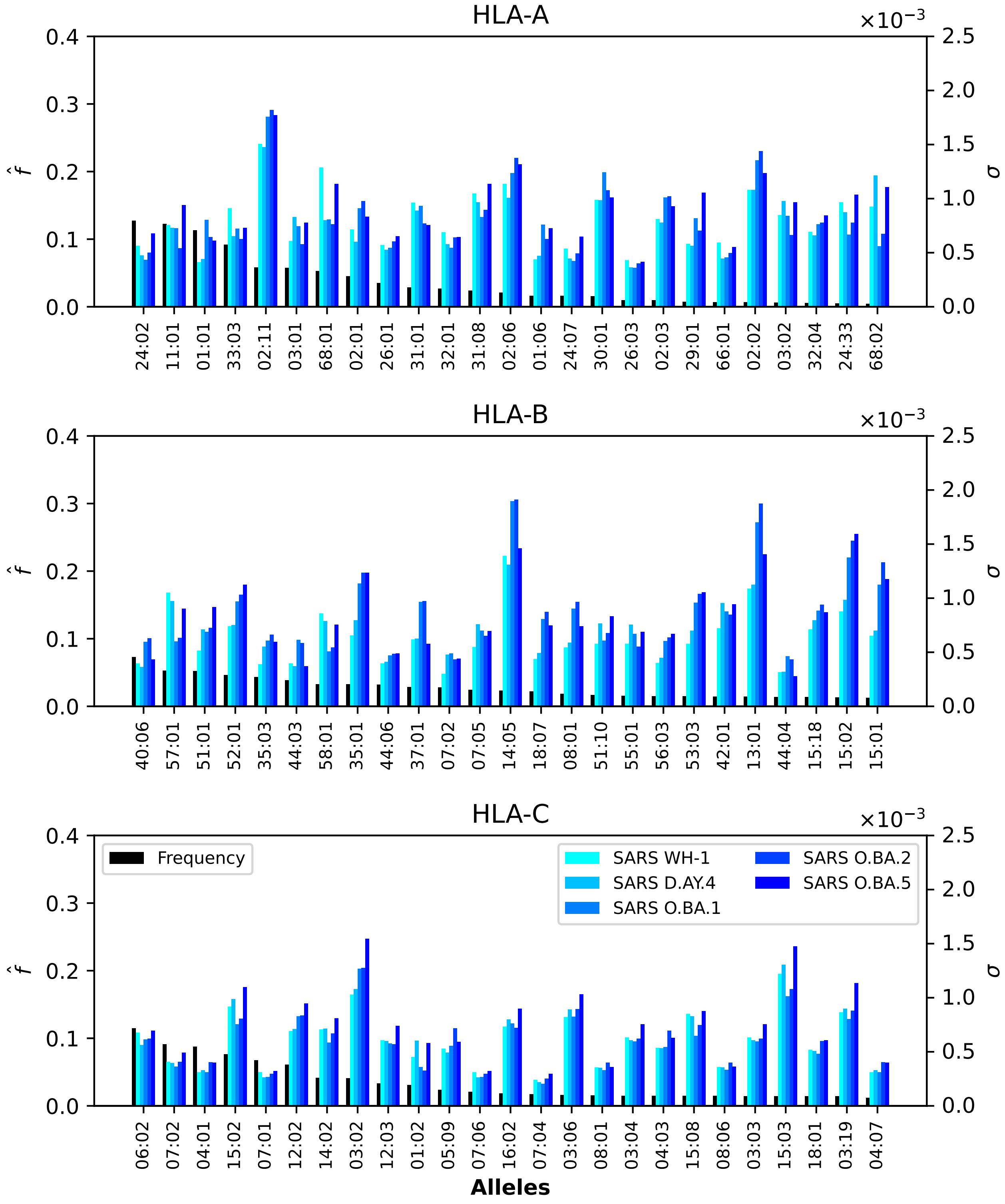

Figure S33. Normalized regional frequencies ($\hat{f}_i^{(8)}$) and SARS-CoV-2 σ_i values for the top 25 most frequent alleles of each type in South Asia. The top panel represents HLA-A alleles, the middle HLA-B, and the bottom HLA-C. From left to right, the bars in each group represent frequency, SARS-CoV-2 Wuhan-Hu-1, SARS-CoV-2 Delta AY.4, SARS-CoV-2 Omicron BA.1, SARS-CoV-2 Omicron BA.2, SARS-CoV-2 Omicron BA.5.

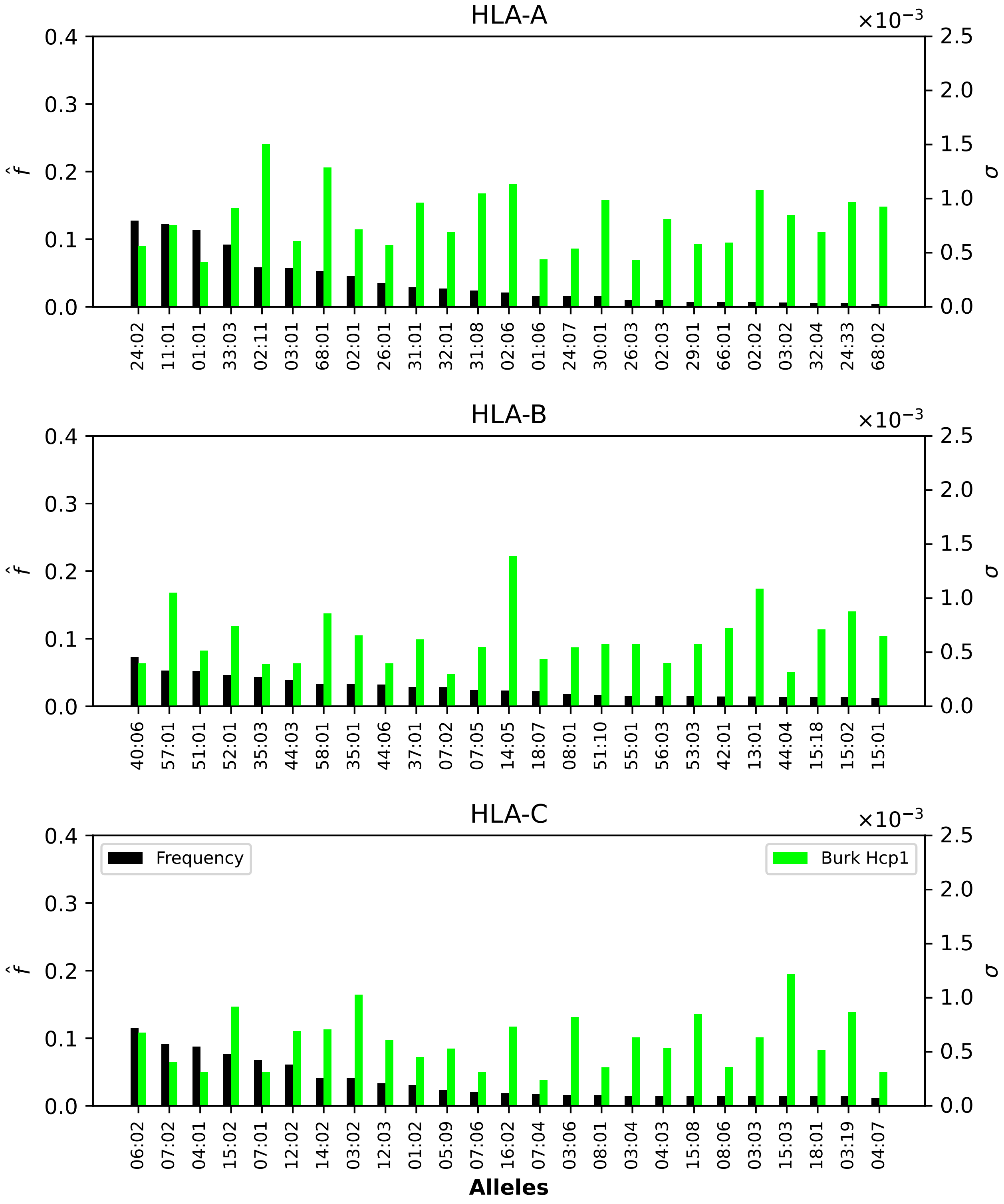

Figure S34. Normalized regional frequencies ($\hat{f}_i^{(8)}$) and Burkholderia σ_i values for the top 25 most frequent alleles of each type in South Asia. The top panel represents HLA-A alleles, the middle HLA-B, and the bottom HLA-C. From left to right, the bars in each group represent frequency and Burkholderia Hcp1.

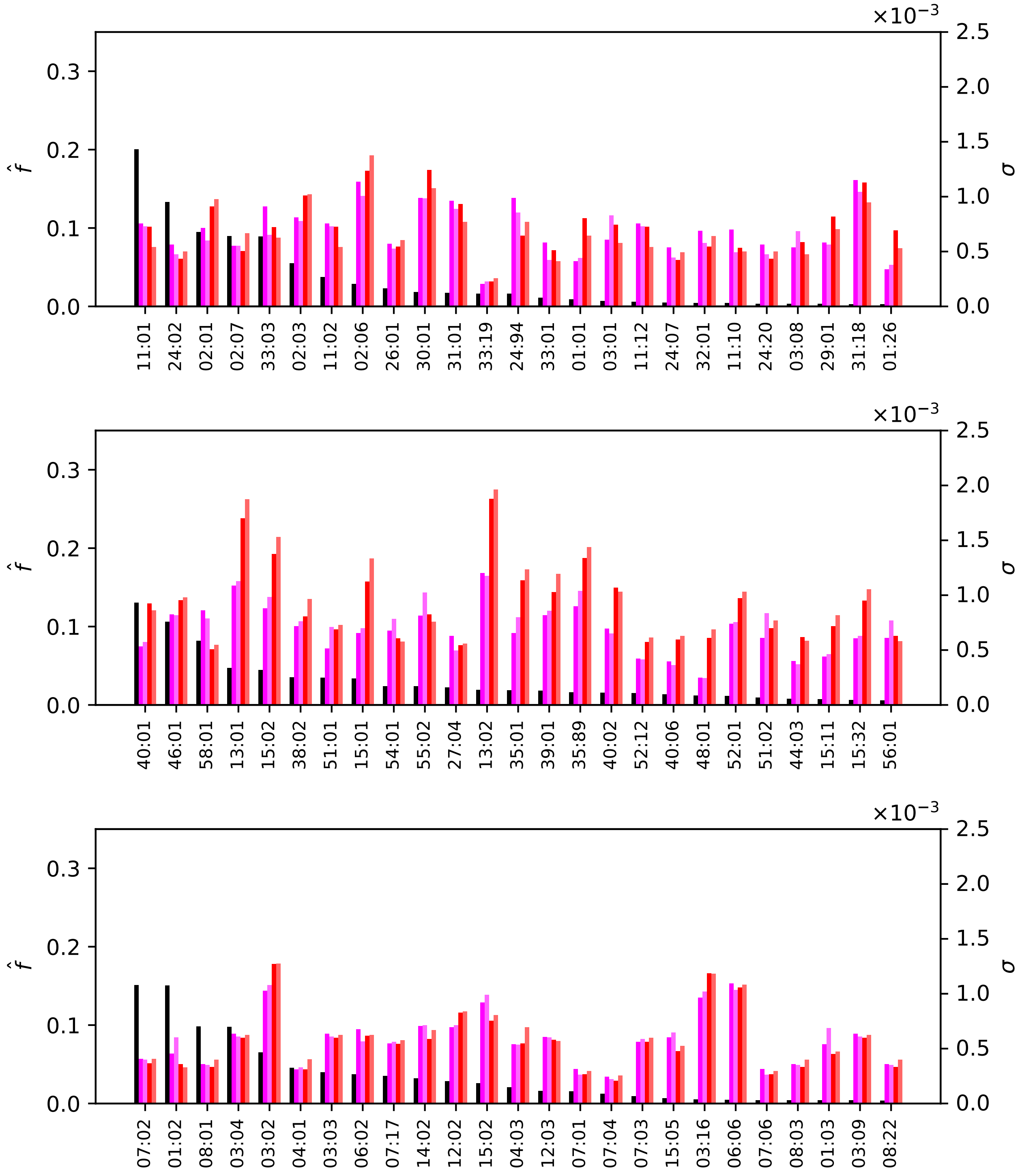

Figure S35. Normalized regional frequencies ($\hat{f}_i^{(9)}$) and Ebola σ_i values for the top 25 most frequent alleles of each type in Southeast Asia. The top panel represents HLA-A alleles, the middle HLA-B, and the bottom HLA-C. From left to right, the bars in each group represent frequency, Ebola GP1 (Zaire), Ebola GP1 (Sudan), Ebola NP (Zaire), and Ebola NP (Sudan).

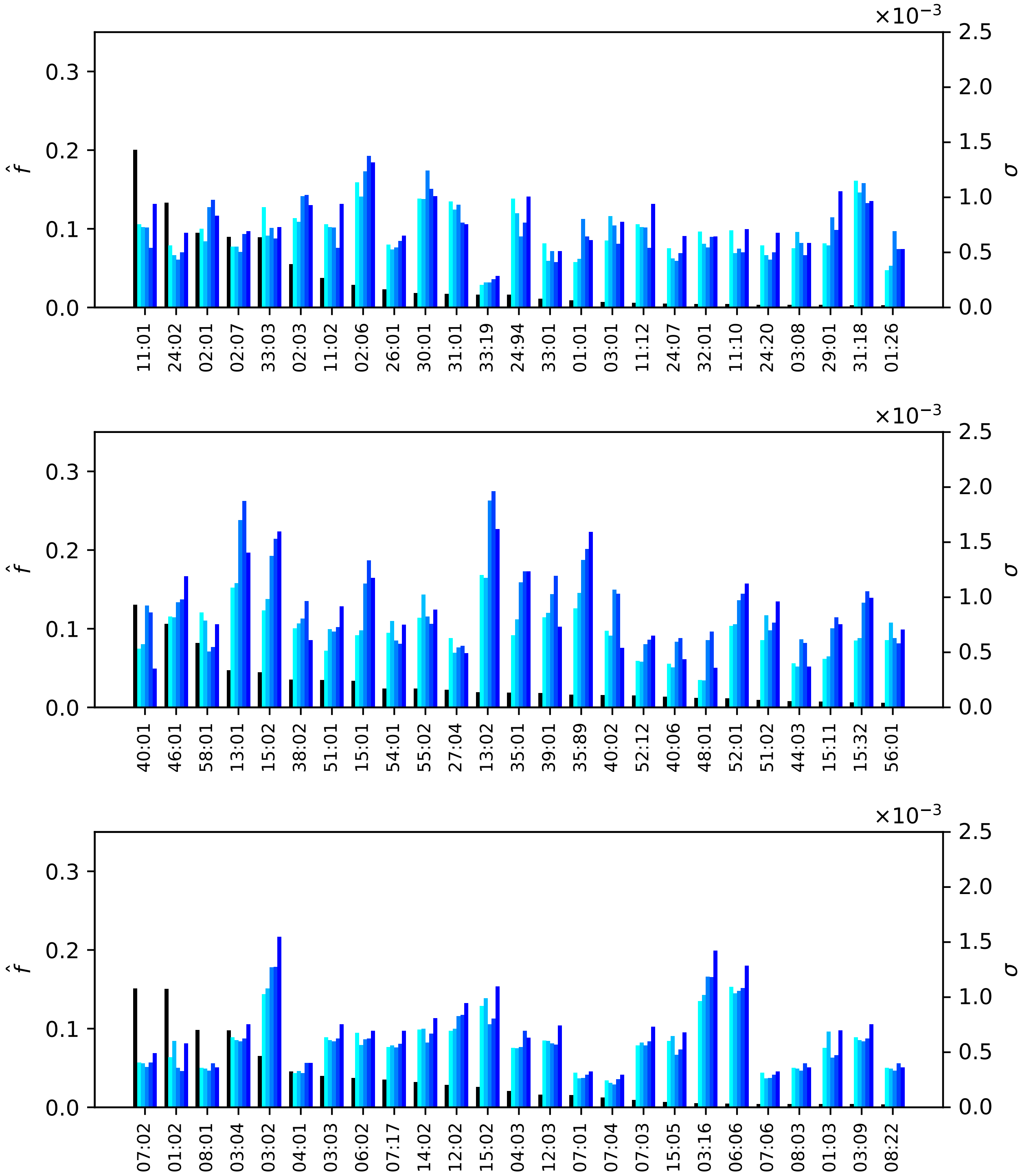

Figure S36. Normalized regional frequencies ($\hat{f}_i^{(9)}$) and SARS-CoV-2 σ_i values for the top 25 most frequent alleles of each type in Southeast Asia. The top panel represents HLA-A alleles, the middle HLA-B, and the bottom HLA-C. From left to right, the bars in each group represent frequency, SARS-CoV-2 Wuhan-Hu-1, SARS-CoV-2 Delta AY.4, SARS-CoV-2 Omicron BA.1, SARS-CoV-2 Omicron BA.2, SARS-CoV-2 Omicron BA.5.

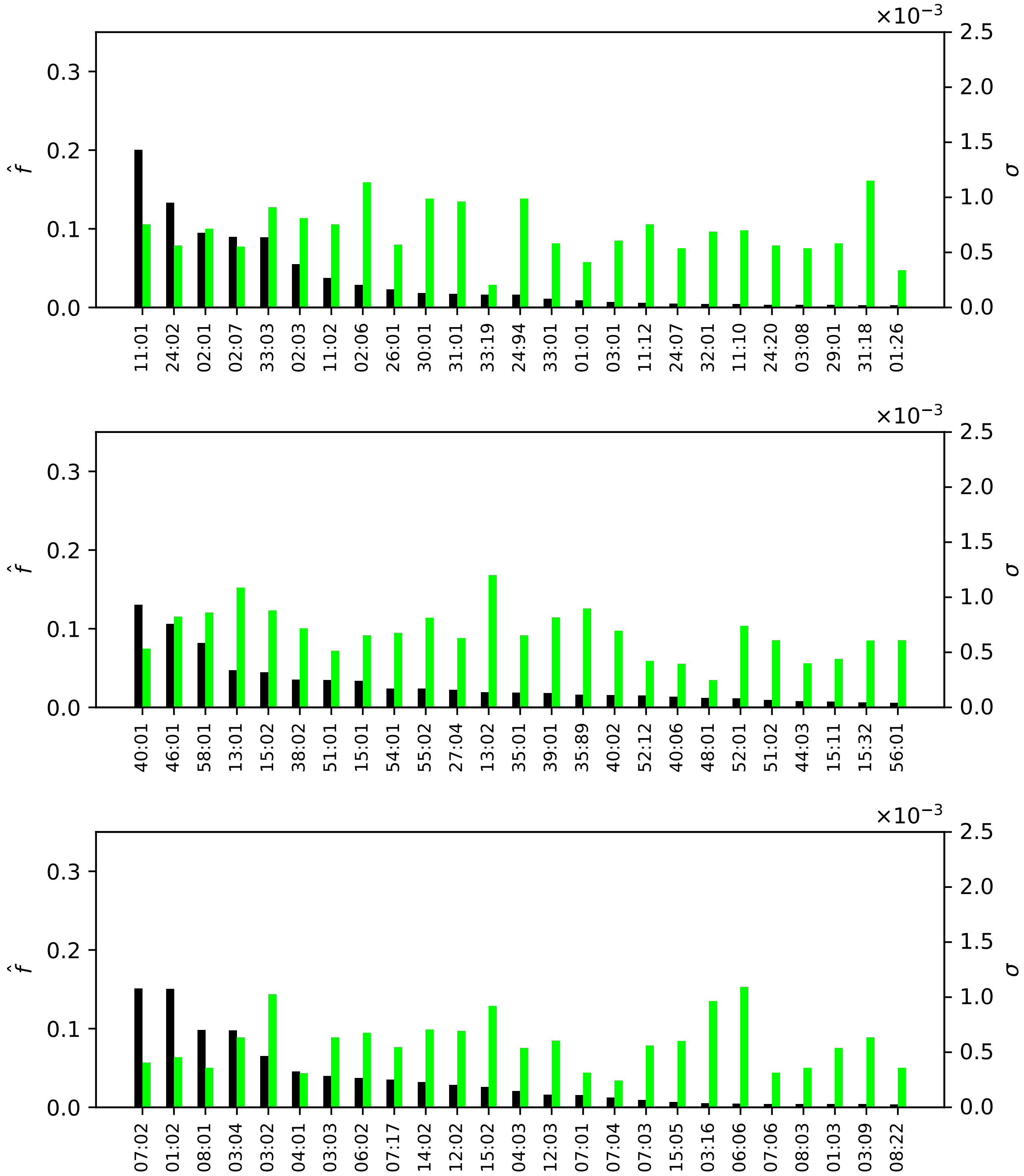

Figure S37. Normalized regional frequencies ($\hat{f}_i^{(9)}$) and Burkholderia σ_i values for the top 25 most frequent alleles of each type in Southeast Asia. The top panel represents HLA-A alleles, the middle HLA-B, and the bottom HLA-C. From left to right, the bars in each group represent frequency and Burkholderia Hcp1.

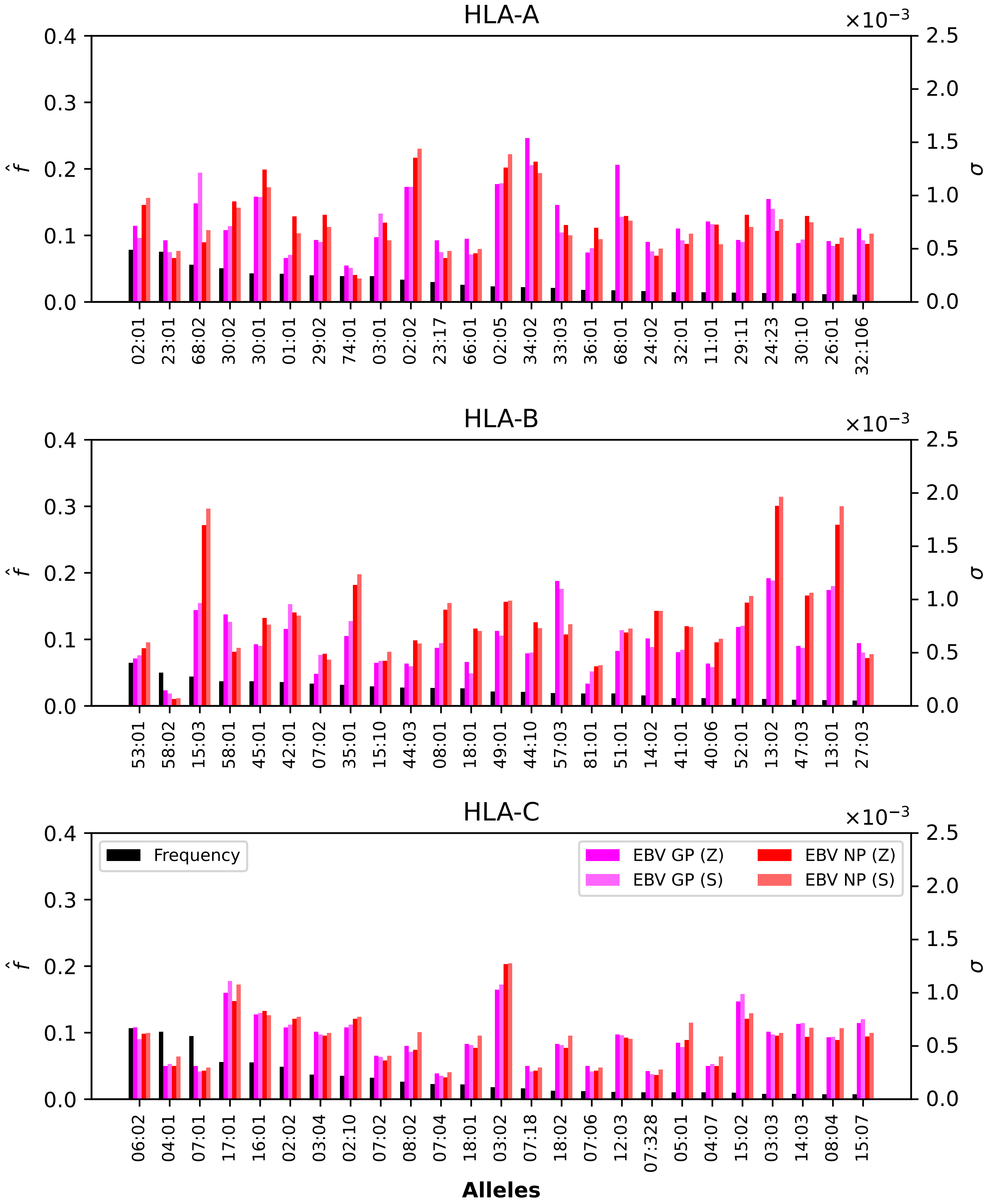

Figure S38. Normalized regional frequencies ($\hat{f}_i^{(10)}$) and Ebola σ_i values for the top 25 most frequent alleles of each type in Sub-Saharan Africa. The top panel represents HLA-A alleles, the middle HLA-B, and the bottom HLA-C. From left to right, the bars in each group represent frequency, Ebola GP1 (Zaire), Ebola GP1 (Sudan), Ebola NP (Zaire), and Ebola NP (Sudan).

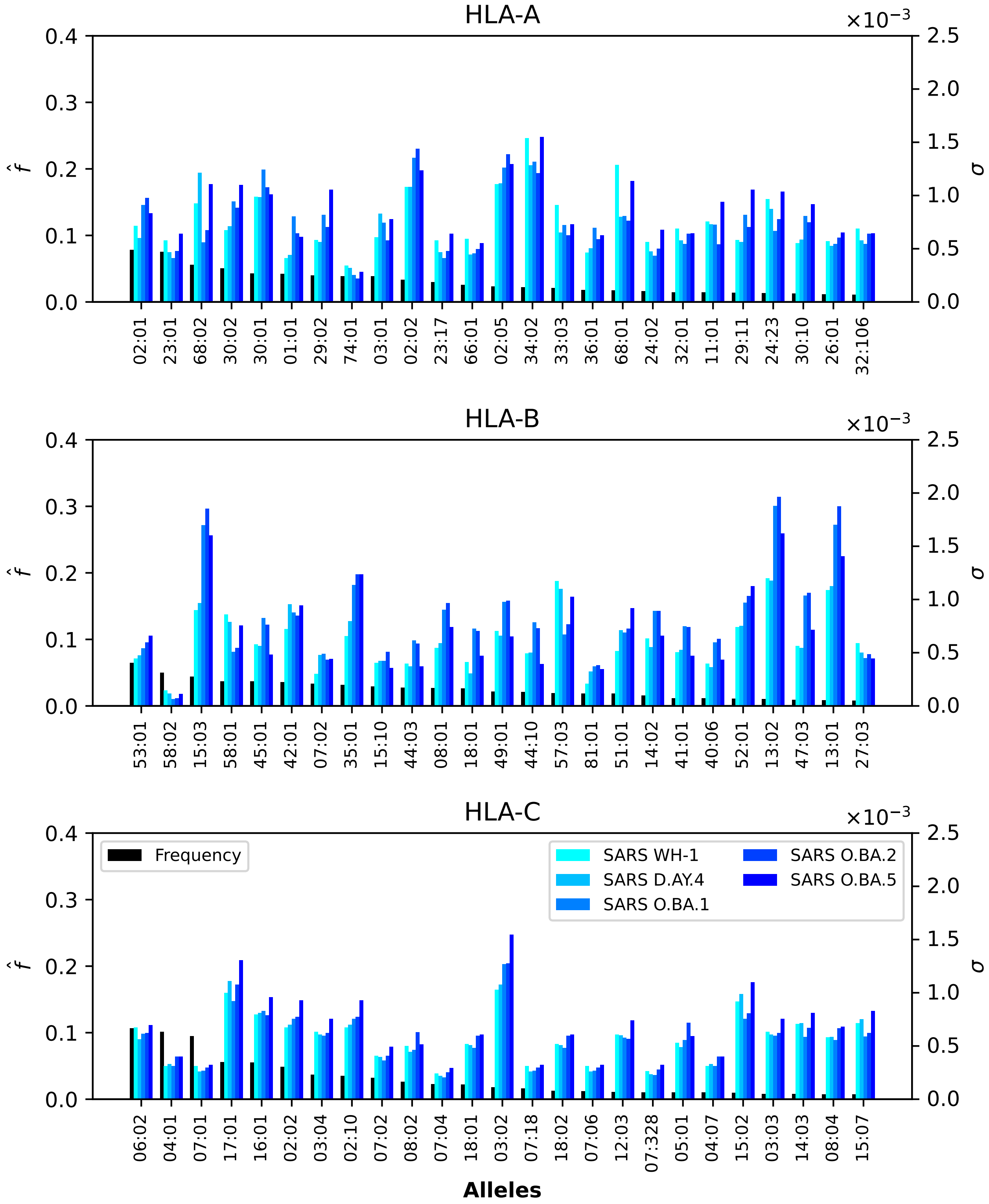

Figure S39. Normalized regional frequencies ($\hat{f}_i^{(10)}$) and SARS-CoV-2 σ_i values for the top 25 most frequent alleles of each type in Sub-Saharan Africa. The top panel represents HLA-A alleles, the middle HLA-B, and the bottom HLA-C. From left to right, the bars in each group represent frequency, SARS-CoV-2 Wuhan-Hu-1, SARS-CoV-2 Delta AY.4, SARS-CoV-2 Omicron BA.1, SARS-CoV-2 Omicron BA.2, SARS-CoV-2 Omicron BA.5.

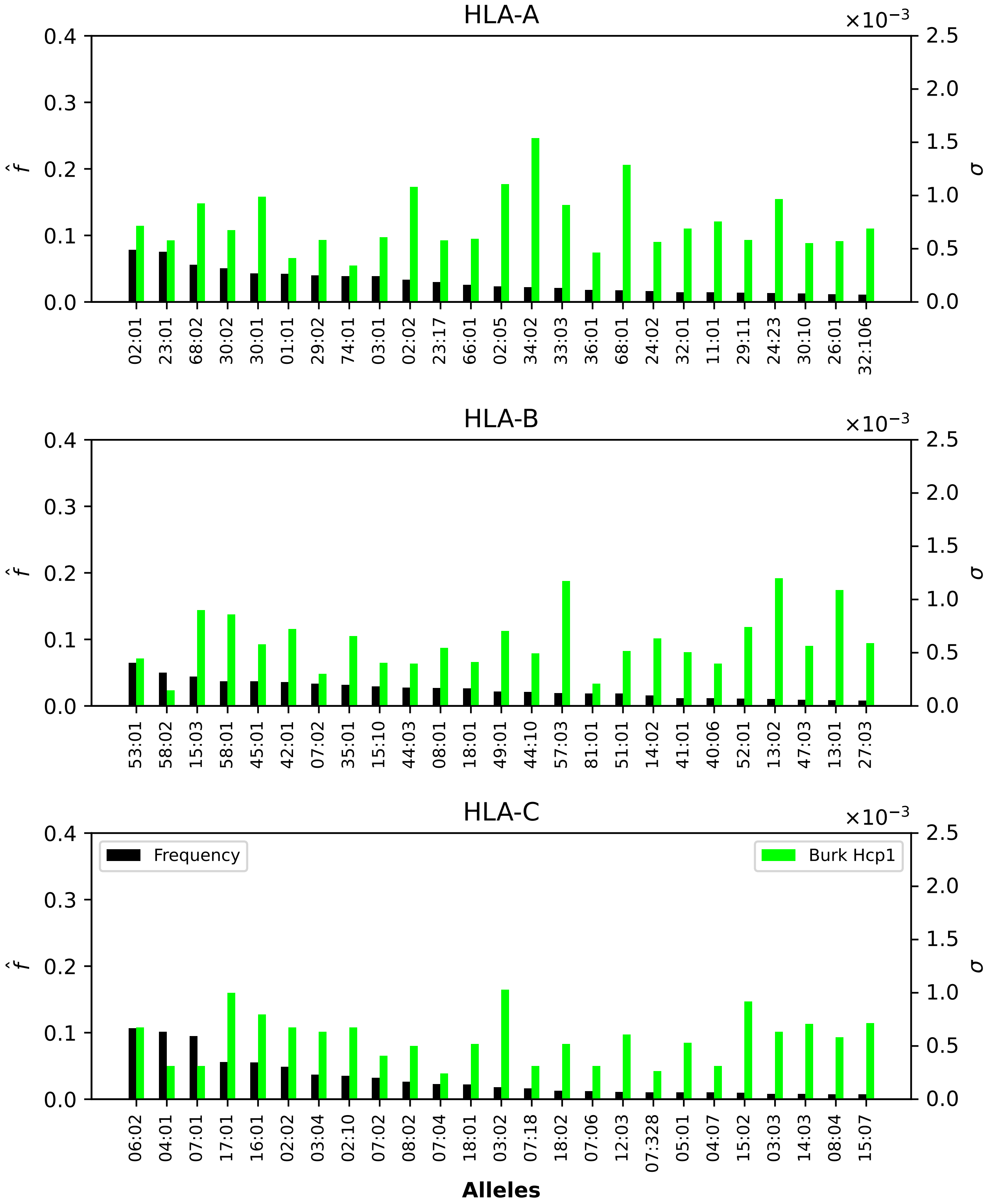

Figure S40. Normalized regional frequencies ($\hat{f}_i^{(10)}$) and Burkholderia σ_i values for the top 25 most frequent alleles of each type in Sub-Saharan Africa. The top panel represents HLA-A alleles, the middle HLA-B, and the bottom HLA-C. From left to right, the bars in each group represent frequency and Burkholderia Hcp1.

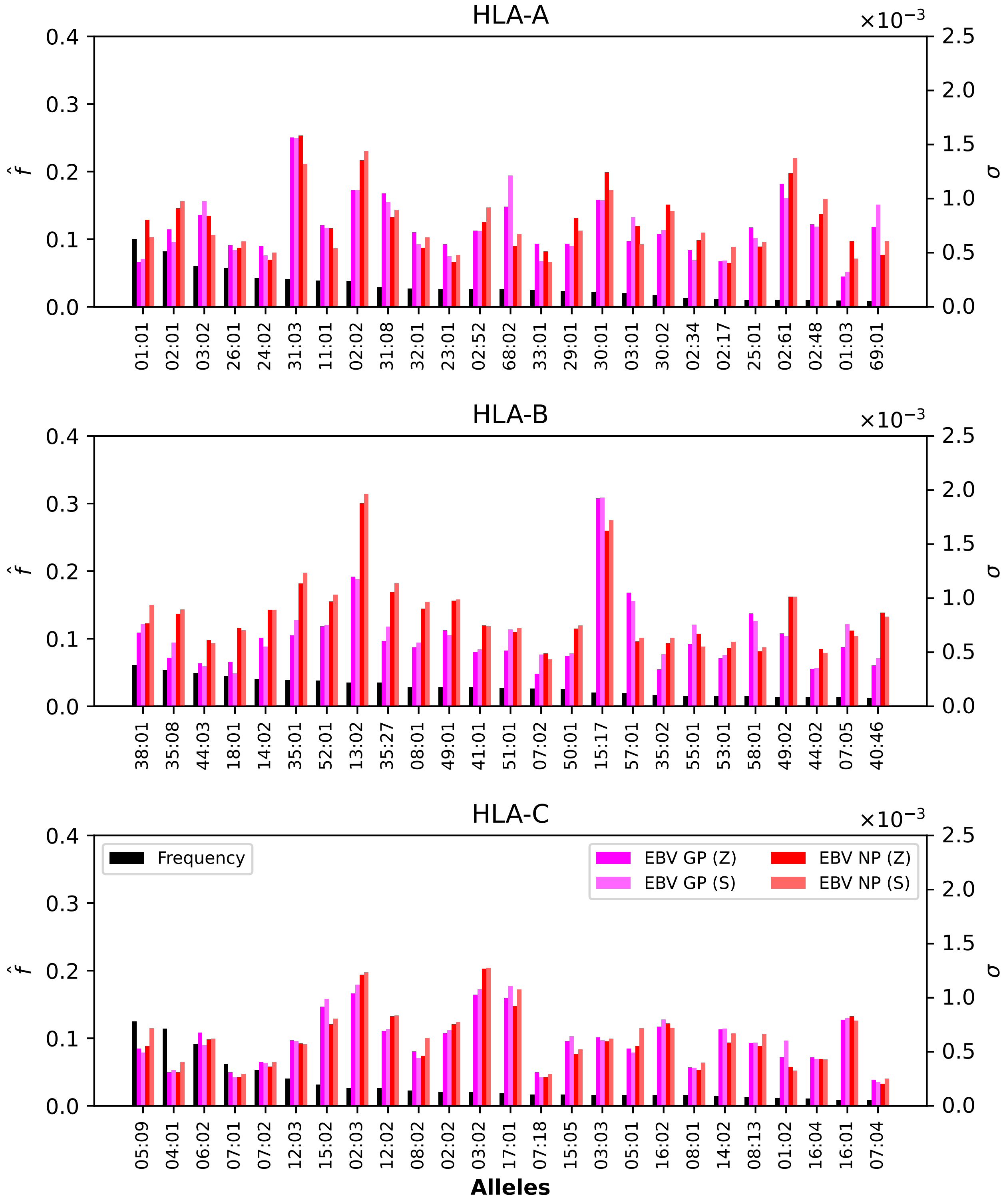

Figure S41. Normalized regional frequencies ($\hat{f}_i^{(11)}$) and Ebola σ_i values for the top 25 most frequent alleles of each type in Western Asia. The top panel represents HLA-A alleles, the middle HLA-B, and the bottom HLA-C. From left to right, the bars in each group represent frequency, Ebola GP1 (Zaire), Ebola GP1 (Sudan), Ebola NP (Zaire), and Ebola NP (Sudan).

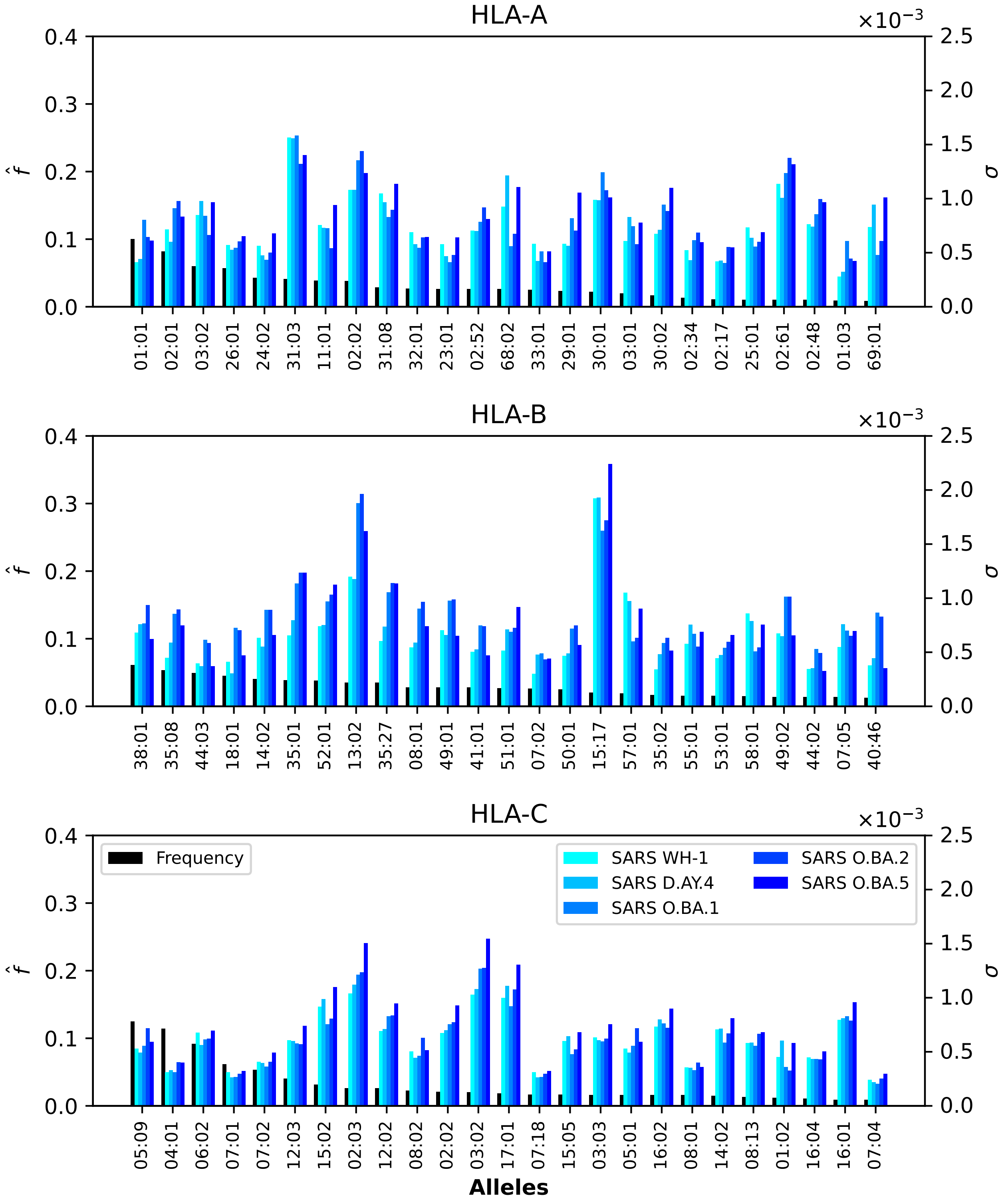

Figure S42. Normalized regional frequencies ($\hat{f}_i^{(11)}$) and SARS-CoV-2 σ_i values for the top 25 most frequent alleles of each type in Western Asia. The top panel represents HLA-A alleles, the middle HLA-B, and the bottom HLA-C. From left to right, the bars in each group represent frequency, SARS-CoV-2 Wuhan-Hu-1, SARS-CoV-2 Delta AY.4, SARS-CoV-2 Omicron BA.1, SARS-CoV-2 Omicron BA.2, SARS-CoV-2 Omicron BA.5.

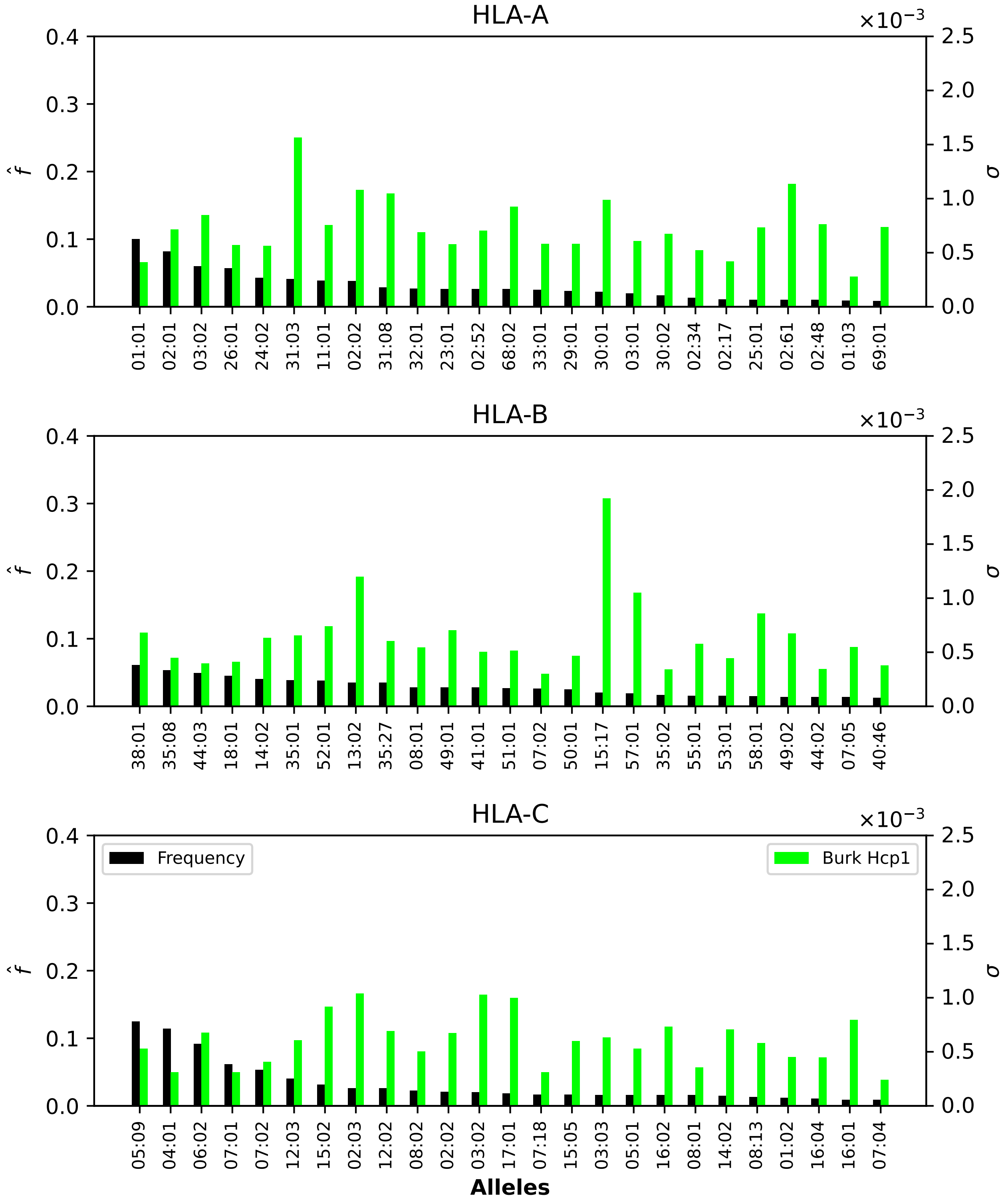

Figure S43. Normalized regional frequencies ($\hat{f}_i^{(11)}$) and Burkholderia σ_i values for the top 25 most frequent alleles of each type in Western Asia. The top panel represents HLA-A alleles, the middle HLA-B, and the bottom HLA-C. From left to right, the bars in each group represent frequency and Burkholderia Hcp1.

4 DISSECTING THE CONTRIBUTION TO THE INDIVIDUAL COVERAGE METRIC: ALLELE PAIR ANALYSIS FOR ALL REGIONS

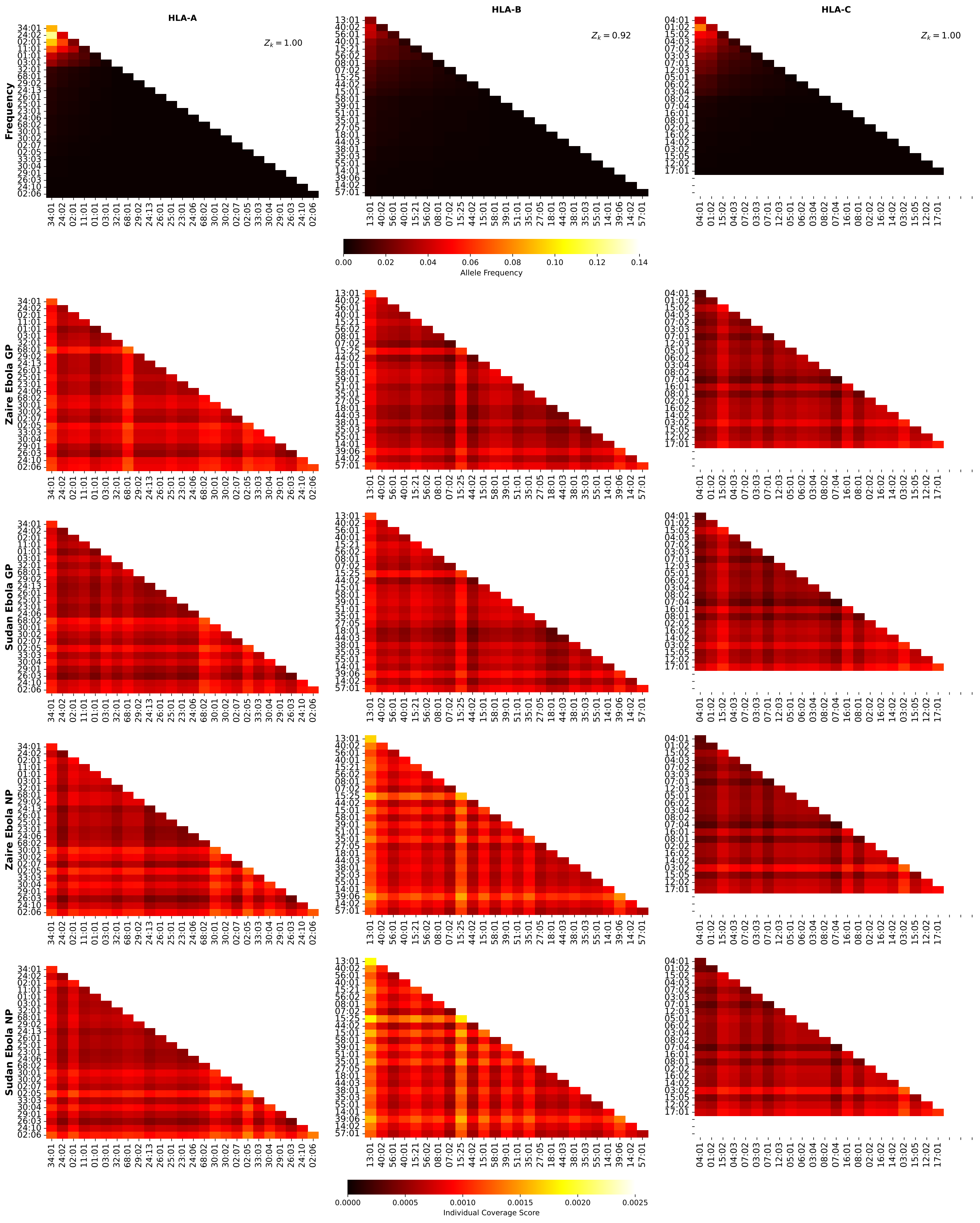

Figure S44. Frequencies and Ebola coverage scores for individuals in Australia. The 1st row corresponds to allele frequencies, the 2nd to GP1 Zaire, the 3rd to GP1 Sudan, the 4th to NP Zaire, and the 5th to NP Sudan. The 1st column is associated with HLA-A alleles, the 2nd to HLA-B, and the 3rd to HLA-C. The sum of the individual frequencies for each allele type is indicated on the panels in the 1st row.

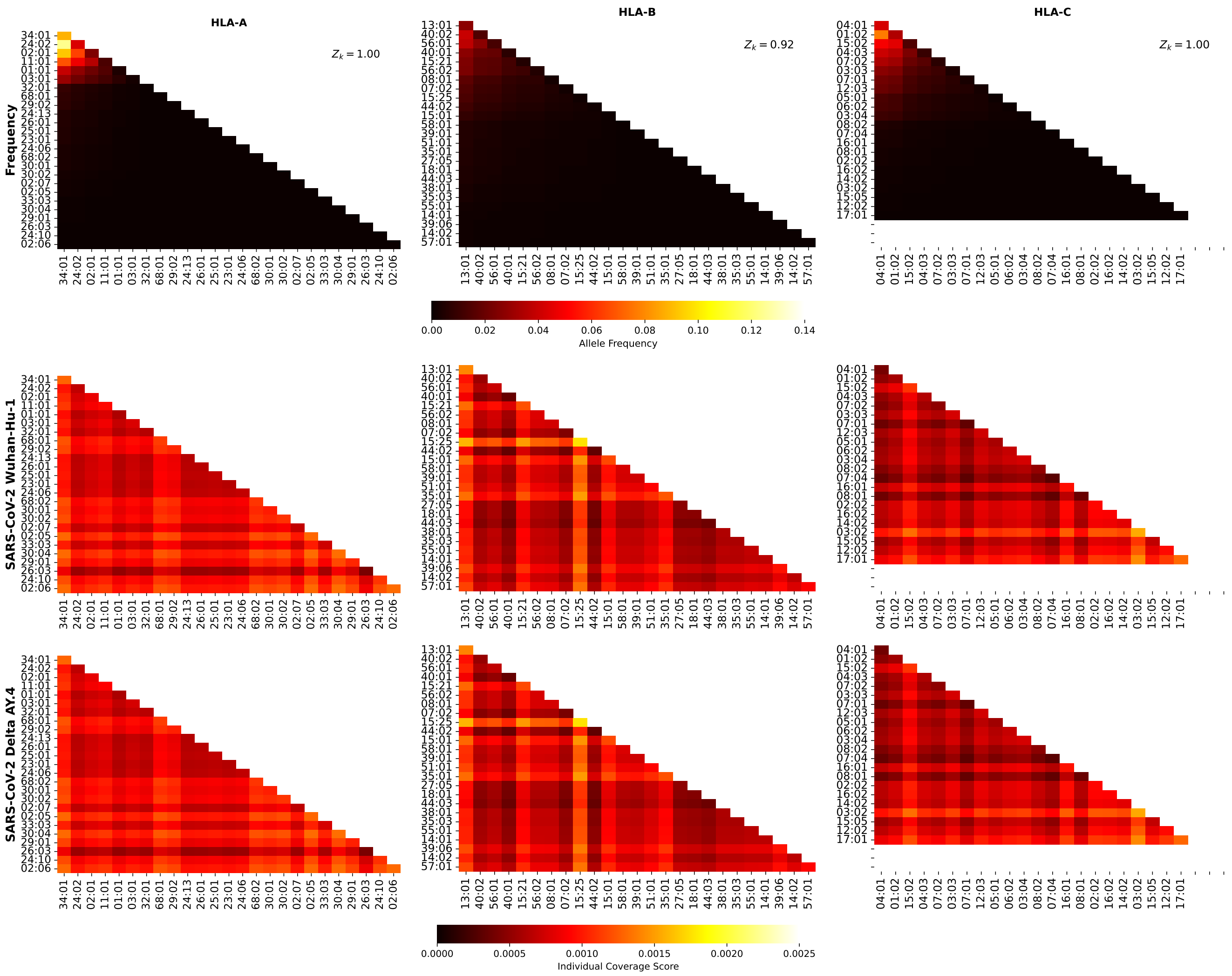

Figure S45. Frequencies and SARS-CoV-2 (Wuhan-Hu-1 and Delta AY.4 variants) coverage scores for individuals in Australia. The 1st row corresponds to allele frequencies, the 2nd to Wuhan-Hu-1, and the 3rd to Delta AY.4. The 1st column is associated with HLA-A alleles, the 2nd to HLA-B, and the 3rd to HLA-C. The sum of the individual frequencies for each allele type is indicated on the panels in the 1st row.

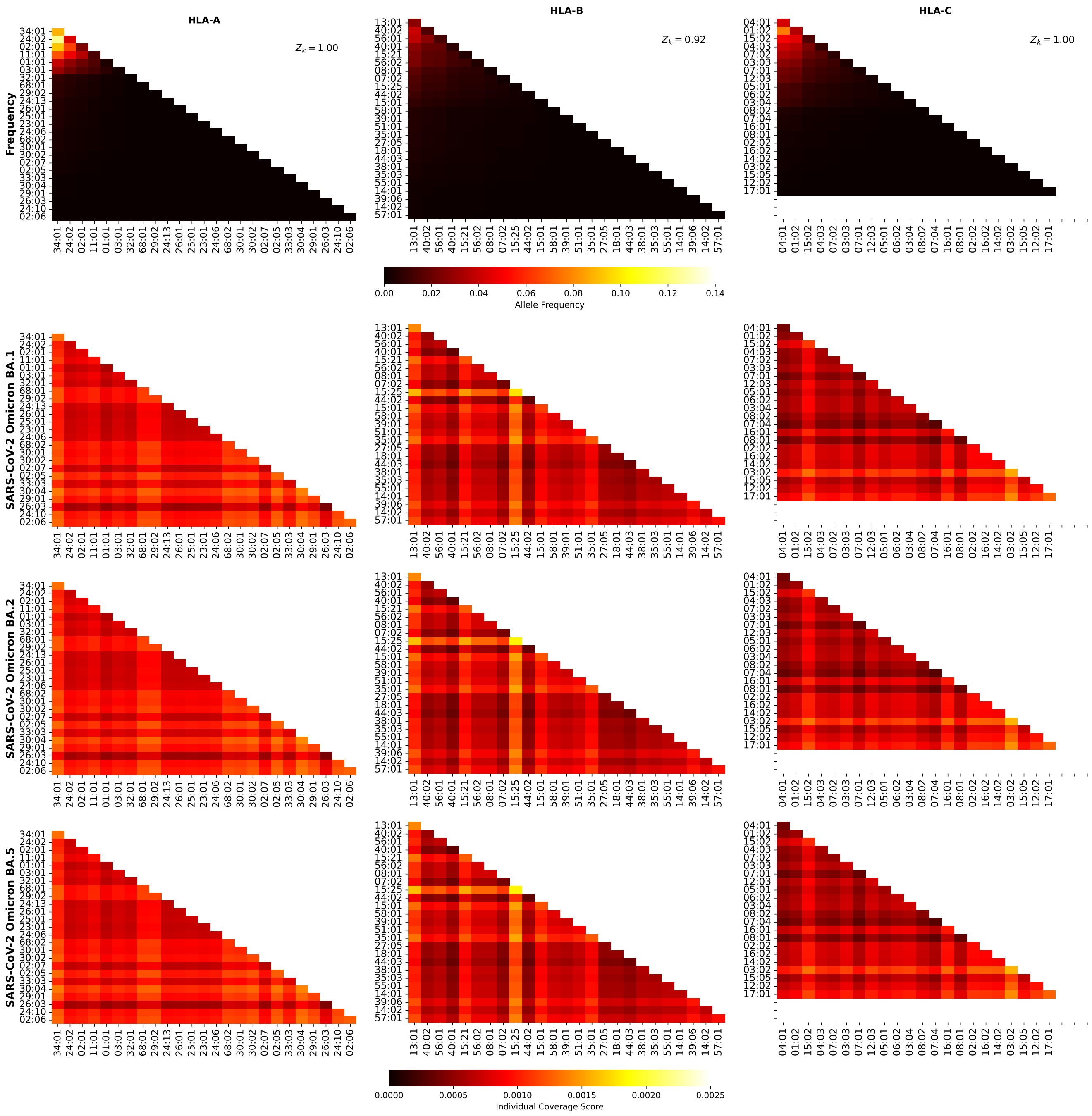

Figure S46. Frequencies and SARS-CoV-2 (Omicron variants) coverage scores for individuals in Australia. The 1st row corresponds to allele frequencies, the 2nd to BA.1, and the 3rd to BA.2, and the 4th to BA.5. The 1st column is associated with HLA-A alleles, the 2nd to HLA-B, and the 3rd to HLA-C. The sum of the individual frequencies for each allele type is indicated on the panels in the 1st row.

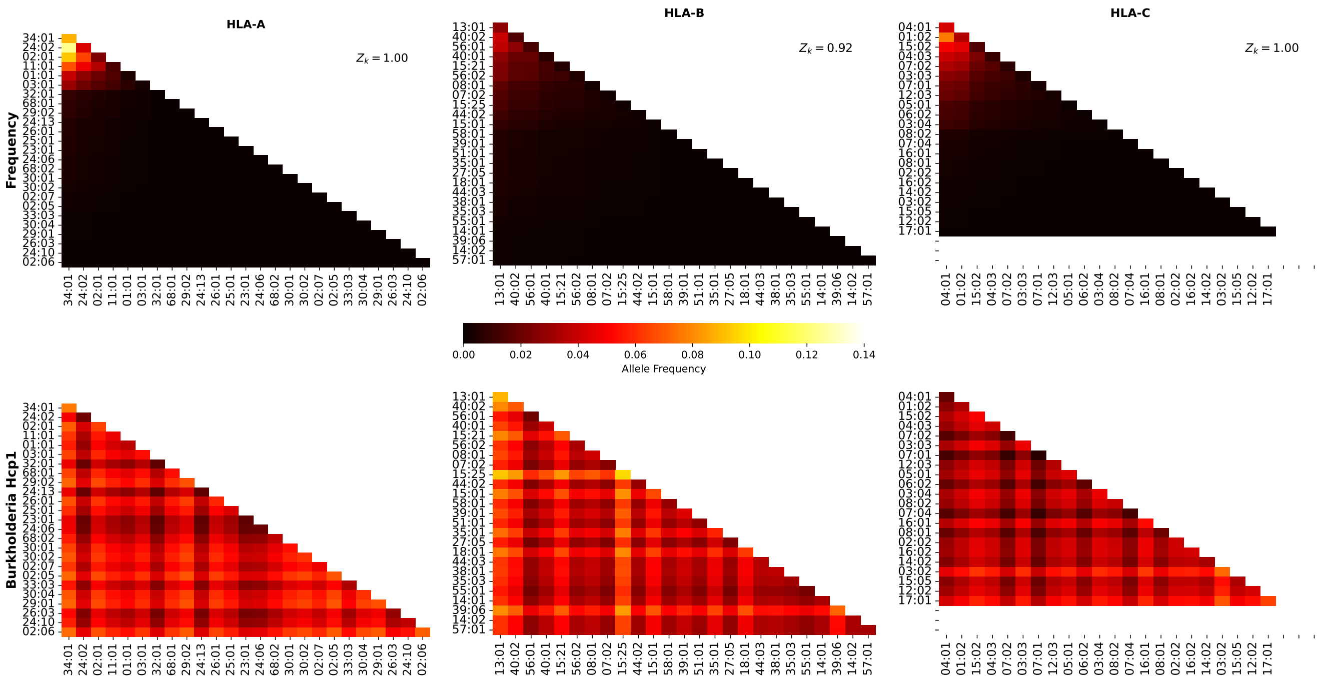

Figure S47. Frequencies and Burkholderia coverage scores for individuals in Australia. The 1st row corresponds to allele frequencies and the 2nd row to Burkholderia coverage score. The 1st column is associated with HLA-A alleles, the 2nd to HLA-B, and the 3rd to HLA-C. The sum of the individual frequencies for each allele type is indicated on the panels in the 1st row.

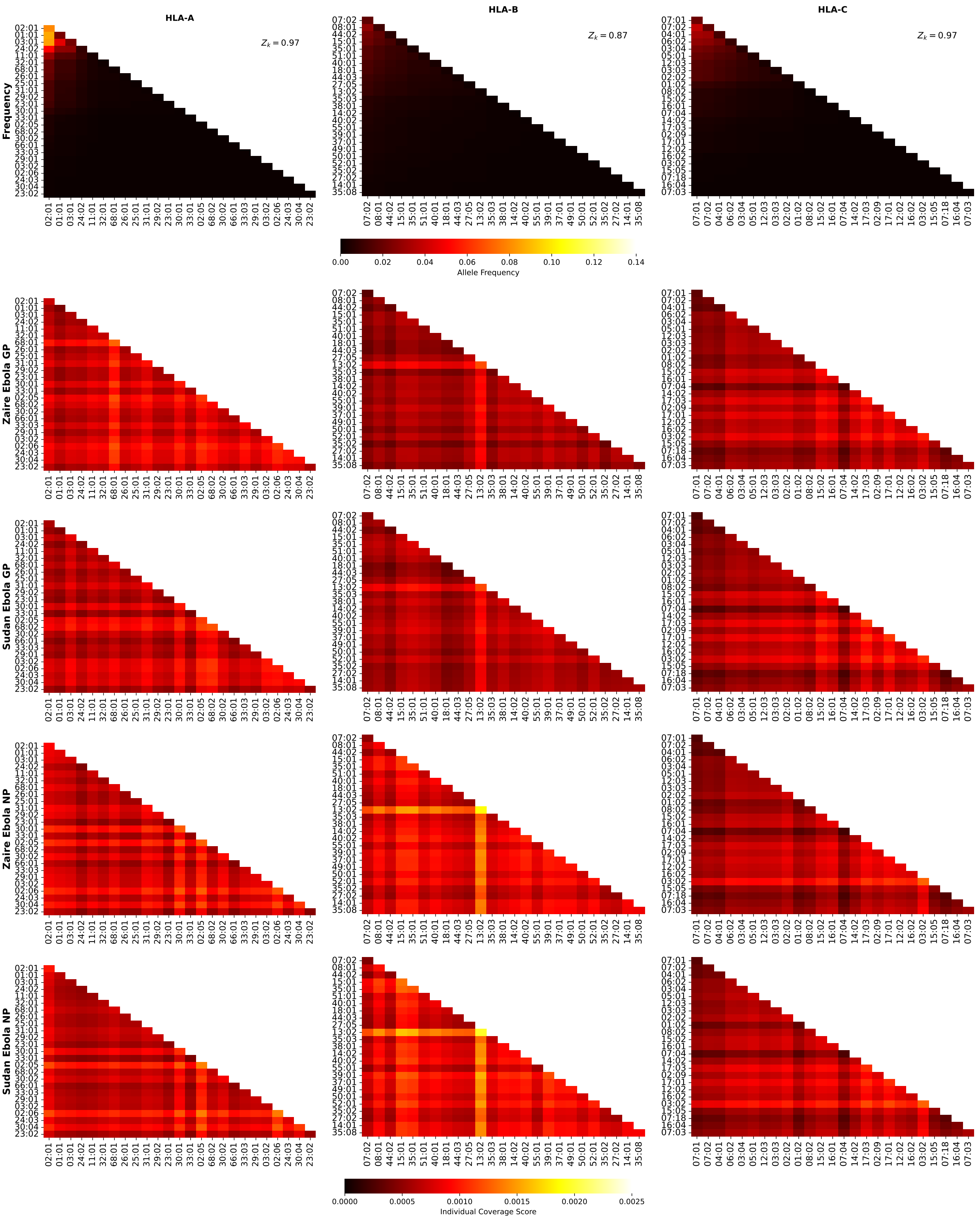

Figure S48. Frequencies and Ebola coverage scores for individuals in Europe. The 1st row corresponds to allele frequencies, the 2nd to GP1 Zaire, the 3rd to GP1 Sudan, the 4th to NP Zaire, and the 5th to NP Sudan. The 1st column is associated with HLA-A alleles, the 2nd to HLA-B, and the 3rd to HLA-C. The sum of the individual frequencies for each allele type is indicated on the panels in the 1st row.

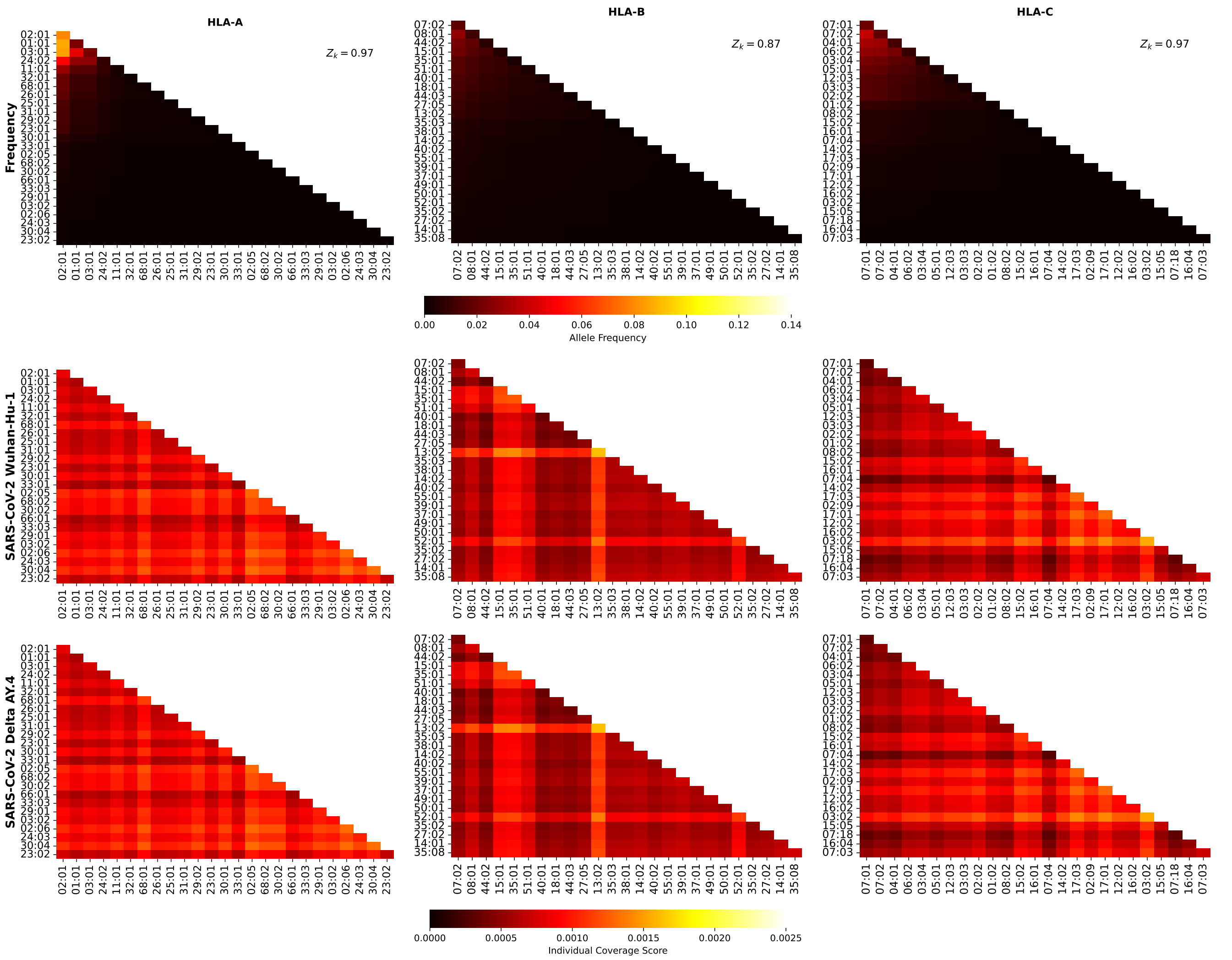

Figure S49. Frequencies and SARS-CoV-2 (Wuhan-Hu-1 and Delta AY.4 variants) coverage scores for individuals in Europe. The 1st row corresponds to allele frequencies, the 2nd to Wuhan-Hu-1, and the 3rd to Delta AY.4. The 1st column is associated with HLA-A alleles, the 2nd to HLA-B, and the 3rd to HLA-C. The sum of the individual frequencies for each allele type is indicated on the panels in the 1st row.

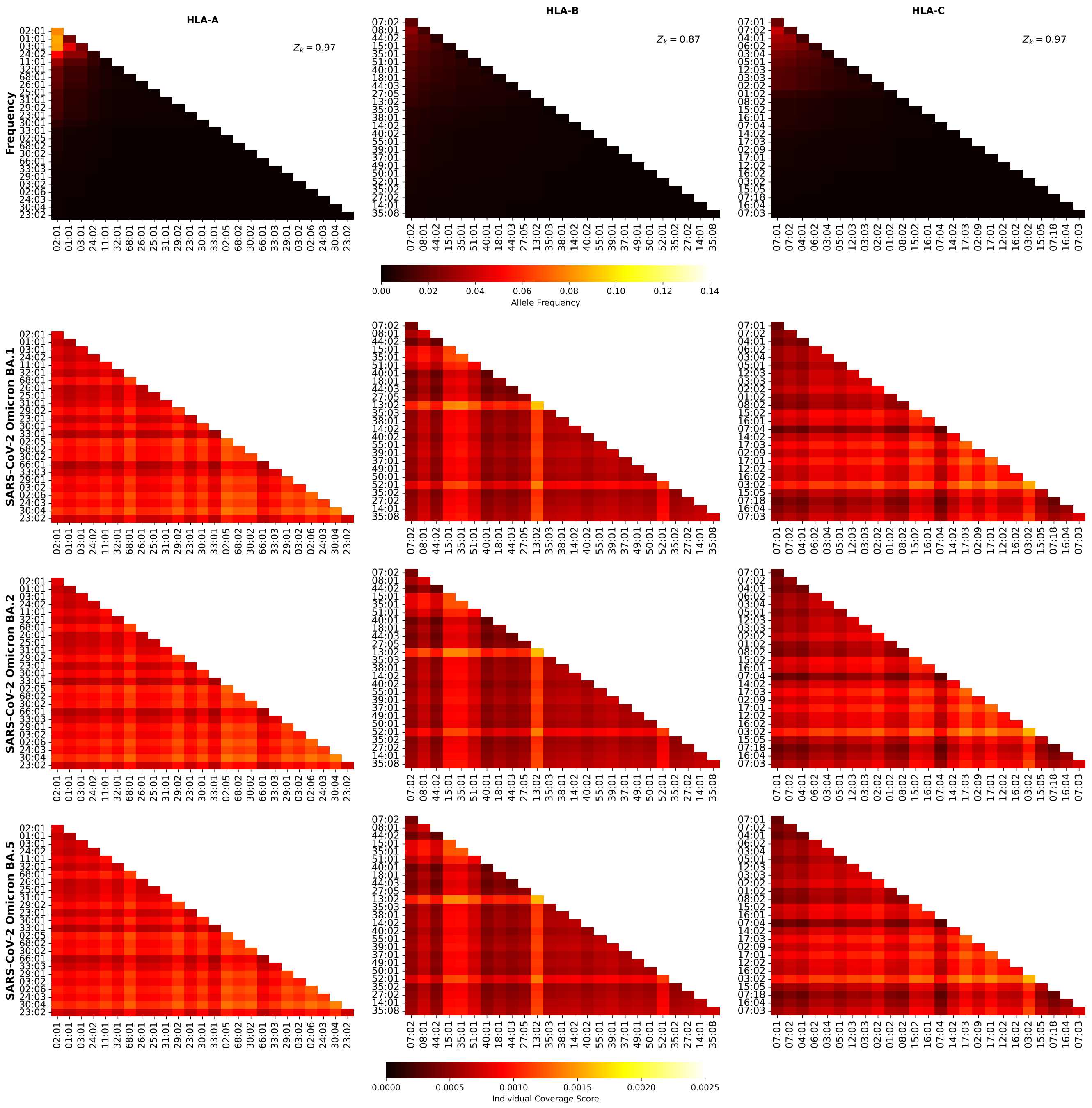

Figure S50. Frequencies and SARS-CoV-2 (Omicron variants) coverage scores for individuals in Europe. The 1st row corresponds to allele frequencies, the 2nd to BA.1, and the 3rd to BA.2, and the 4th to BA.5. The 1st column is associated with HLA-A alleles, the 2nd to HLA-B, and the 3rd to HLA-C. The sum of the individual frequencies for each allele type is indicated on the panels in the 1st row.

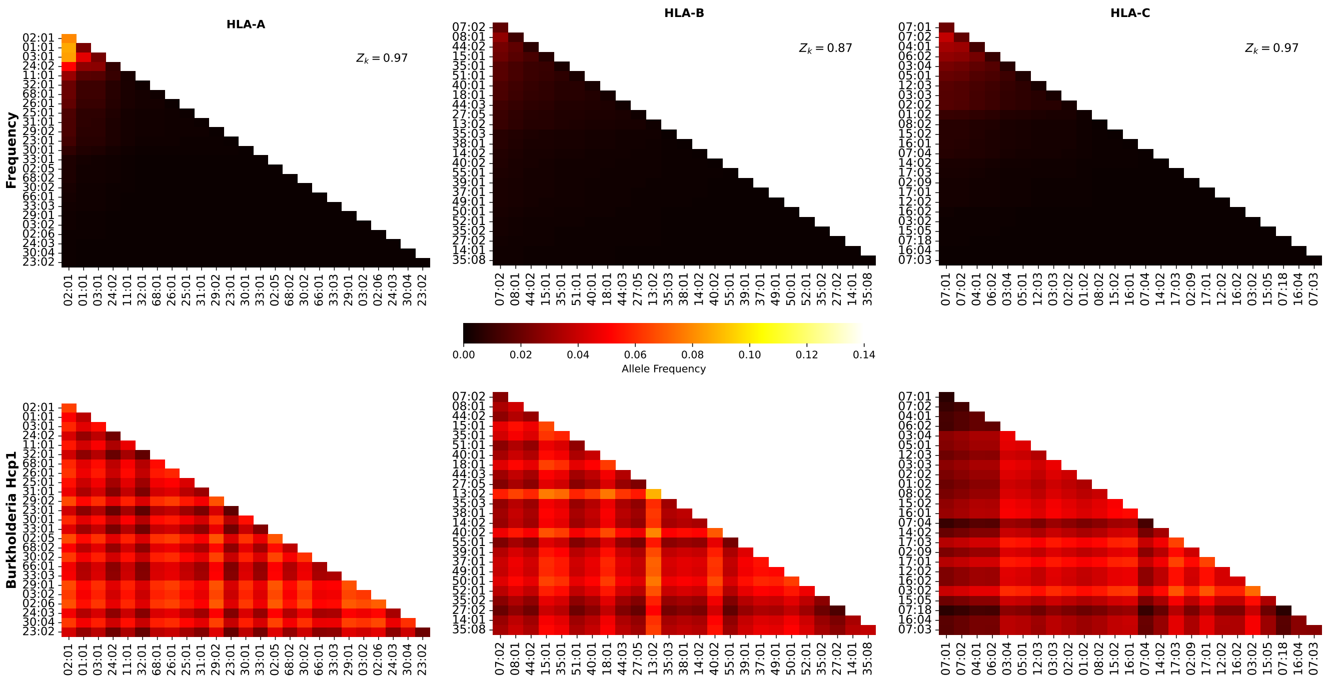

Figure S51. Frequencies and Burkholderia coverage scores for individuals in Europe. The 1st row corresponds to allele frequencies and the 2nd row to Burkholderia coverage score. The 1st column is associated with HLA-A alleles, the 2nd to HLA-B, and the 3rd to HLA-C. The sum of the individual frequencies for each allele type is indicated on the panels in the 1st row.

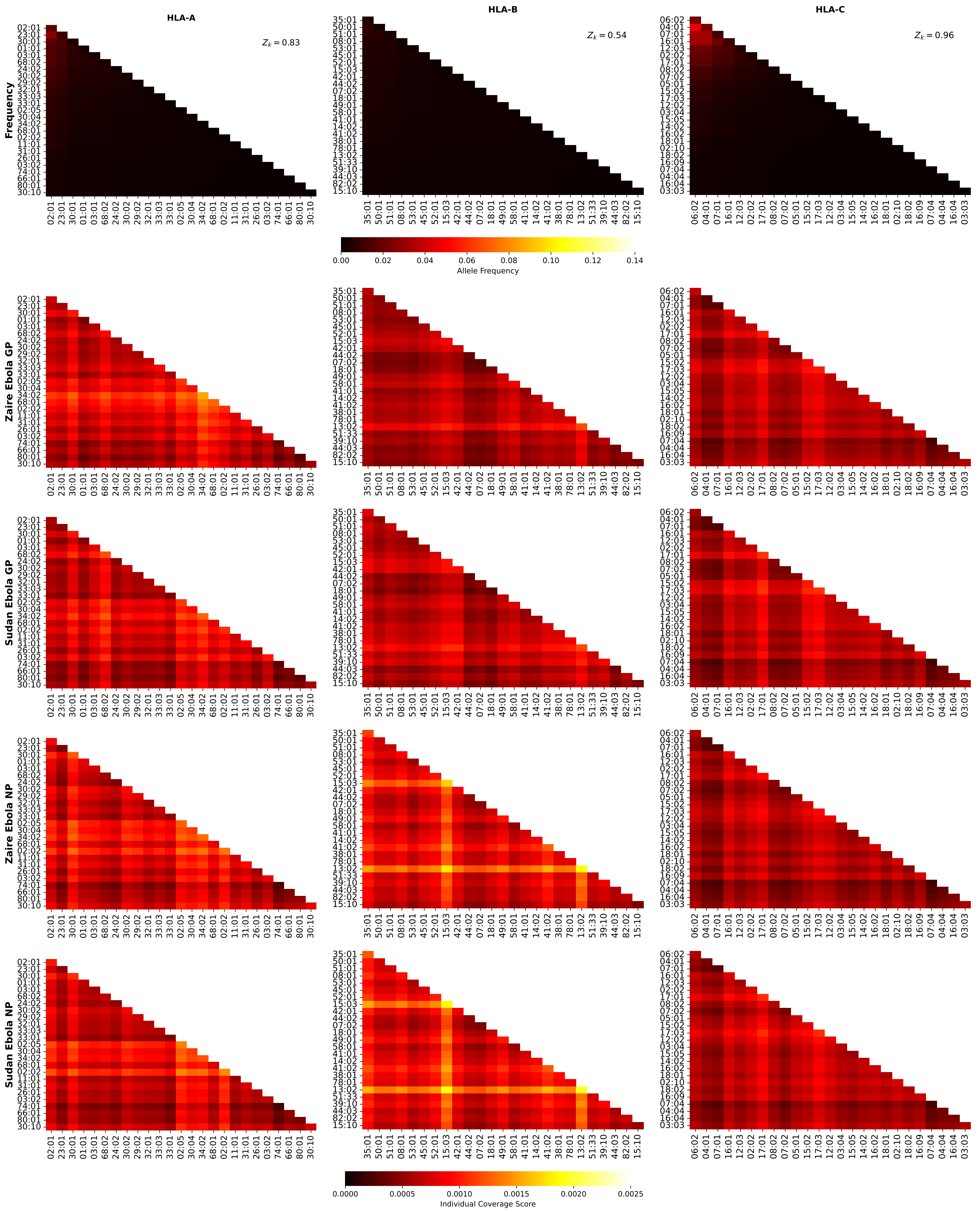

Figure S52. Frequencies and Ebola coverage scores for individuals in North Africa. The 1st row corresponds to allele frequencies, the 2nd to GP1 Zaire, the 3rd to GP1 Sudan, the 4th to NP Zaire, and the 5th to NP Sudan. The 1st column is associated with HLA-A alleles, the 2nd to HLA-B, and the 3rd to HLA-C. The sum of the individual frequencies for each allele type is indicated on the panels in the 1st row.

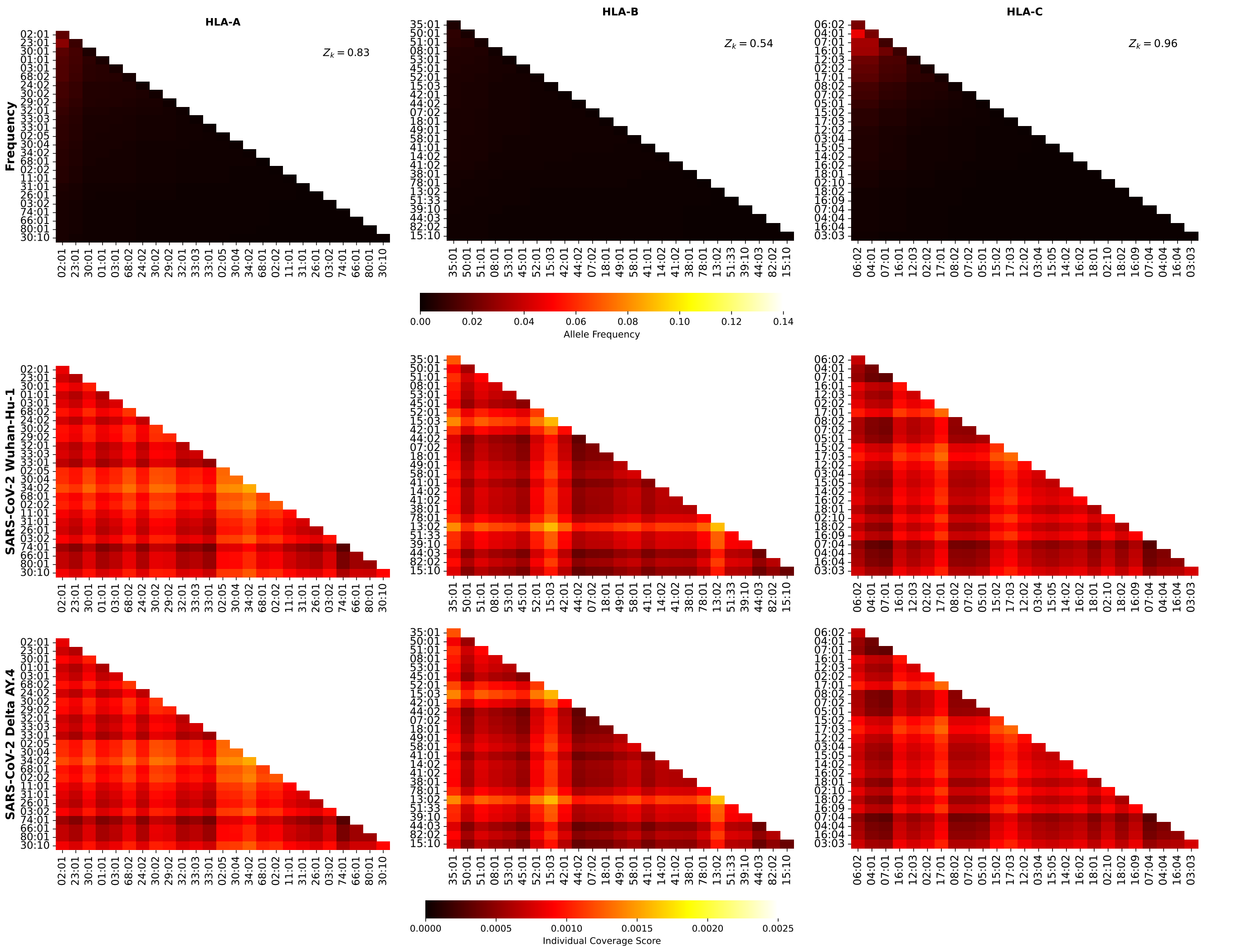

Figure S53. Frequencies and SARS-CoV-2 (Wuhan-Hu-1 and Delta AY.4 variants) coverage scores for individuals in North Africa. The 1st row corresponds to allele frequencies, the 2nd to Wuhan-Hu-1, and the 3rd to Delta AY.4. The 1st column is associated with HLA-A alleles, the 2nd to HLA-B, and the 3rd to HLA-C. The sum of the individual frequencies for each allele type is indicated on the panels in the 1st row.

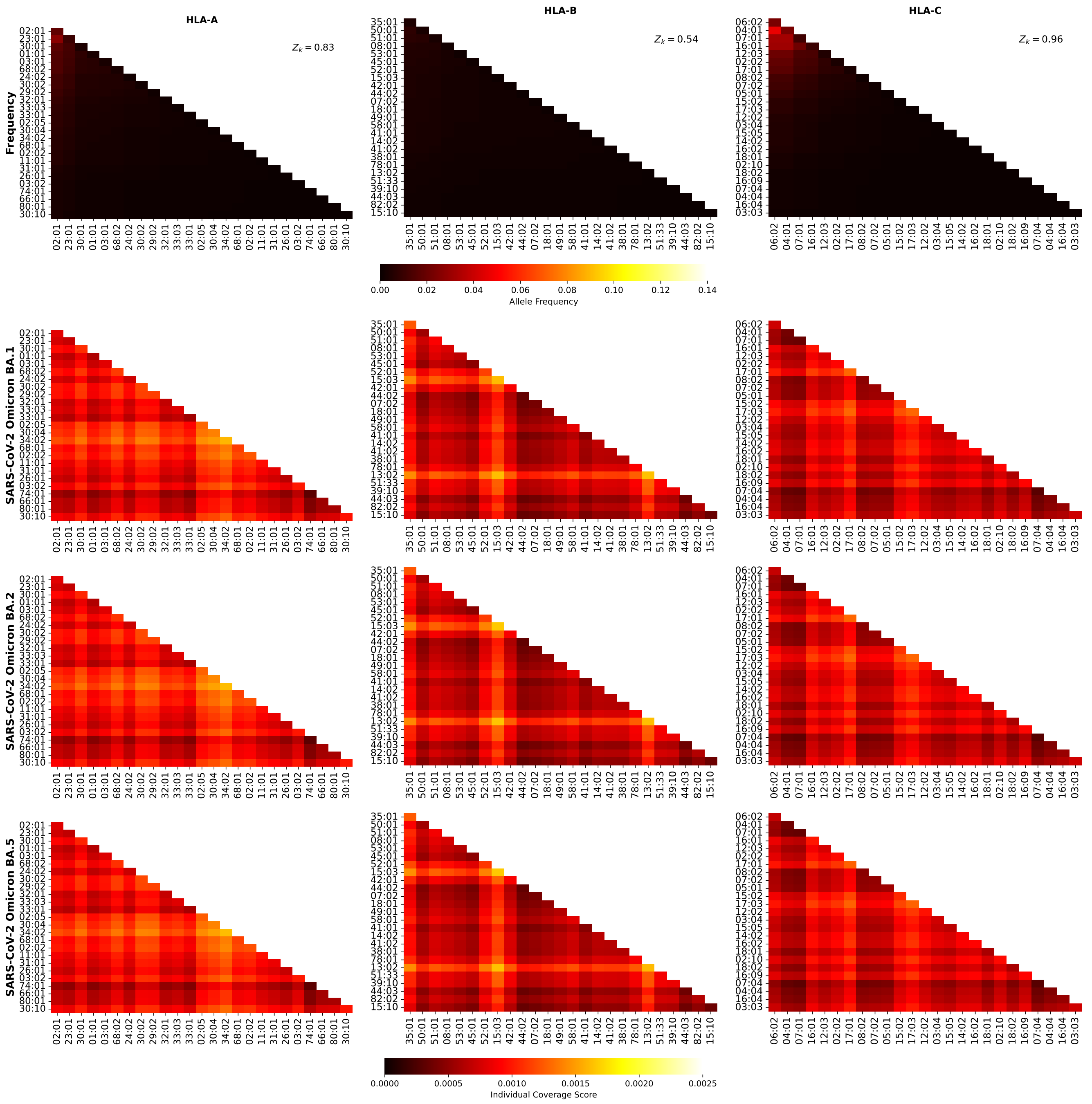

Figure S54. Frequencies and SARS-CoV-2 (Omicron variants) coverage scores for individuals in North Africa. The 1st row corresponds to allele frequencies, the 2nd to BA.1, and the 3rd to BA.2, and the 4th to BA.5. The 1st column is associated with HLA-A alleles, the 2nd to HLA-B, and the 3rd to HLA-C. The sum of the individual frequencies for each allele type is indicated on the panels in the 1st row.

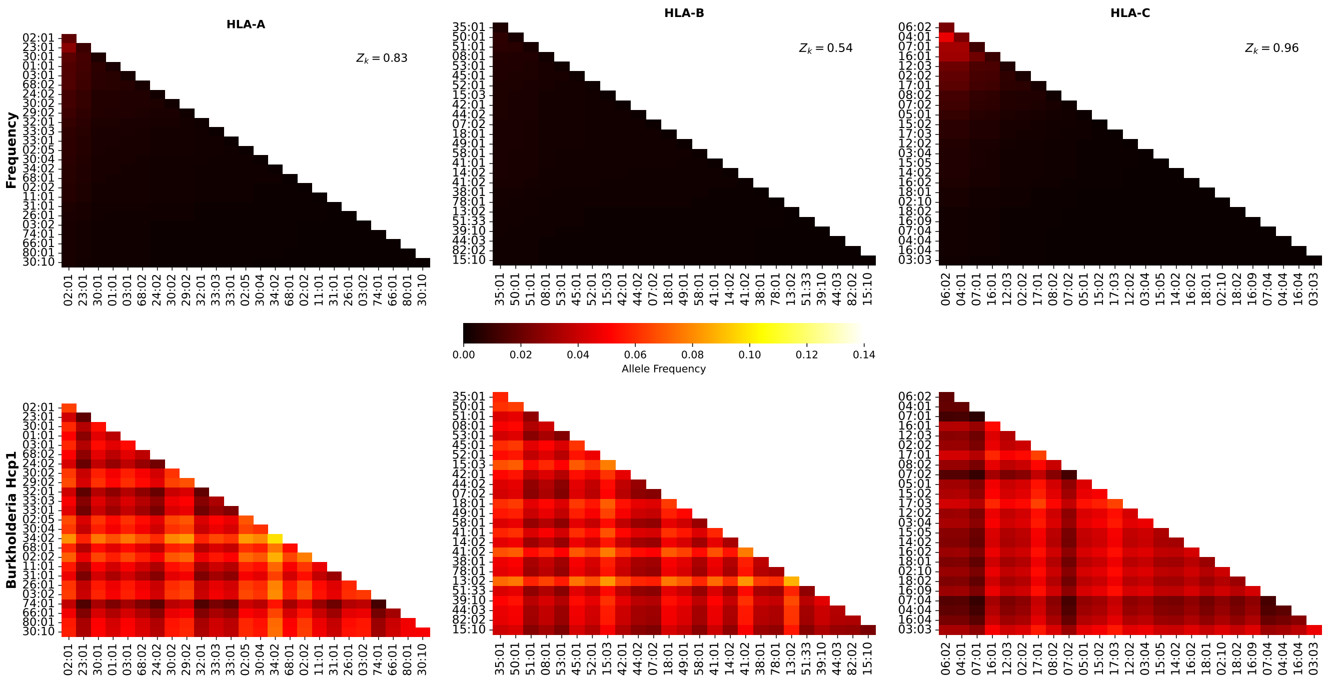

Figure S55. Frequencies and Burkholderia coverage scores for individuals in North Africa. The 1st row corresponds to allele frequencies and the 2nd row to Burkholderia coverage score. The 1st column is associated with HLA-A alleles, the 2nd to HLA-B, and the 3rd to HLA-C. The sum of the individual frequencies for each allele type is indicated on the panels in the 1st row.

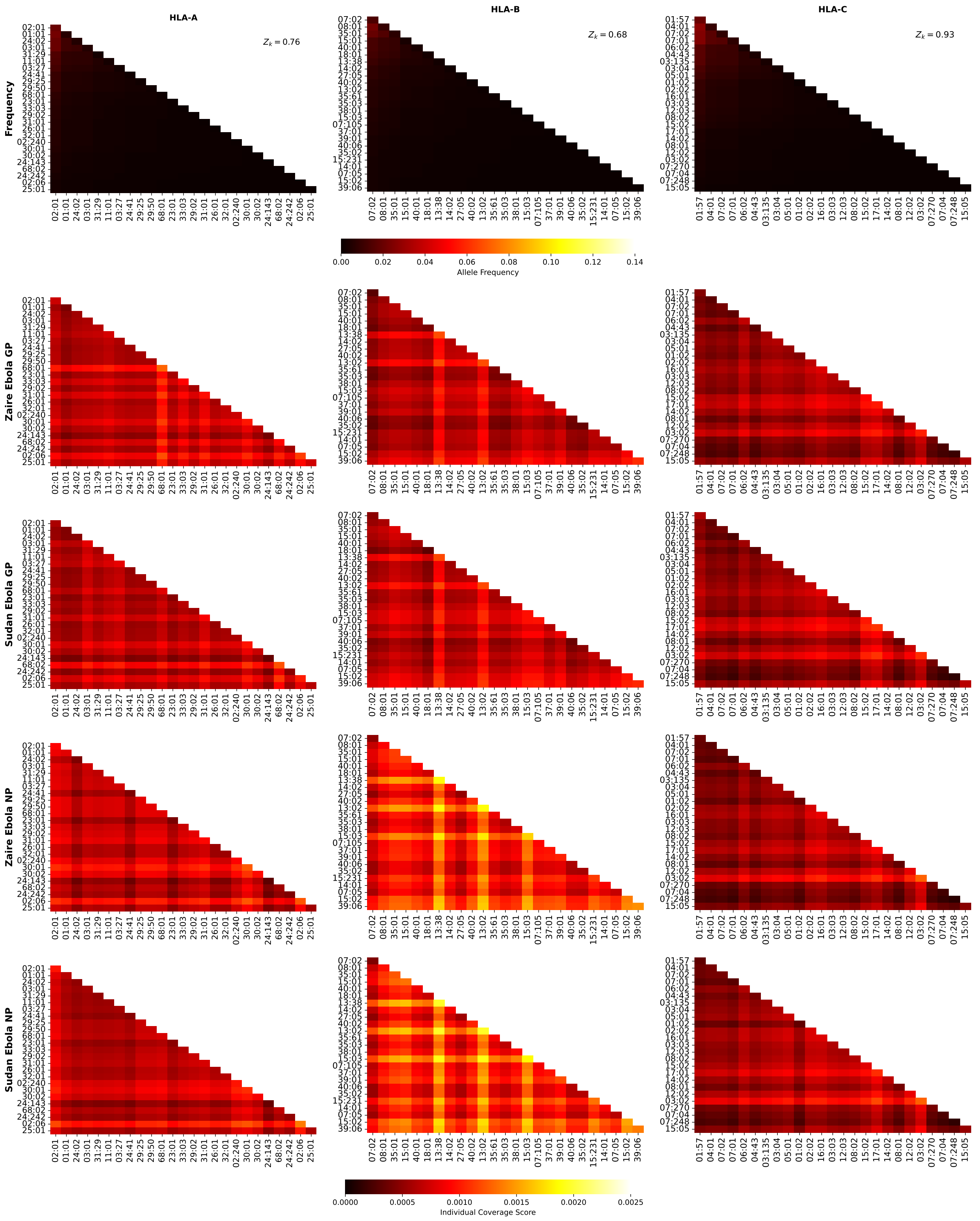

Figure S56. Frequencies and Ebola coverage scores for individuals in North America. The 1st row corresponds to allele frequencies, the 2nd to GP1 Zaire, the 3rd to GP1 Sudan, the 4th to NP Zaire, and the 5th to NP Sudan. The 1st column is associated with HLA-A alleles, the 2nd to HLA-B, and the 3rd to HLA-C. The sum of the individual frequencies for each allele type is indicated on the panels in the 1st row.

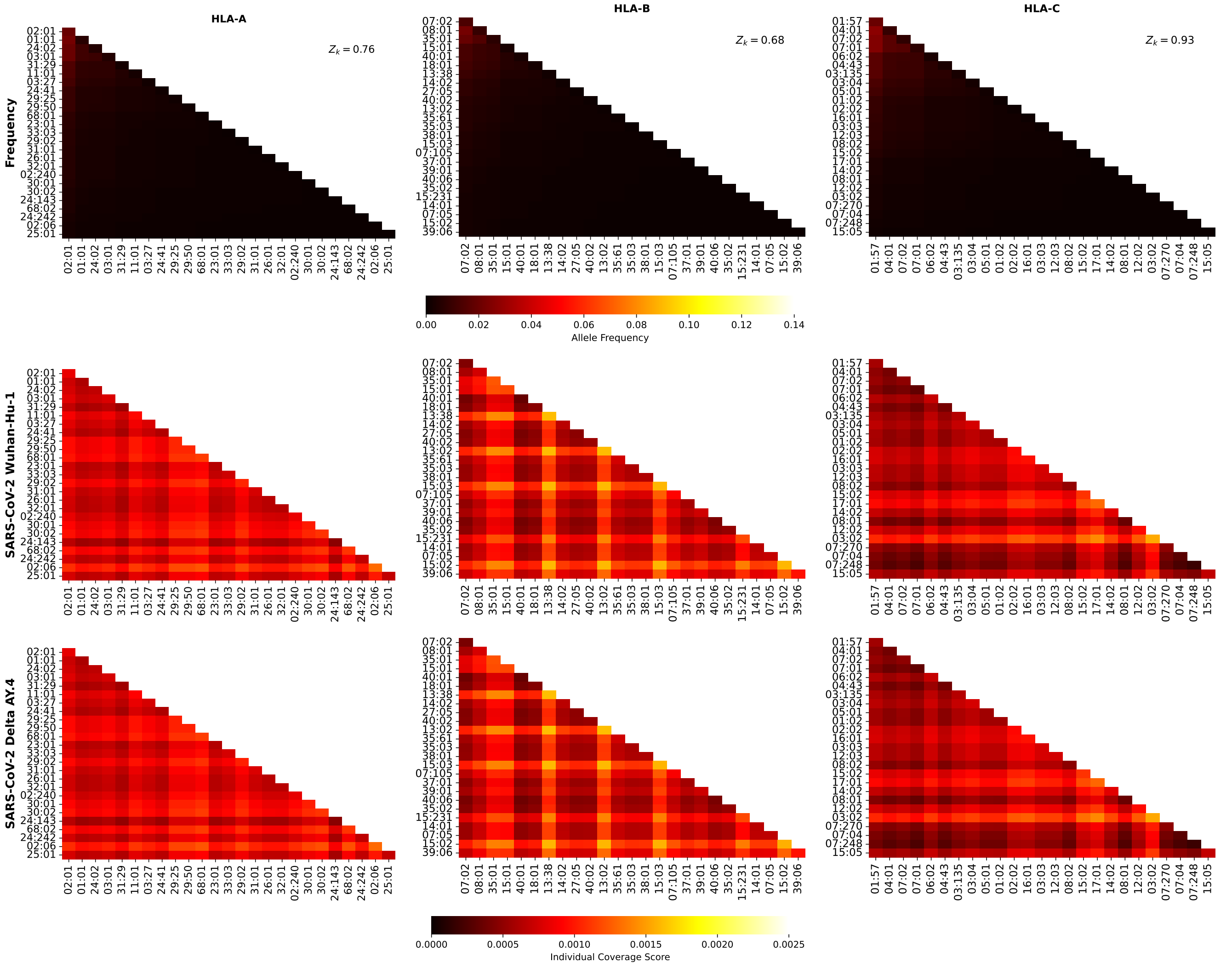

Figure S57. Frequencies and SARS-CoV-2 (Wuhan-Hu-1 and Delta AY.4 variants) coverage scores for individuals in North America. The 1st row corresponds to allele frequencies, the 2nd to Wuhan-Hu-1, and the 3rd to Delta AY.4. The 1st column is associated with HLA-A alleles, the 2nd to HLA-B, and the 3rd to HLA-C. The sum of the individual frequencies for each allele type is indicated on the panels in the 1st row.

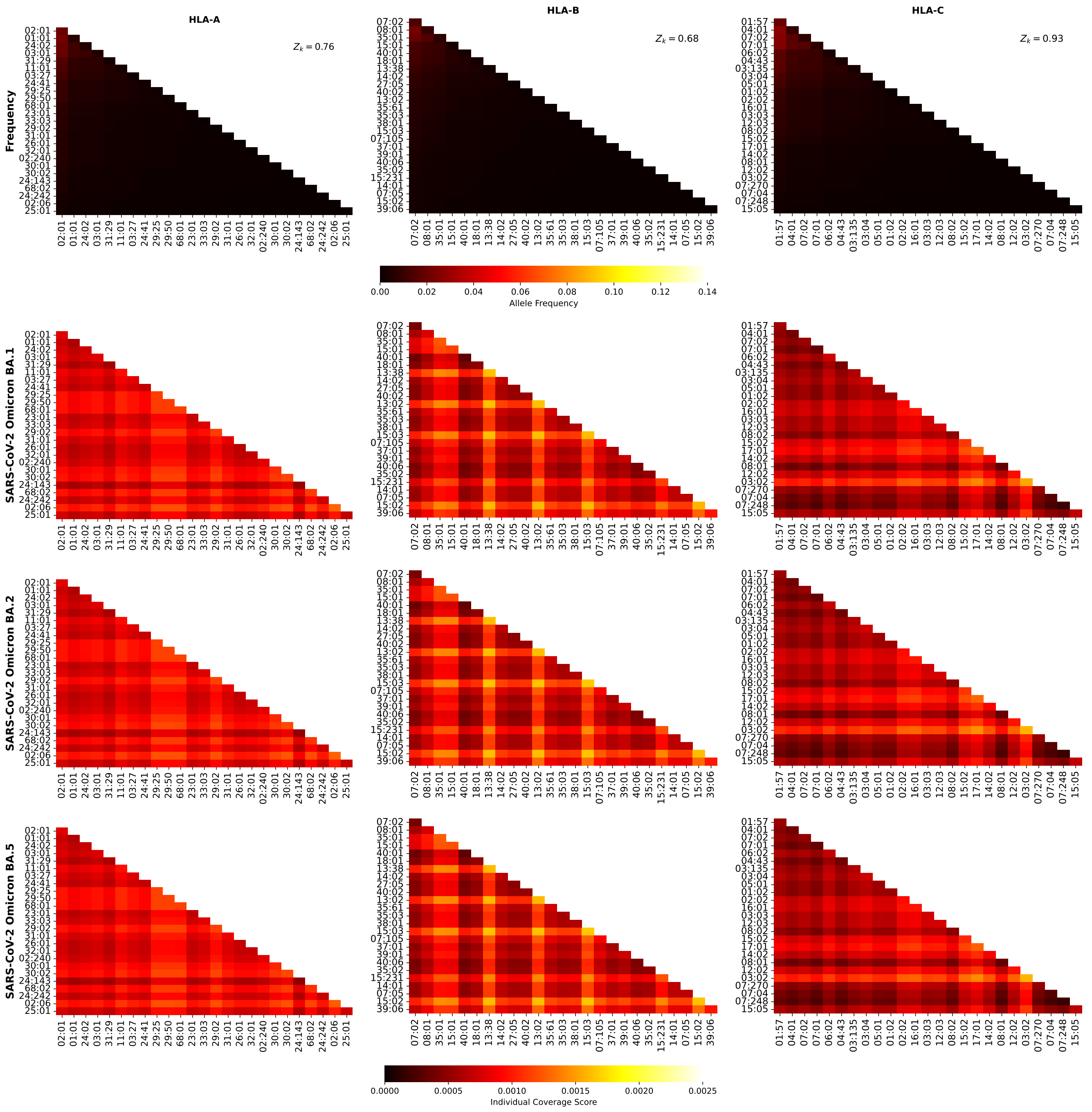

Figure S58. Frequencies and SARS-CoV-2 (Omicron variants) coverage scores for individuals in North America. The 1st row corresponds to allele frequencies, the 2nd to BA.1, and the 3rd to BA.2, and the 4th to BA.5. The 1st column is associated with HLA-A alleles, the 2nd to HLA-B, and the 3rd to HLA-C. The sum of the individual frequencies for each allele type is indicated on the panels in the 1st row.

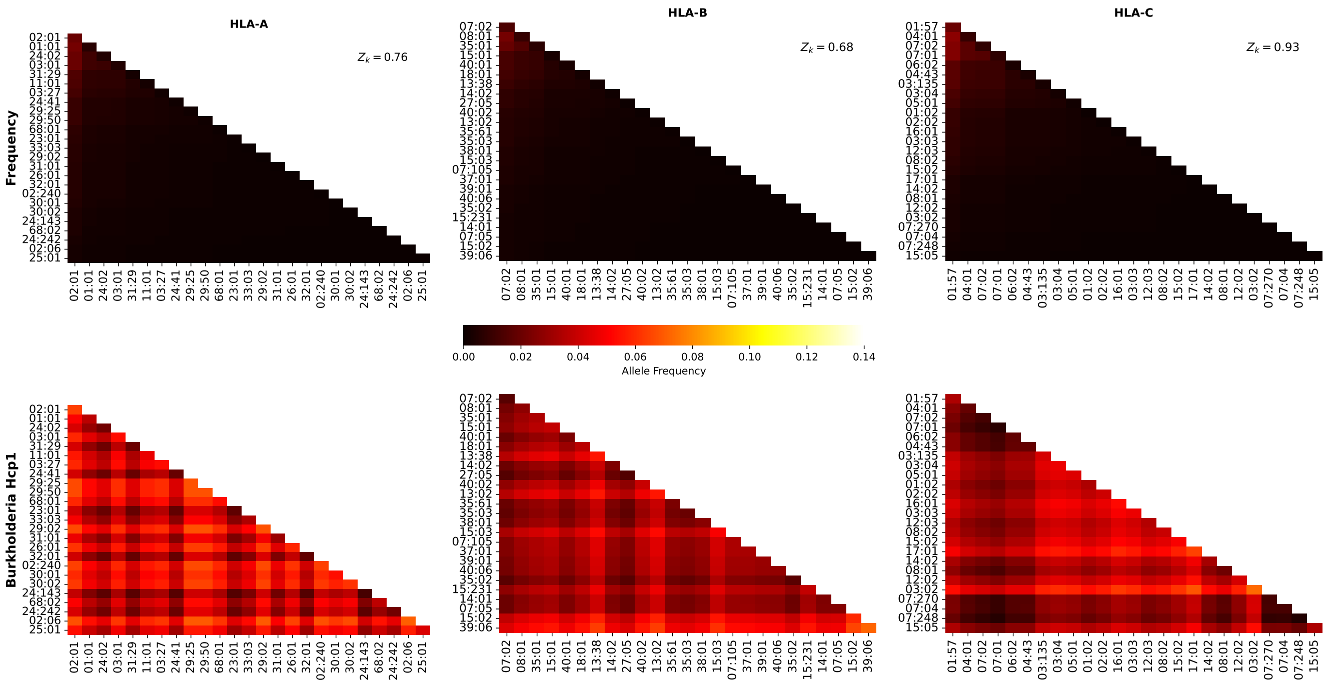

Figure S59. Frequencies and Burkholderia coverage scores for individuals in North America. The 1st row corresponds to allele frequencies and the 2nd row to Burkholderia coverage score. The 1st column is associated with HLA-A alleles, the 2nd to HLA-B, and the 3rd to HLA-C. The sum of the individual frequencies for each allele type is indicated on the panels in the 1st row.

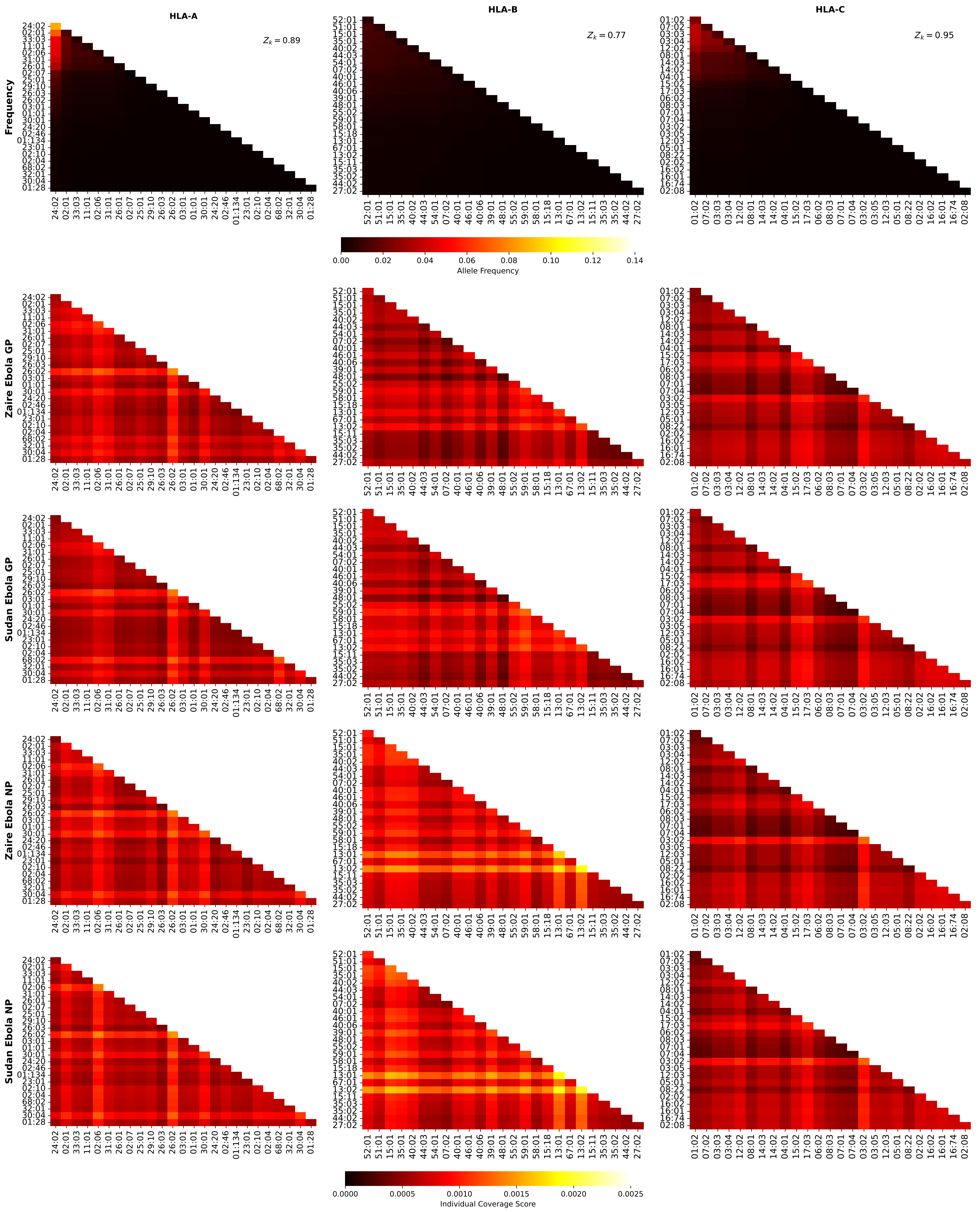

Figure S60. Frequencies and Ebola coverage scores for individuals in Northeast Asia. The 1st row corresponds to allele frequencies, the 2nd to GP1 Zaire, the 3rd to GP1 Sudan, the 4th to NP Zaire, and the 5th to NP Sudan. The 1st column is associated with HLA-A alleles, the 2nd to HLA-B, and the 3rd to HLA-C. The sum of the individual frequencies for each allele type is indicated on the panels in the 1st row.

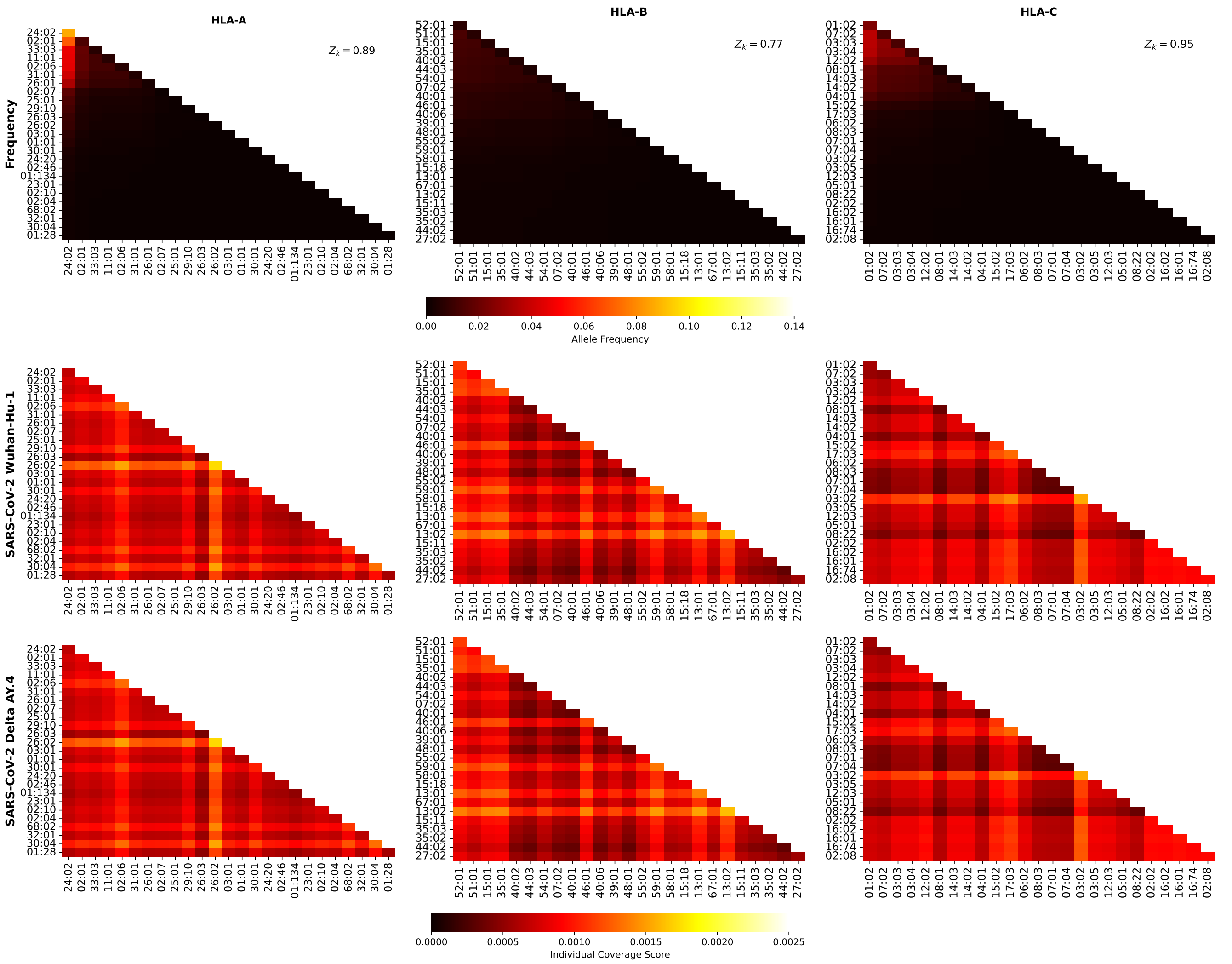

Figure S61. Frequencies and SARS-CoV-2 (Wuhan-Hu-1 and Delta AY.4 variants) coverage scores for individuals in Northeast Asia. The 1st row corresponds to allele frequencies, the 2nd to Wuhan-Hu-1, and the 3rd to Delta AY.4. The 1st column is associated with HLA-A alleles, the 2nd to HLA-B, and the 3rd to HLA-C. The sum of the individual frequencies for each allele type is indicated on the panels in the 1st row.

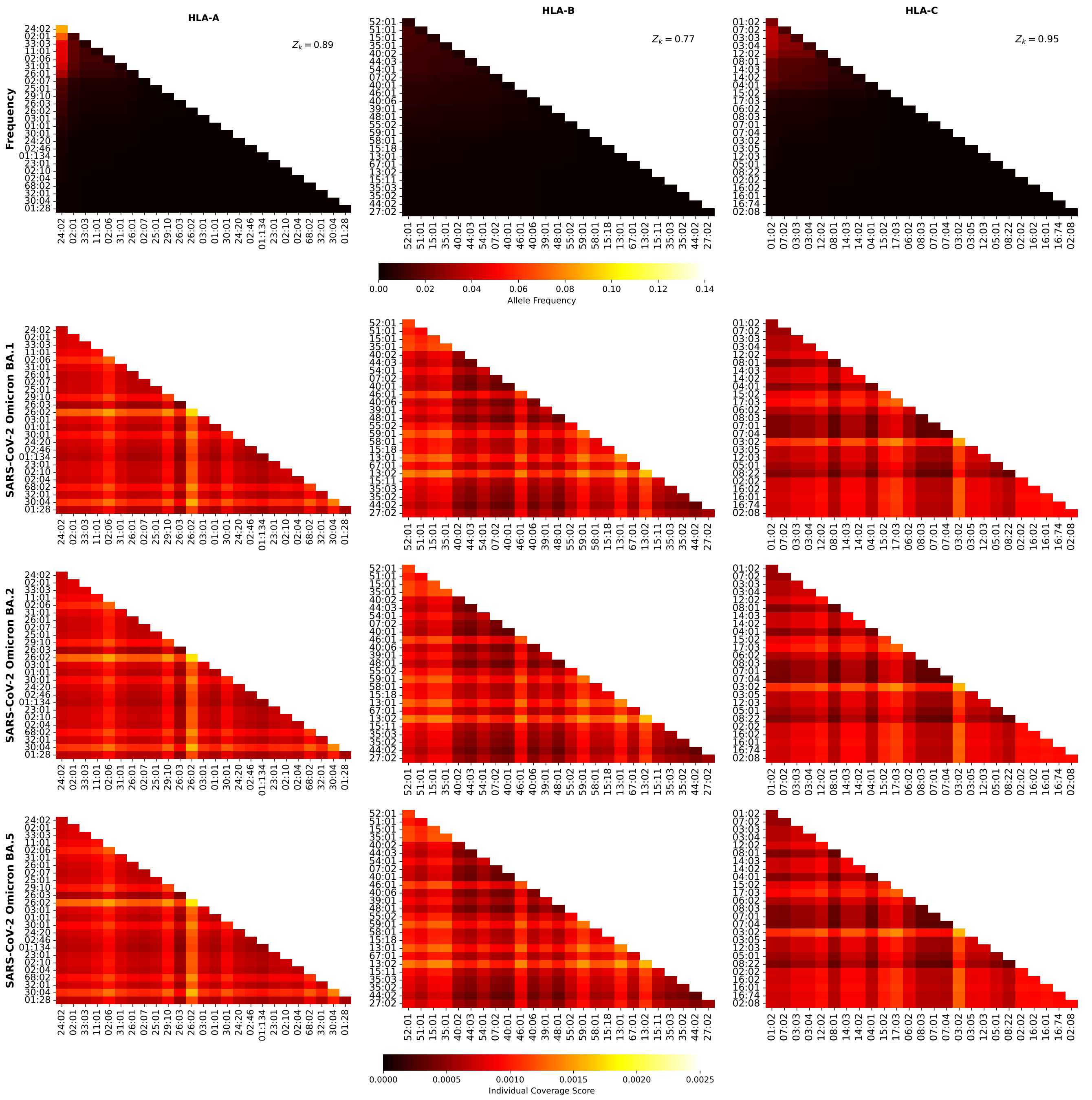

Figure S62. Frequencies and SARS-CoV-2 (Omicron variants) coverage scores for individuals in Northeast Asia. The 1st row corresponds to allele frequencies, the 2nd to BA.1, and the 3rd to BA.2, and the 4th to BA.5. The 1st column is associated with HLA-A alleles, the 2nd to HLA-B, and the 3rd to HLA-C. The sum of the individual frequencies for each allele type is indicated on the panels in the 1st row.

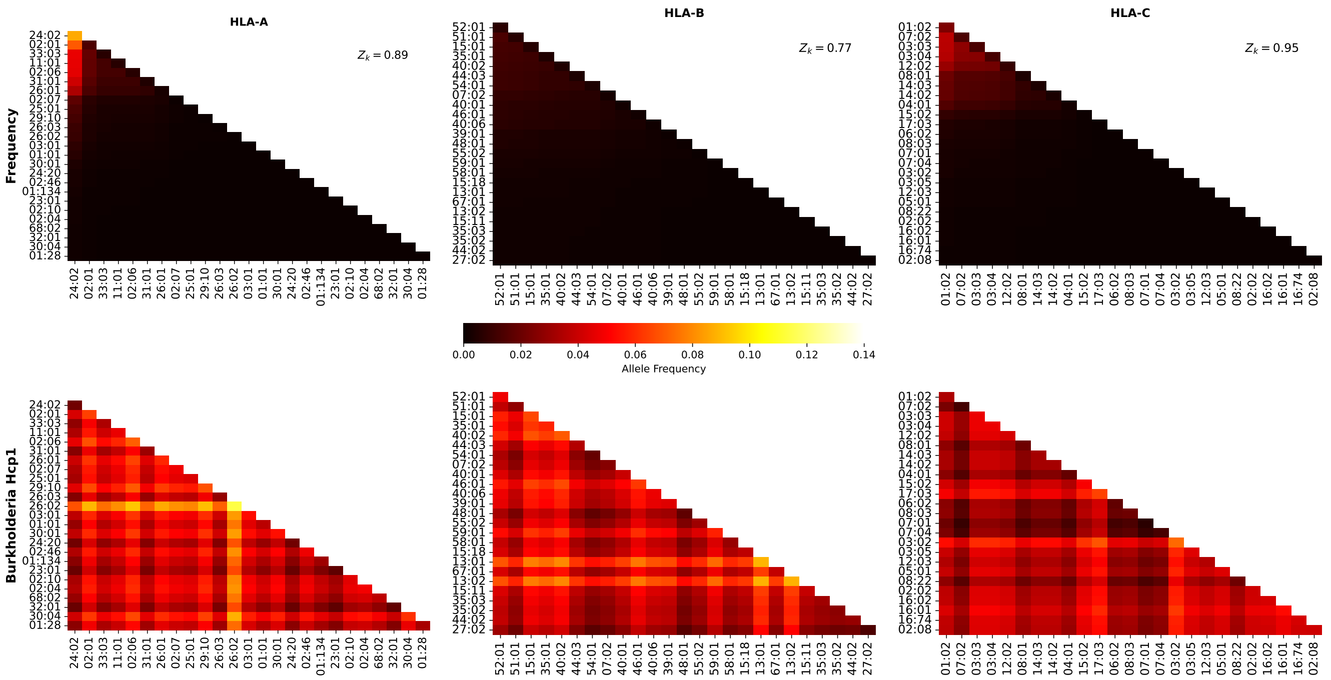

Figure S63. Frequencies and Burkholderia coverage scores for individuals in Northeast Asia. The 1st row corresponds to allele frequencies and the 2nd row to Burkholderia coverage score. The 1st column is associated with HLA-A alleles, the 2nd to HLA-B, and the 3rd to HLA-C. The sum of the individual frequencies for each allele type is indicated on the panels in the 1st row.

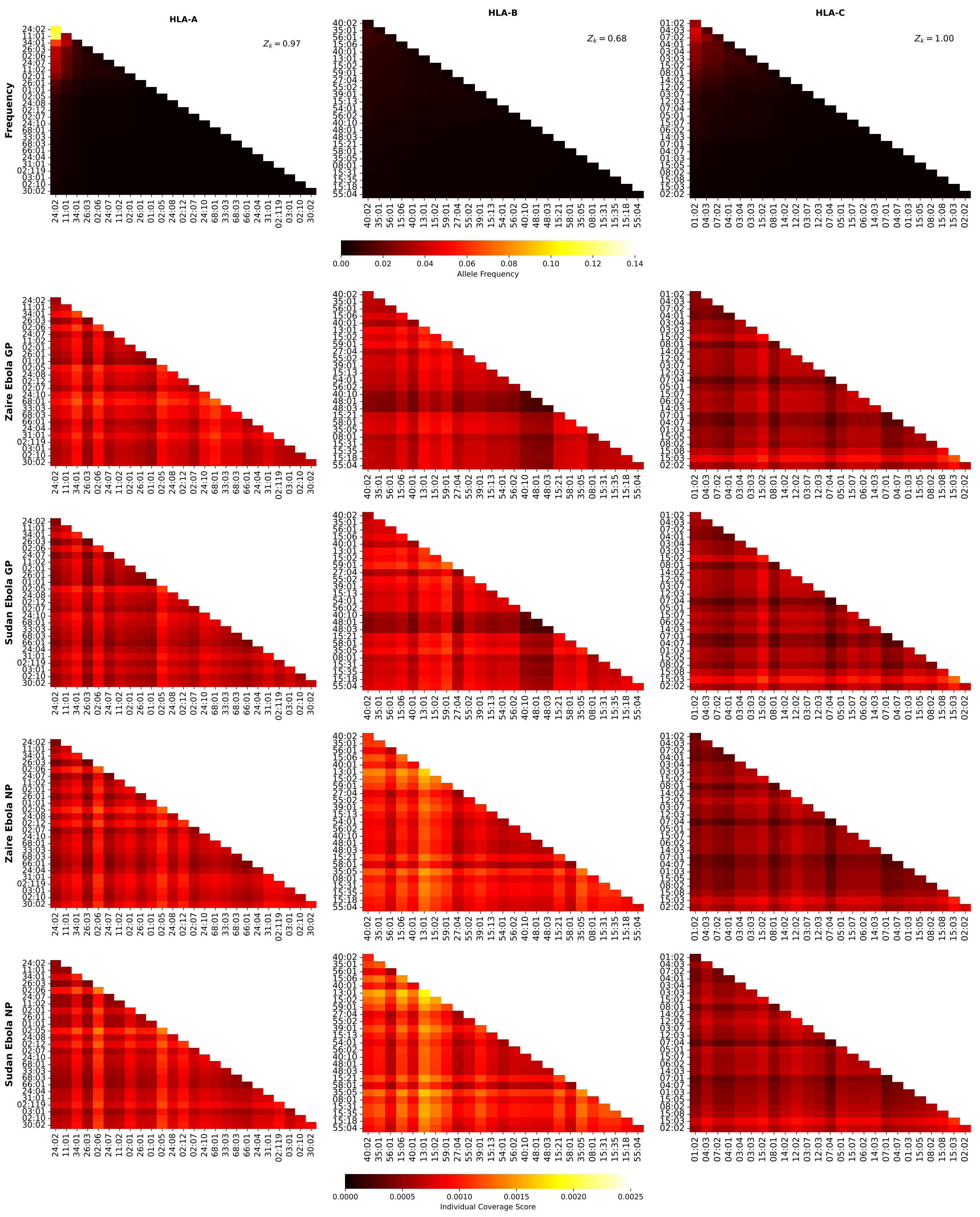

Figure S64. Frequencies and Ebola coverage scores for individuals in Oceania. The 1st row corresponds to allele frequencies, the 2nd to GP1 Zaire, the 3rd to GP1 Sudan, the 4th to NP Zaire, and the 5th to NP Sudan. The 1st column is associated with HLA-A alleles, the 2nd to HLA-B, and the 3rd to HLA-C. The sum of the individual frequencies for each allele type is indicated on the panels in the 1st row.

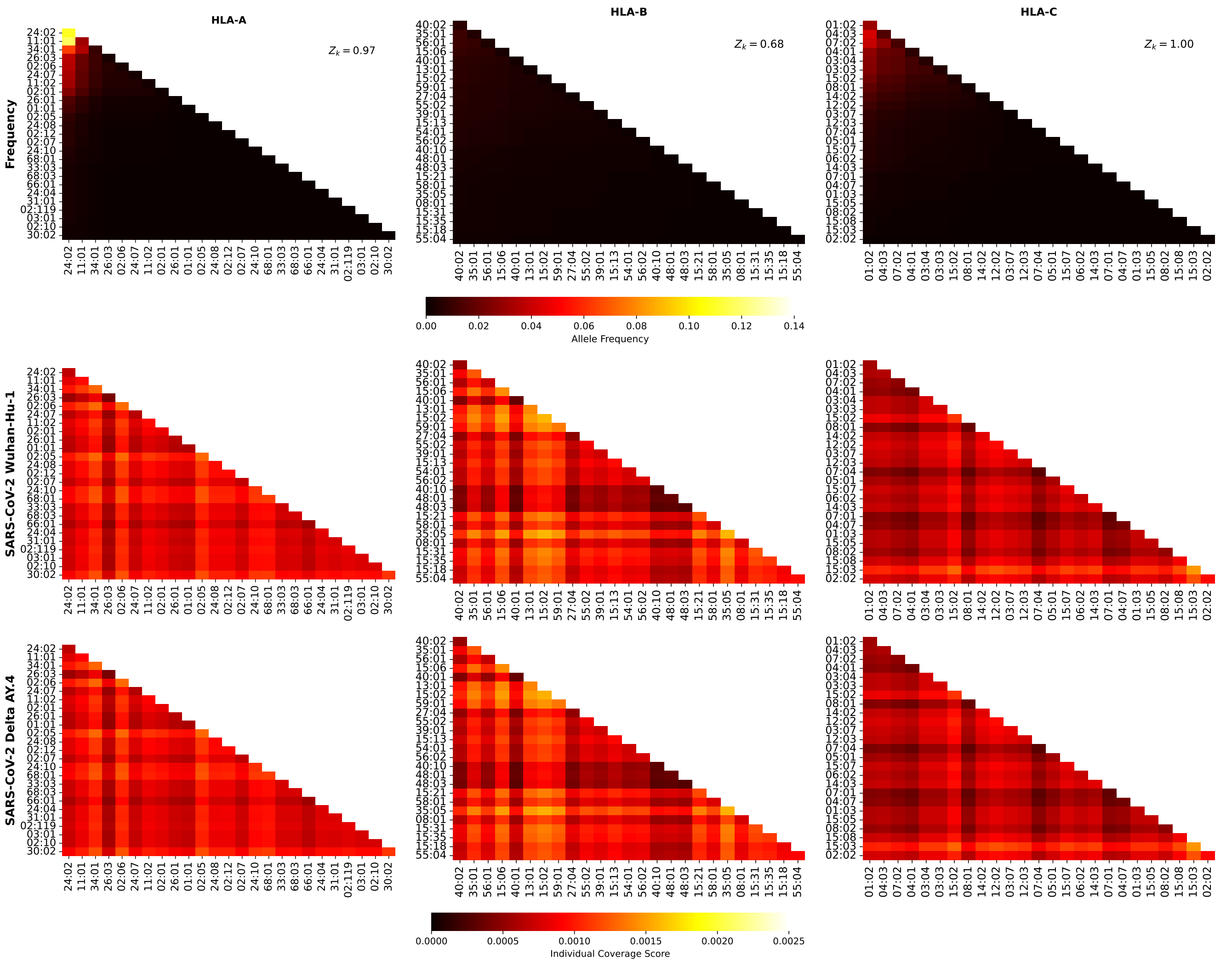

Figure S65. Frequencies and SARS-CoV-2 (Wuhan-Hu-1 and Delta AY.4 variants) coverage scores for individuals in Oceania. The 1st row corresponds to allele frequencies, the 2nd to Wuhan-Hu-1, and the 3rd to Delta AY.4. The 1st column is associated with HLA-A alleles, the 2nd to HLA-B, and the 3rd to HLA-C. The sum of the individual frequencies for each allele type is indicated on the panels in the 1st row.

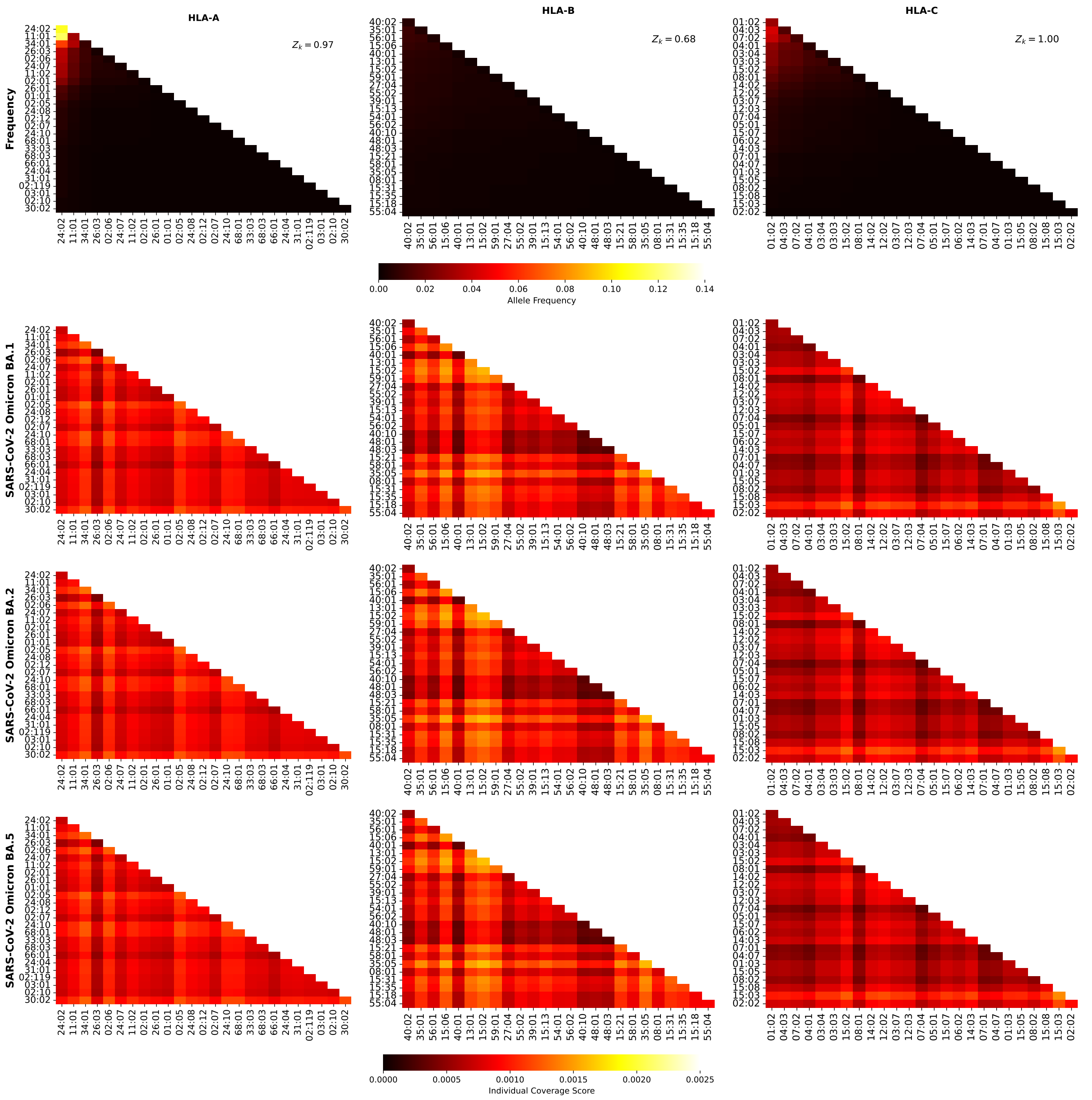

Figure S66. Frequencies and SARS-CoV-2 (Omicron variants) coverage scores for individuals in Oceania. The 1st row corresponds to allele frequencies, the 2nd to BA.1, and the 3rd to BA.2, and the 4th to BA.5. The 1st column is associated with HLA-A alleles, the 2nd to HLA-B, and the 3rd to HLA-C. The sum of the individual frequencies for each allele type is indicated on the panels in the 1st row.

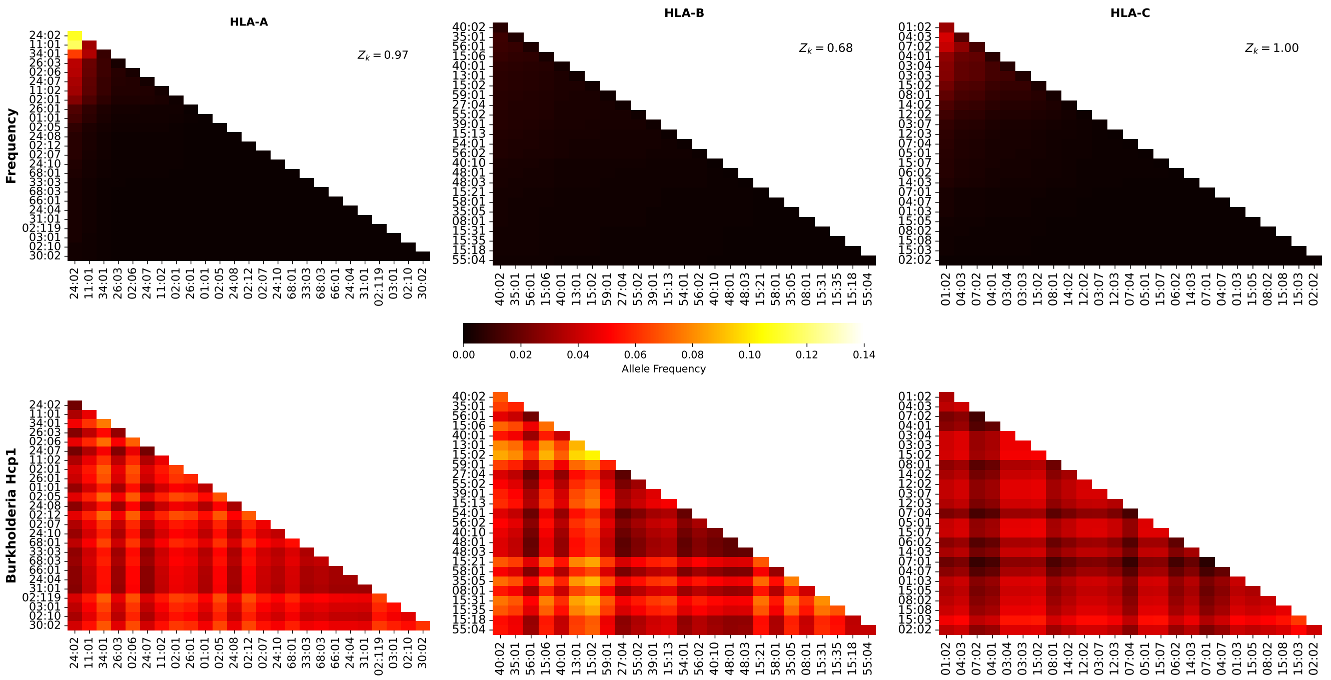

Figure S67. Frequencies and Burkholderia coverage scores for individuals in Oceania. The 1st row corresponds to allele frequencies and the 2nd row to Burkholderia coverage score. The 1st column is associated with HLA-A alleles, the 2nd to HLA-B, and the 3rd to HLA-C. The sum of the individual frequencies for each allele type is indicated on the panels in the 1st row.

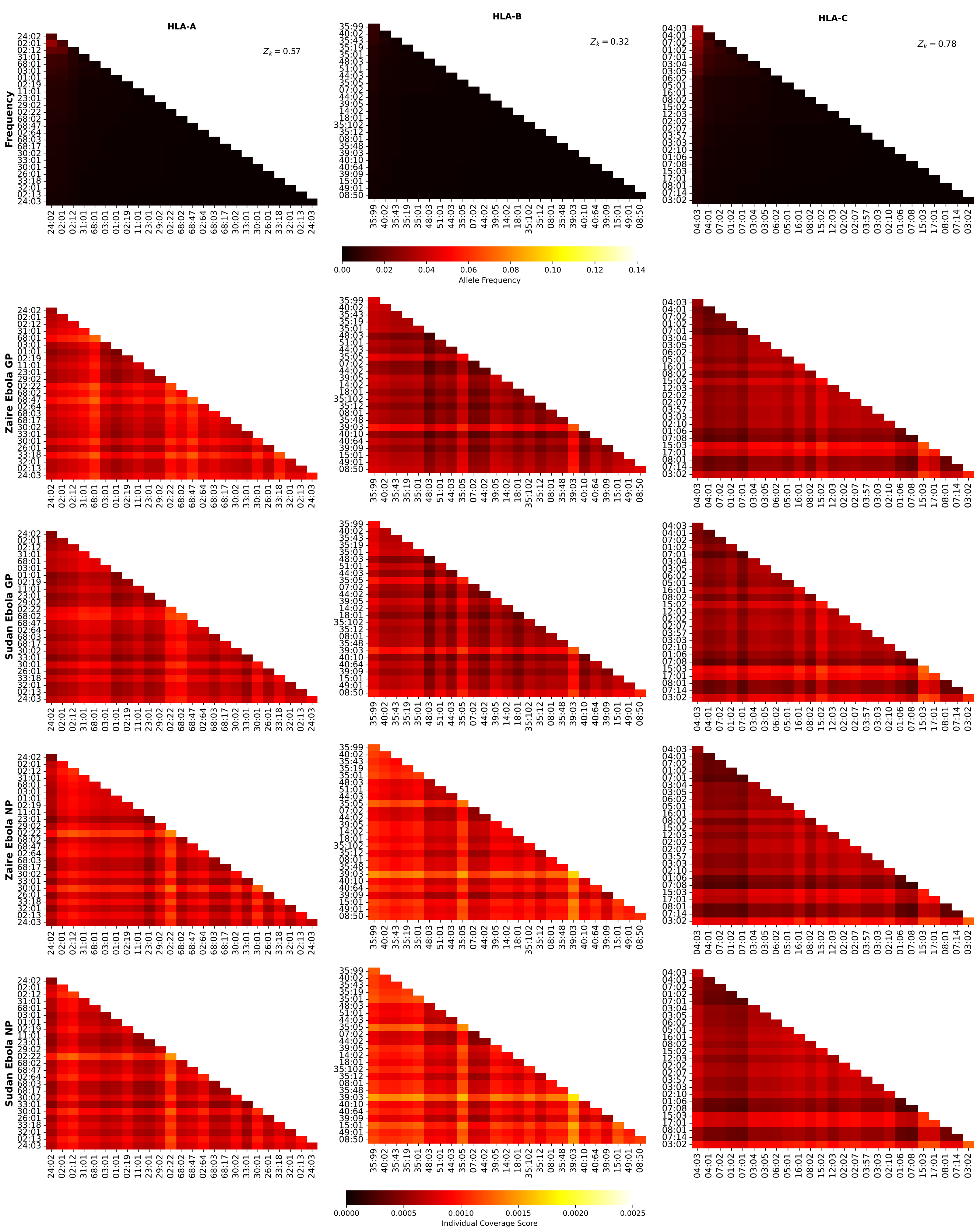

Figure S68. Frequencies and Ebola coverage scores for individuals in South and Central America. The 1st row corresponds to allele frequencies, the 2nd to GP1 Zaire, the 3rd to GP1 Sudan, the 4th to NP Zaire, and the 5th to NP Sudan. The 1st column is associated with HLA-A alleles, the 2nd to HLA-B, and the 3rd to HLA-C. The sum of the individual frequencies for each allele type is indicated on the panels in the 1st row.

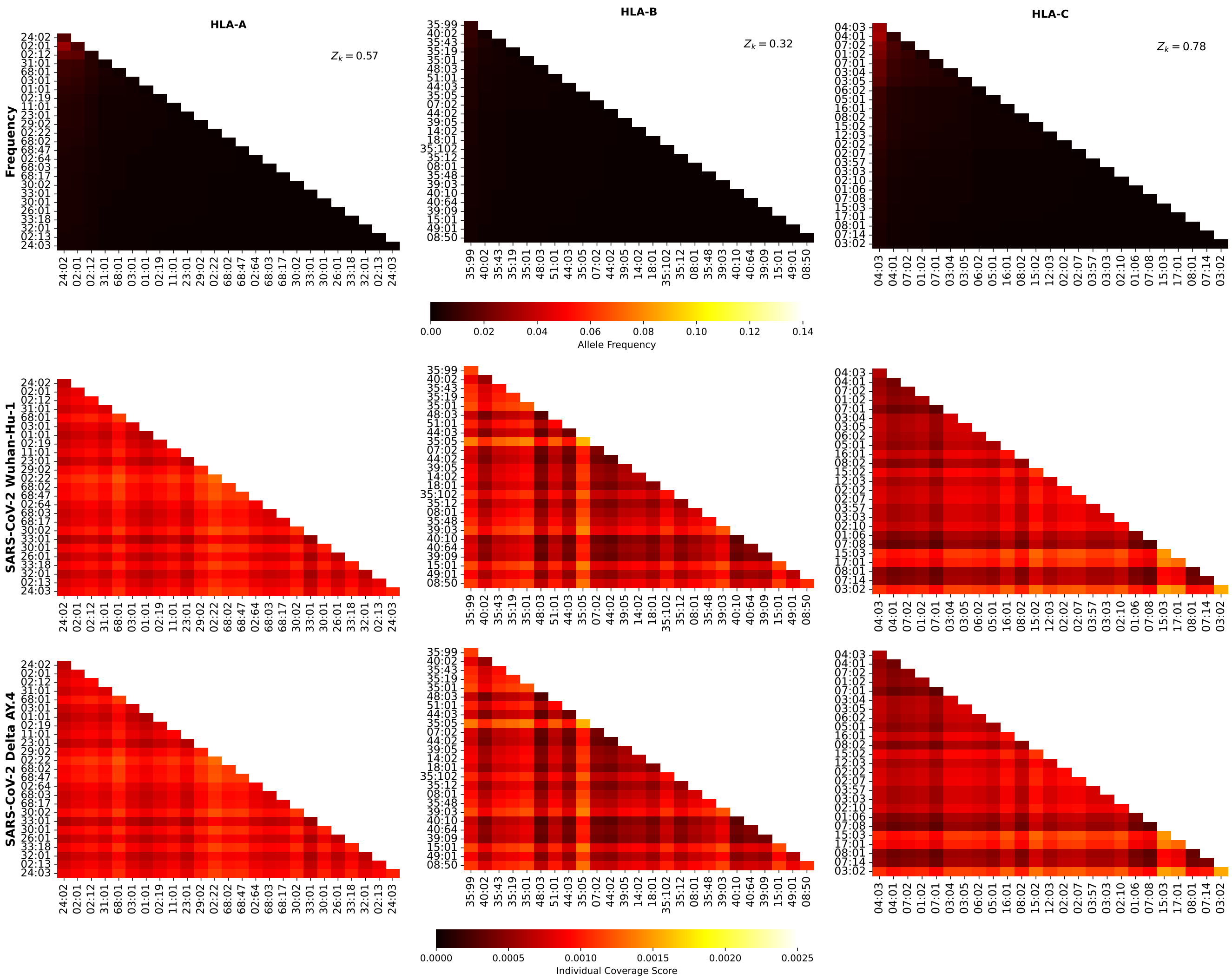

Figure S69. Frequencies and SARS-CoV-2 (Wuhan-Hu-1 and Delta AY.4 variants) coverage scores for individuals in South and Central America. The 1st row corresponds to allele frequencies, the 2nd to Wuhan-Hu-1, and the 3rd to Delta AY.4. The 1st column is associated with HLA-A alleles, the 2nd to HLA-B, and the 3rd to HLA-C. The sum of the individual frequencies for each allele type is indicated on the panels in the 1st row.

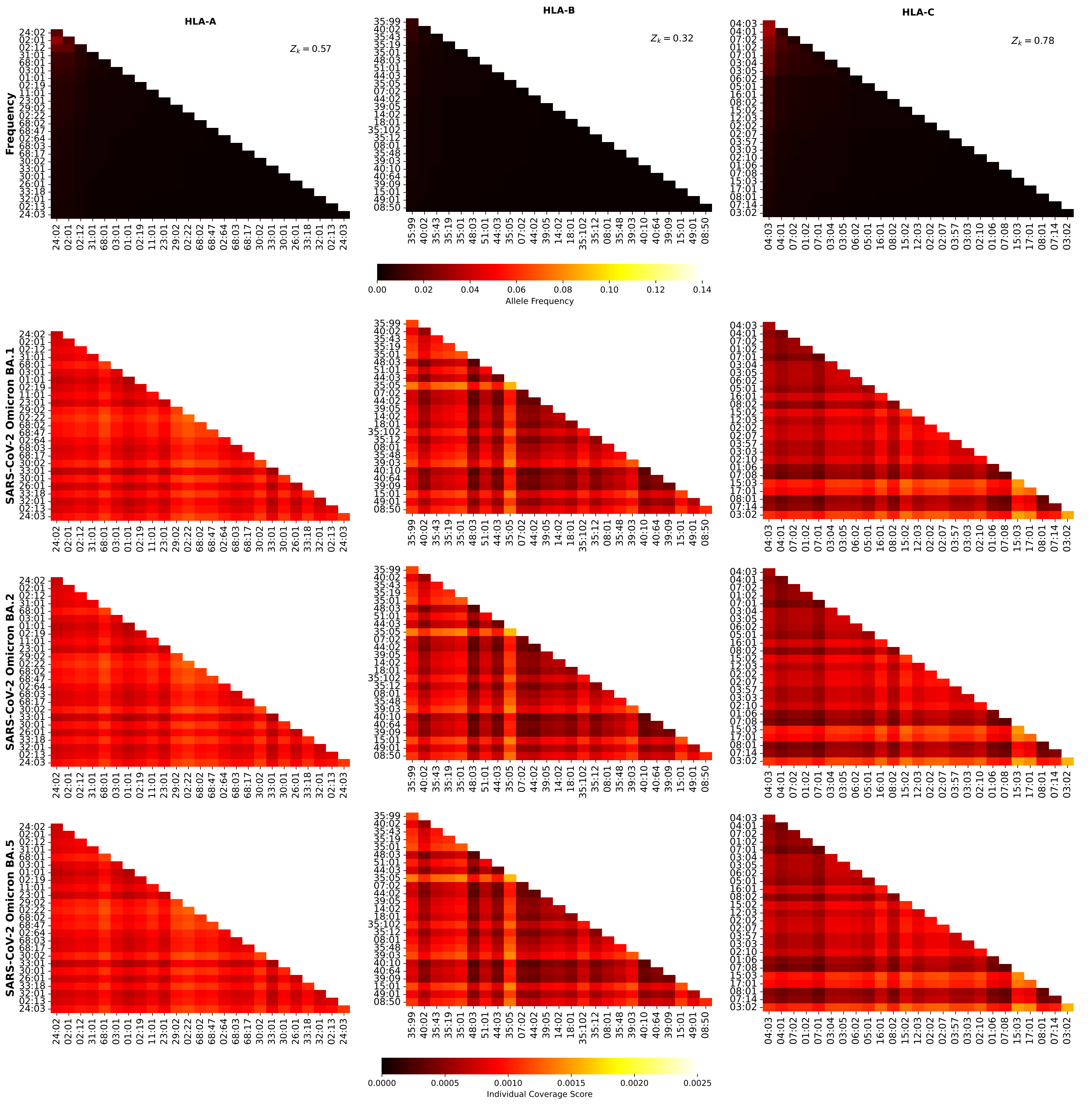

Figure S70. Frequencies and SARS-CoV-2 (Omicron variants) coverage scores for individuals in South and Central America. The 1st row corresponds to allele frequencies, the 2nd to BA.1, and the 3rd to BA.2, and the 4th to BA.5. The 1st column is associated with HLA-A alleles, the 2nd to HLA-B, and the 3rd to HLA-C. The sum of the individual frequencies for each allele type is indicated on the panels in the 1st row.

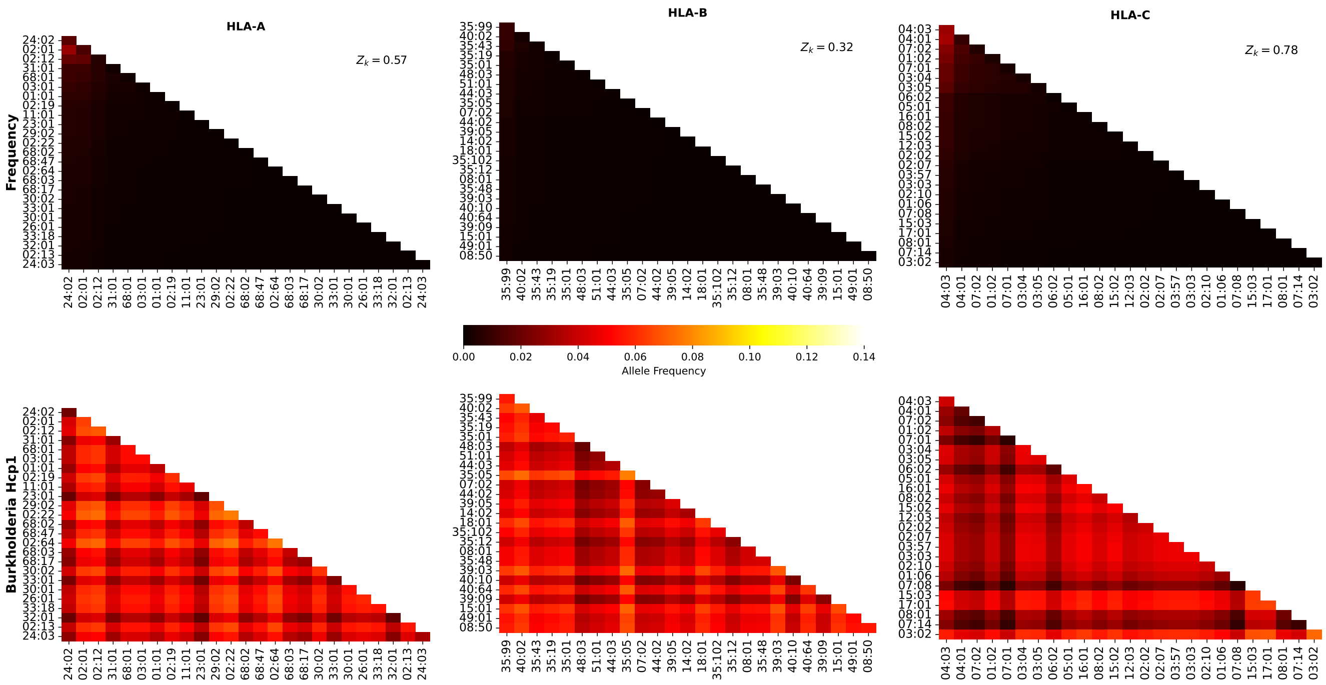

Figure S71. Frequencies and Burkholderia coverage scores for individuals in South and Central America. The 1st row corresponds to allele frequencies and the 2nd row to Burkholderia coverage score. The 1st column is associated with HLA-A alleles, the 2nd to HLA-B, and the 3rd to HLA-C. The sum of the individual frequencies for each allele type is indicated on the panels in the 1st row.

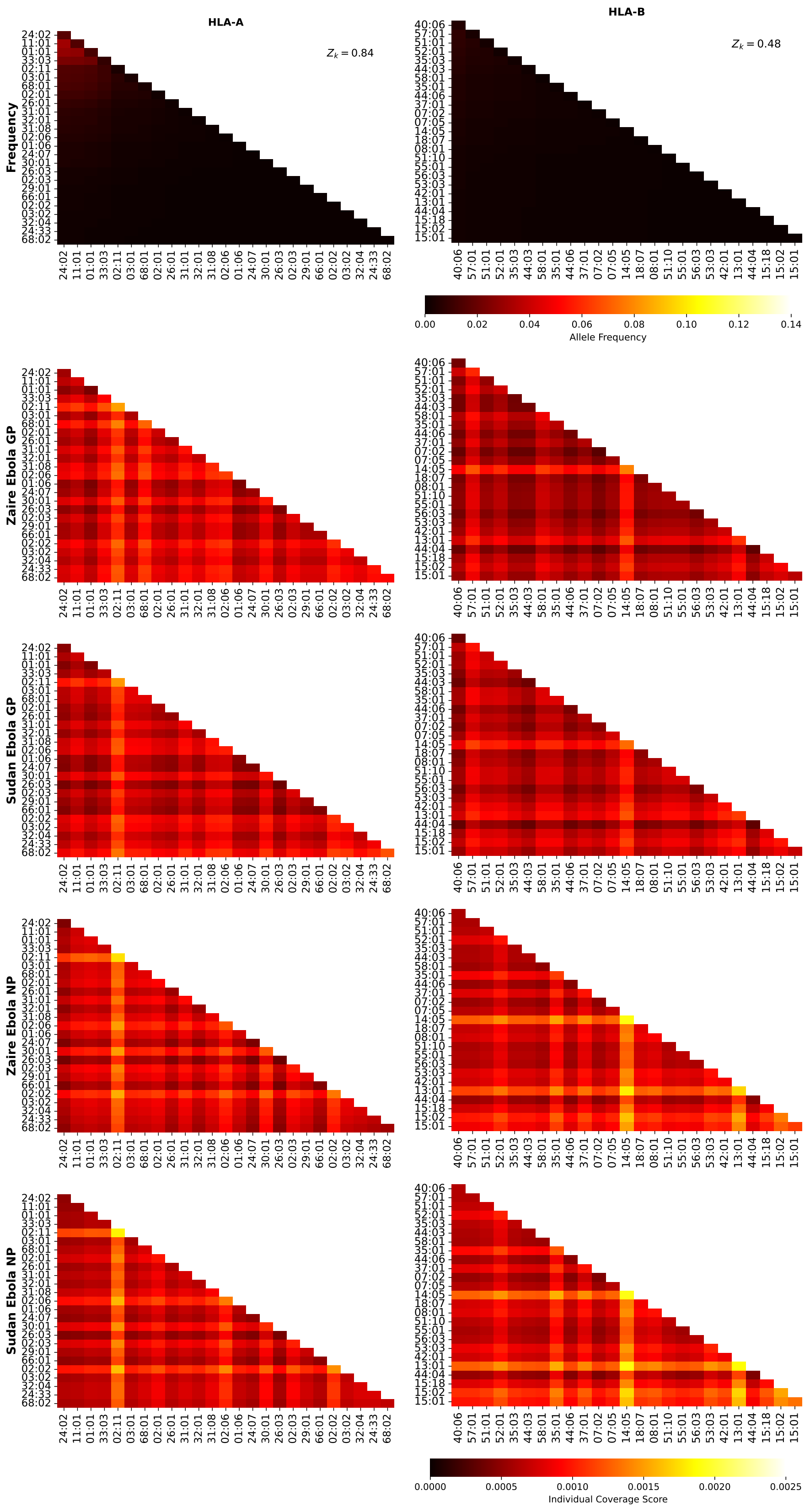

Figure S72. Frequencies and Ebola coverage scores for individuals in South Asia. The 1st row corresponds to allele frequencies, the 2nd to GP1 Zaire, the 3rd to GP1 Sudan, the 4th to NP Zaire, and the 5th to NP Sudan. The 1st column is associated with HLA-A alleles, the 2nd to HLA-B, and the 3rd to HLA-C. The sum of the individual frequencies for each allele type is indicated on the panels in the 1st row.

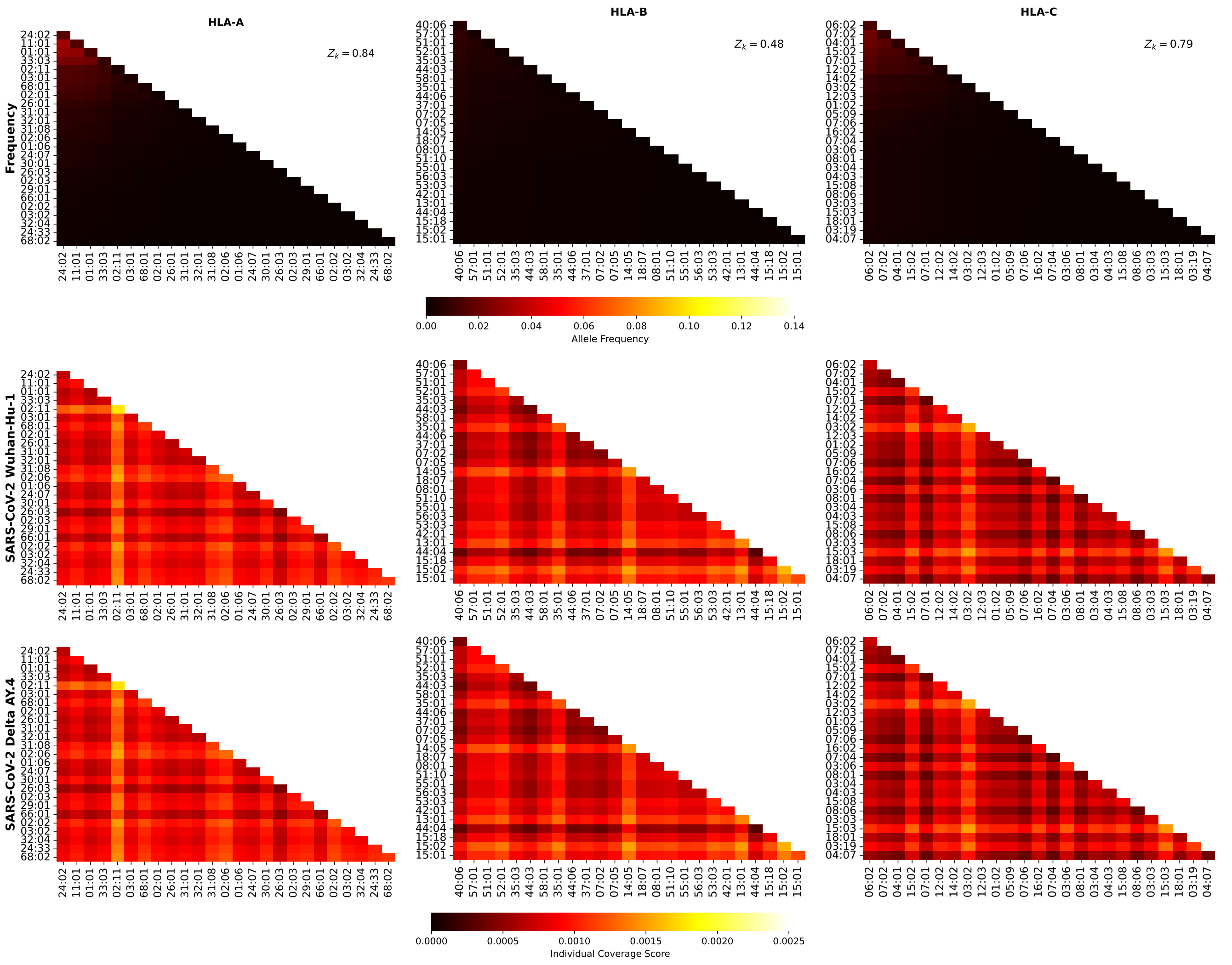

Figure S73. Frequencies and SARS-CoV-2 (Wuhan-Hu-1 and Delta AY.4 variants) coverage scores for individuals in South Asia. The 1st row corresponds to allele frequencies, the 2nd to Wuhan-Hu-1, and the 3rd to Delta AY.4. The 1st column is associated with HLA-A alleles, the 2nd to HLA-B, and the 3rd to HLA-C. The sum of the individual frequencies for each allele type is indicated on the panels in the 1st row.

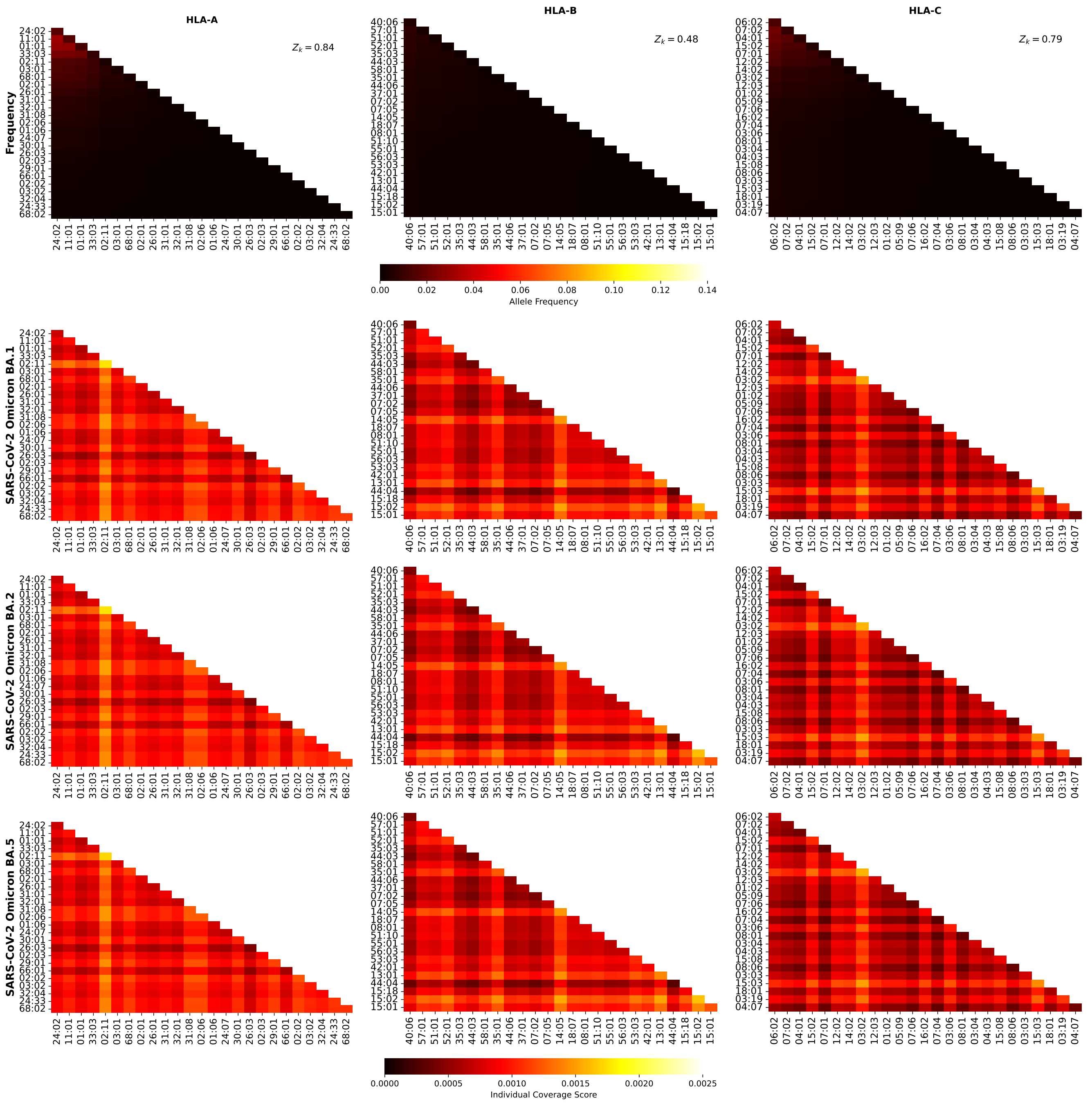

Figure S74. Frequencies and SARS-CoV-2 (Omicron variants) coverage scores for individuals in South Asia. The 1st row corresponds to allele frequencies, the 2nd to BA.1, and the 3rd to BA.2, and the 4th to BA.5. The 1st column is associated with HLA-A alleles, the 2nd to HLA-B, and the 3rd to HLA-C. The sum of the individual frequencies for each allele type is indicated on the panels in the 1st row.

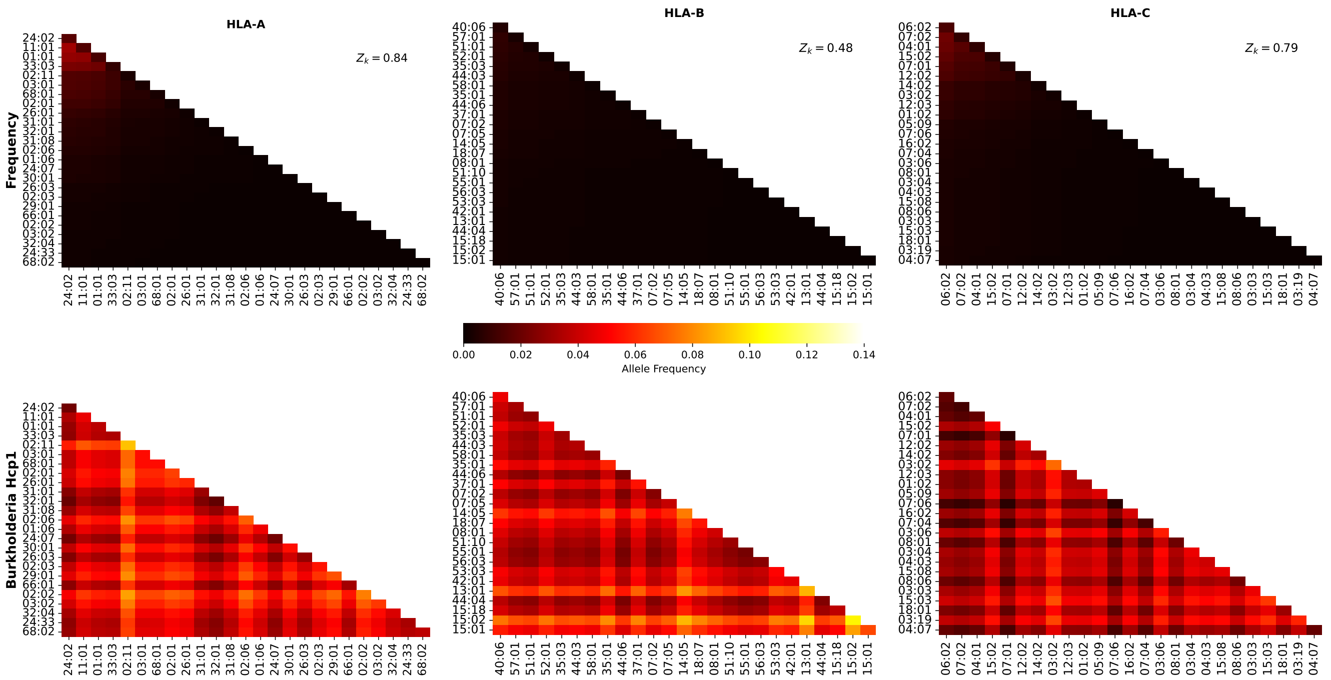

Figure S75. Frequencies and Burkholderia coverage scores for individuals in South Asia. The 1st row corresponds to allele frequencies and the 2nd row to Burkholderia coverage score. The 1st column is associated with HLA-A alleles, the 2nd to HLA-B, and the 3rd to HLA-C. The sum of the individual frequencies for each allele type is indicated on the panels in the 1st row.

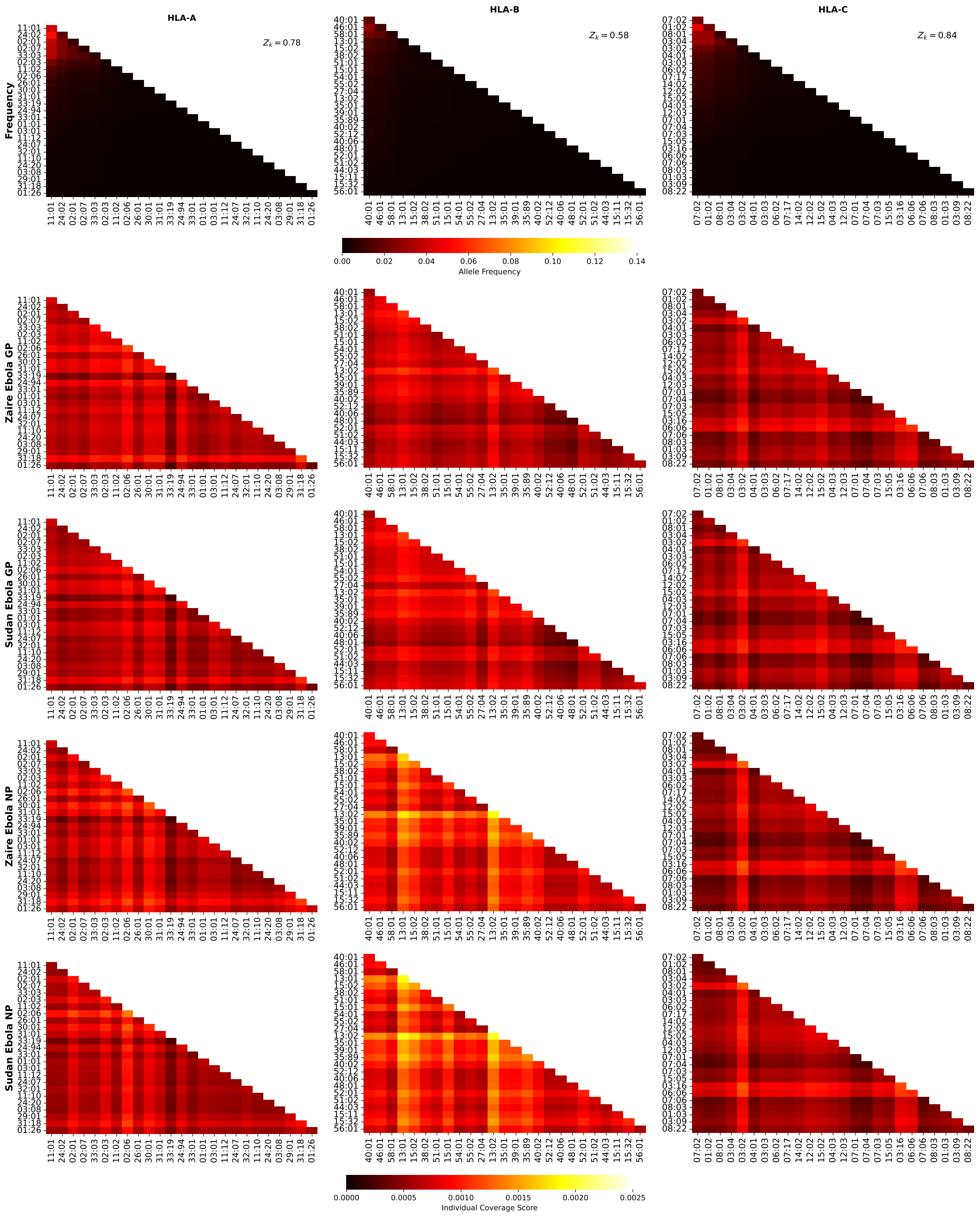

Figure S76. Frequencies and Ebola coverage scores for individuals in Southeast Asia. The 1st row corresponds to allele frequencies, the 2nd to GP1 Zaire, the 3rd to GP1 Sudan, the 4th to NP Zaire, and the 5th to NP Sudan. The 1st column is associated with HLA-A alleles, the 2nd to HLA-B, and the 3rd to HLA-C. The sum of the individual frequencies for each allele type is indicated on the panels in the 1st row.

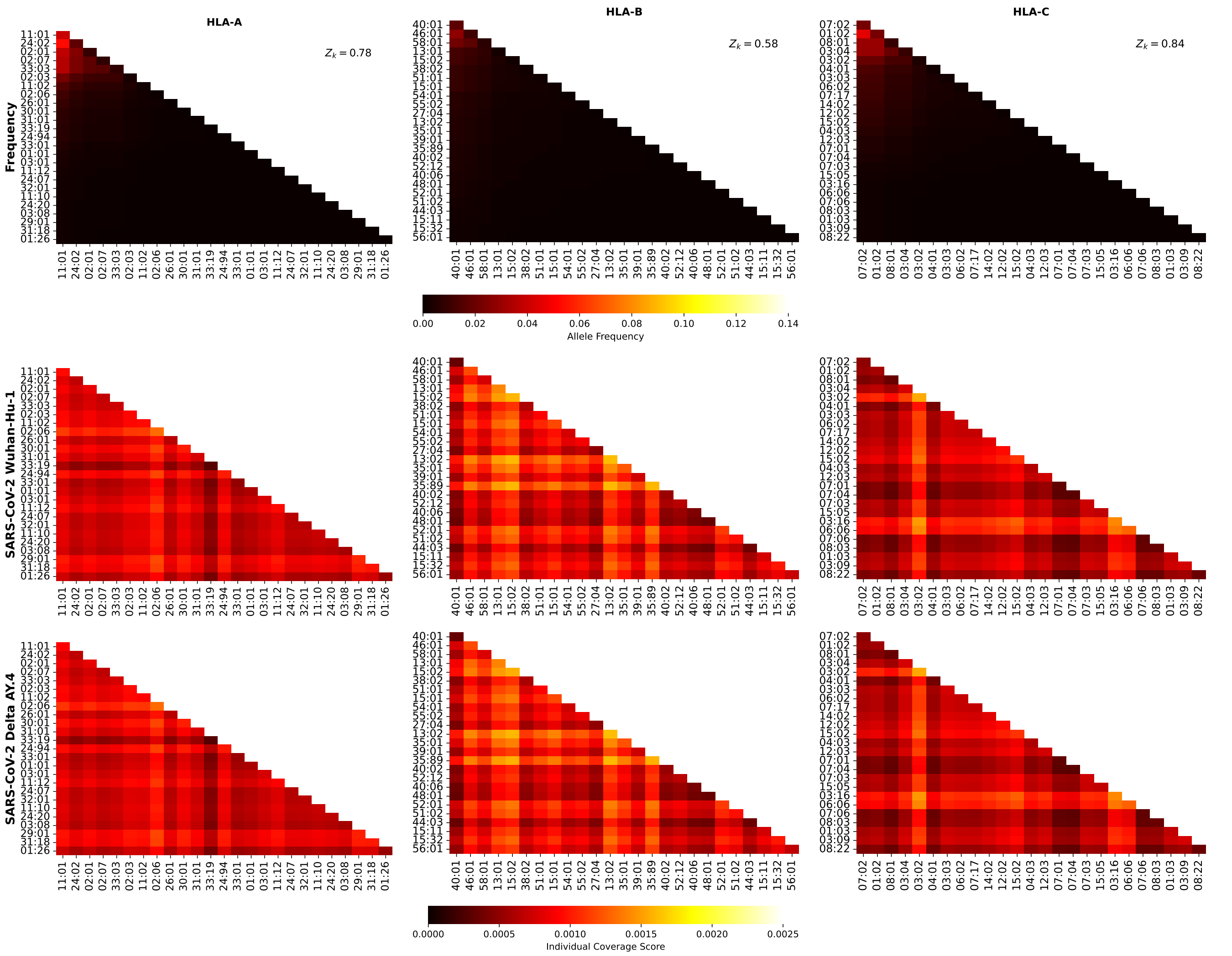

Figure S77. Frequencies and SARS-CoV-2 (Wuhan-Hu-1 and Delta AY.4 variants) coverage scores for individuals in Southeast Asia. The 1st row corresponds to allele frequencies, the 2nd to Wuhan-Hu-1, and the 3rd to Delta AY.4. The 1st column is associated with HLA-A alleles, the 2nd to HLA-B, and the 3rd to HLA-C. The sum of the individual frequencies for each allele type is indicated on the panels in the 1st row.

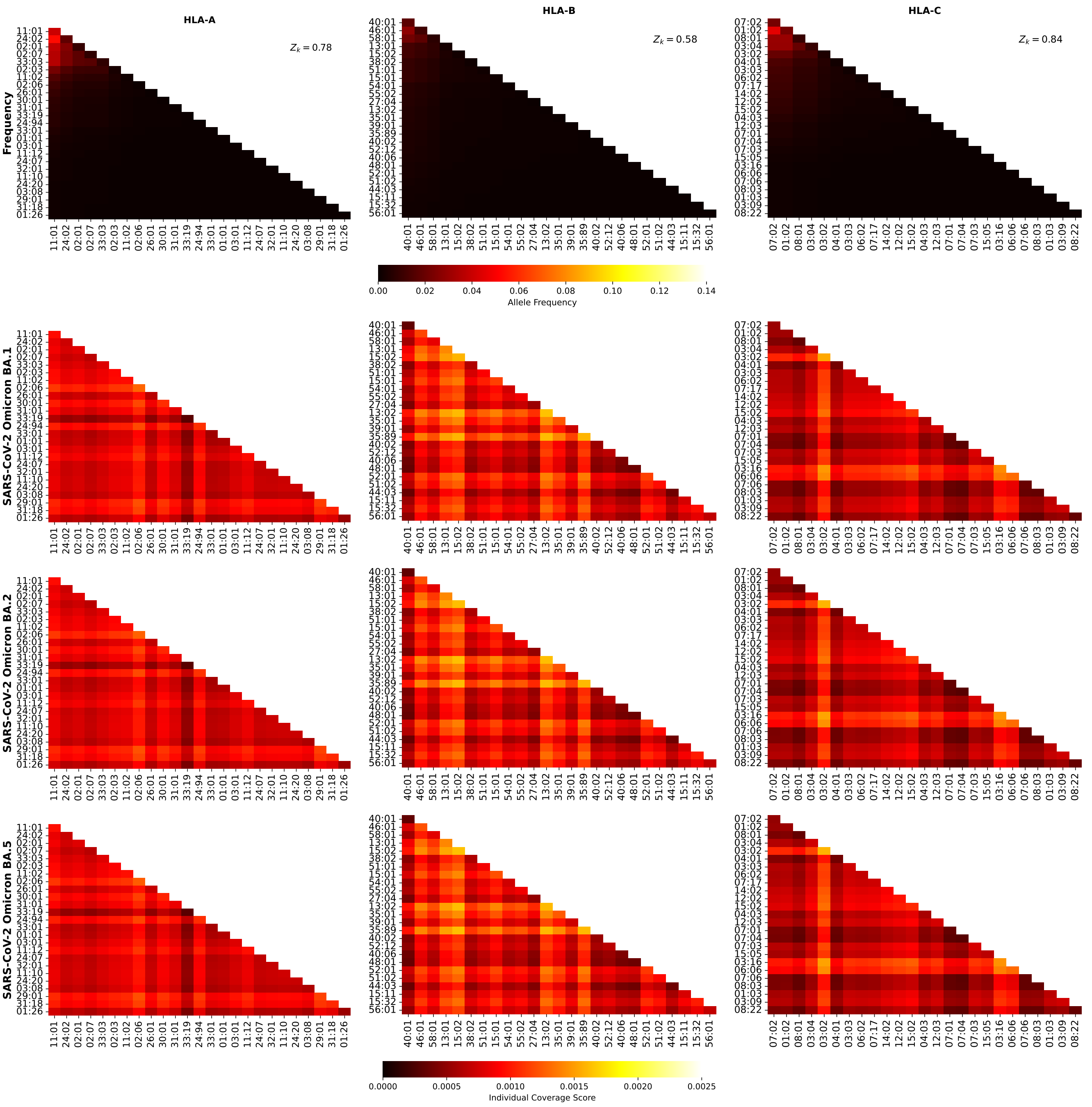

Figure S78. Frequencies and SARS-CoV-2 (Omicron variants) coverage scores for individuals in Southeast Asia. The 1st row corresponds to allele frequencies, the 2nd to BA.1, and the 3rd to BA.2, and the 4th to BA.5. The 1st column is associated with HLA-A alleles, the 2nd to HLA-B, and the 3rd to HLA-C. The sum of the individual frequencies for each allele type is indicated on the panels in the 1st row.

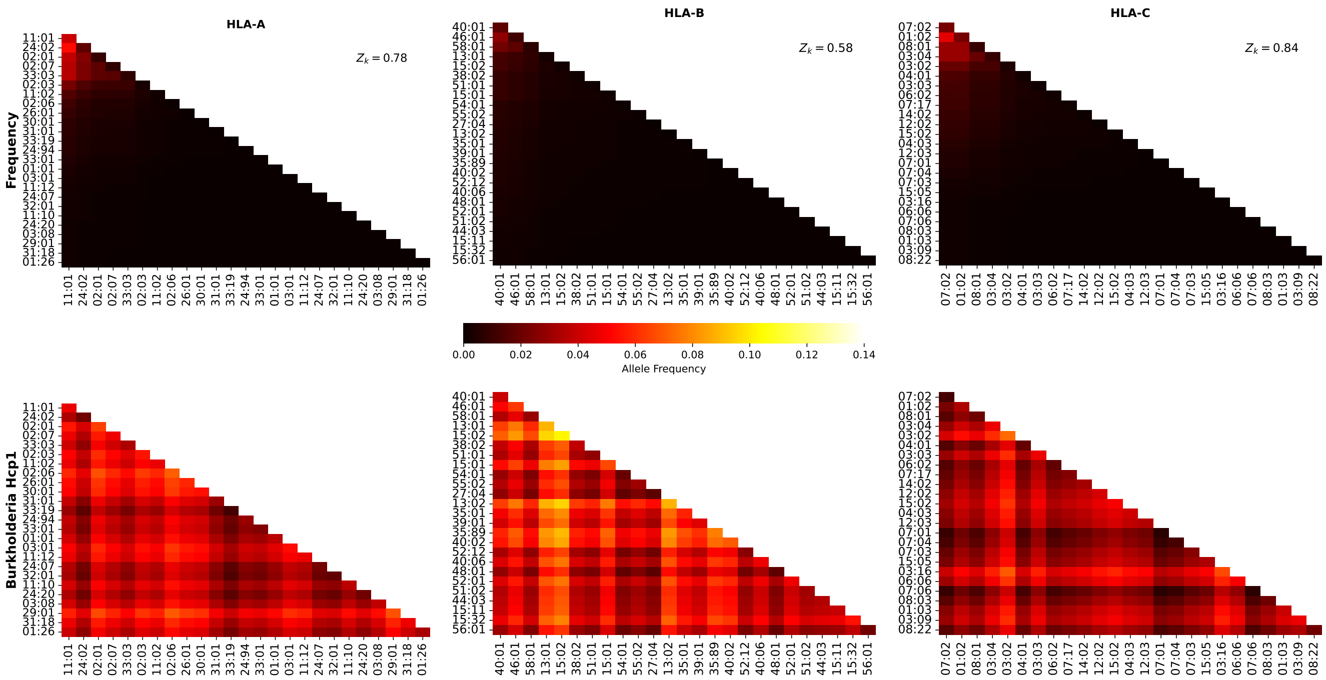

Figure S79. Frequencies and Burkholderia coverage scores for individuals in Southeast Asia. The 1st row corresponds to allele frequencies and the 2nd row to Burkholderia coverage score. The 1st column is associated with HLA-A alleles, the 2nd to HLA-B, and the 3rd to HLA-C. The sum of the individual frequencies for each allele type is indicated on the panels in the 1st row.

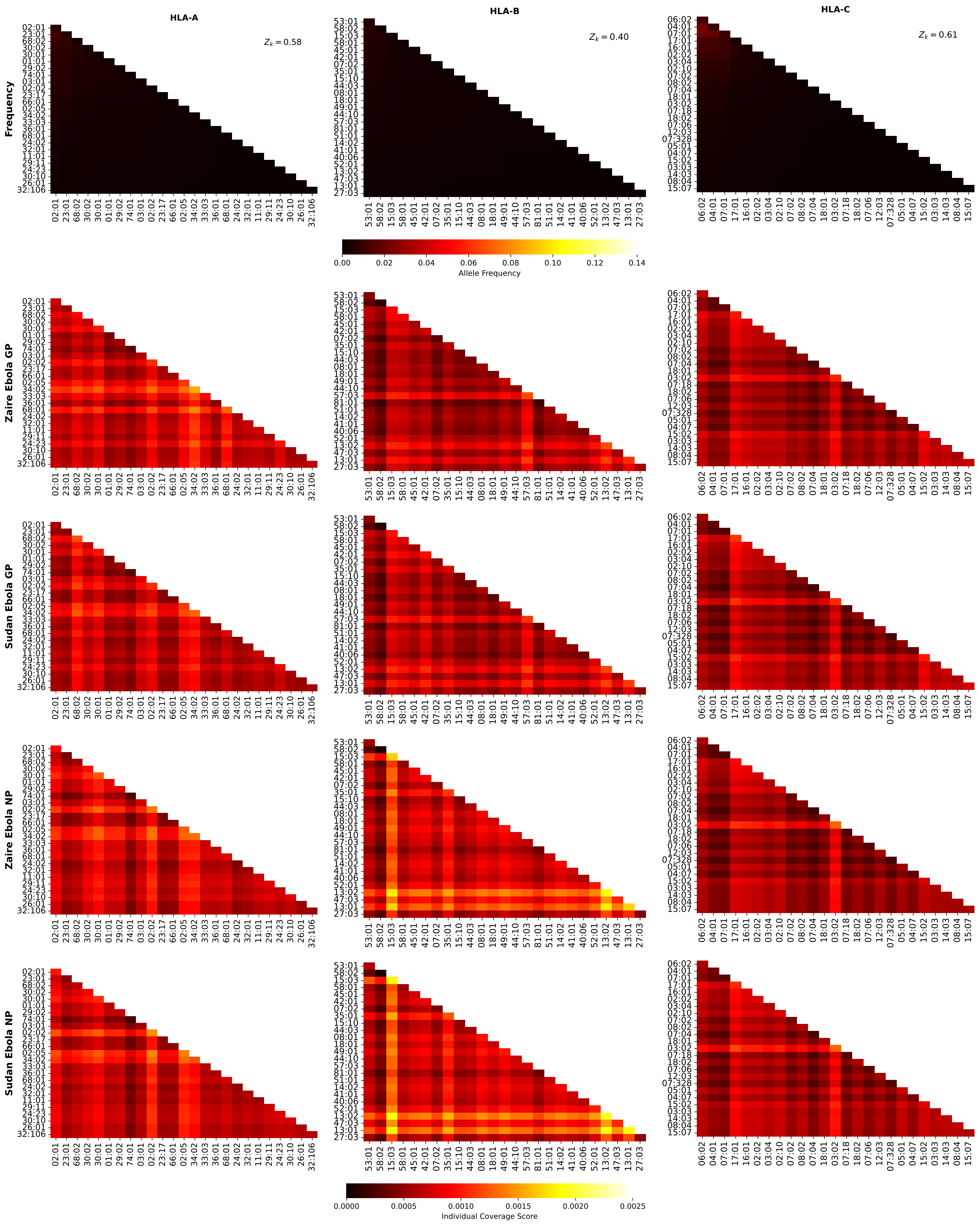

Figure S80. Frequencies and Ebola coverage scores for individuals in Sub-Saharan Africa. The 1st row corresponds to allele frequencies, the 2nd to GP1 Zaire, the 3rd to GP1 Sudan, the 4th to NP Zaire, and the 5th to NP Sudan. The 1st column is associated with HLA-A alleles, the 2nd to HLA-B, and the 3rd to HLA-C. The sum of the individual frequencies for each allele type is indicated on the panels in the 1st row.

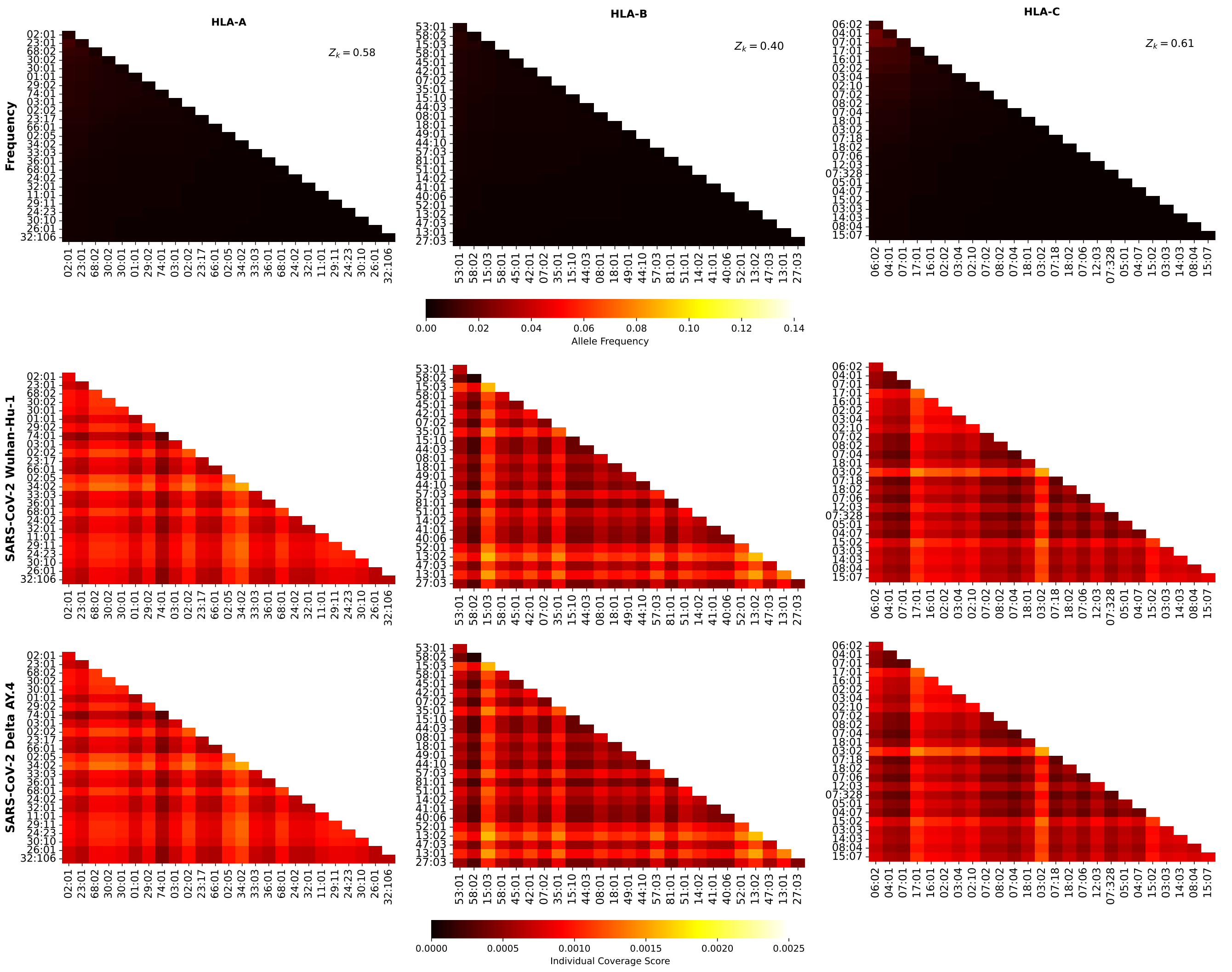

Figure S81. Frequencies and SARS-CoV-2 (Wuhan-Hu-1 and Delta AY.4 variants) coverage scores for individuals in Sub-Saharan Africa. The 1st row corresponds to allele frequencies, the 2nd to Wuhan-Hu-1, and the 3rd to Delta AY.4. The 1st column is associated with HLA-A alleles, the 2nd to HLA-B, and the 3rd to HLA-C. The sum of the individual frequencies for each allele type is indicated on the panels in the 1st row.

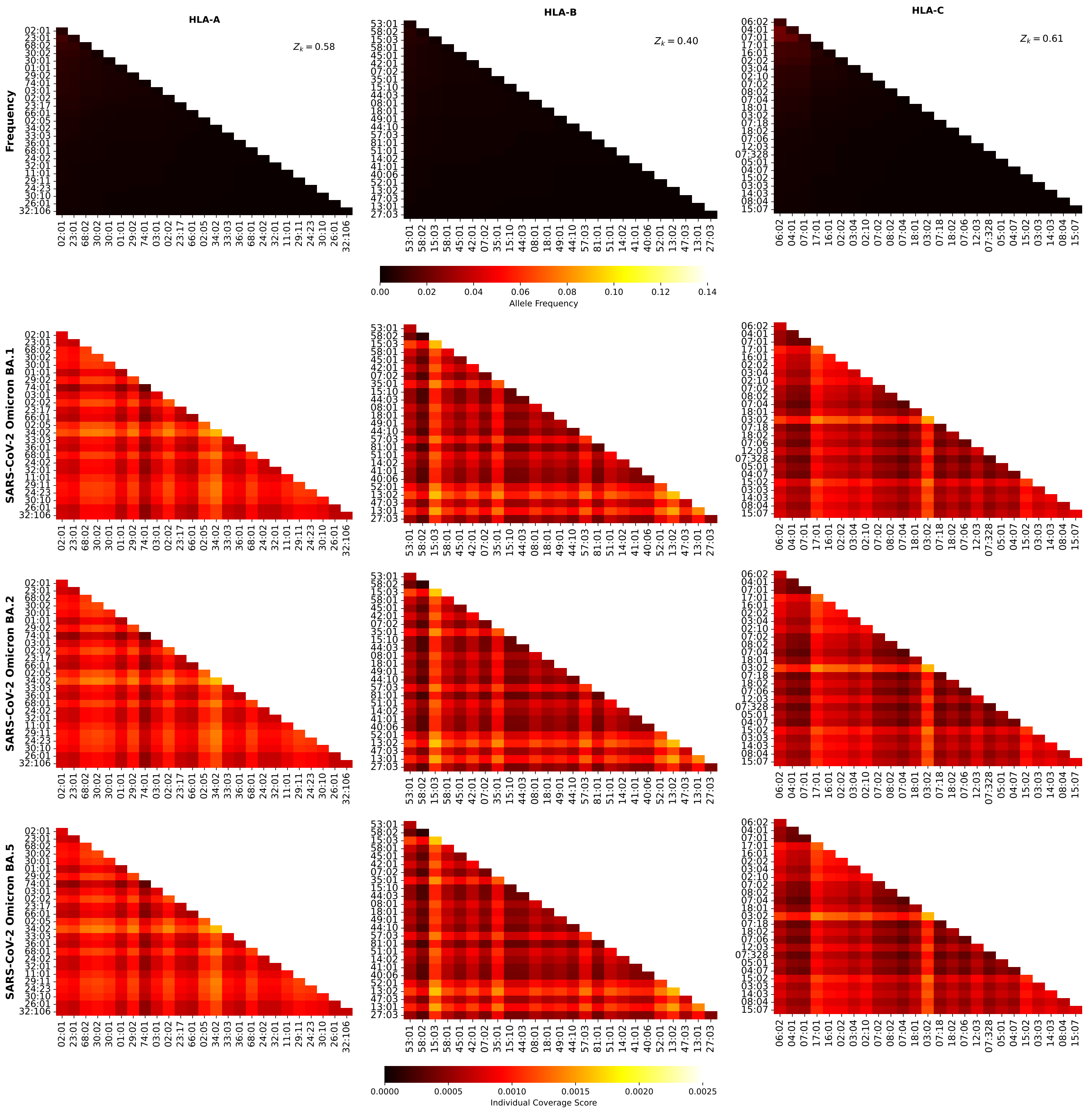

Figure S82. Frequencies and SARS-CoV-2 (Omicron variants) coverage scores for individuals in Sub-Saharan Africa. The 1st row corresponds to allele frequencies, the 2nd to BA.1, and the 3rd to BA.2, and the 4th to BA.5. The 1st column is associated with HLA-A alleles, the 2nd to HLA-B, and the 3rd to HLA-C. The sum of the individual frequencies for each allele type is indicated on the panels in the 1st row.

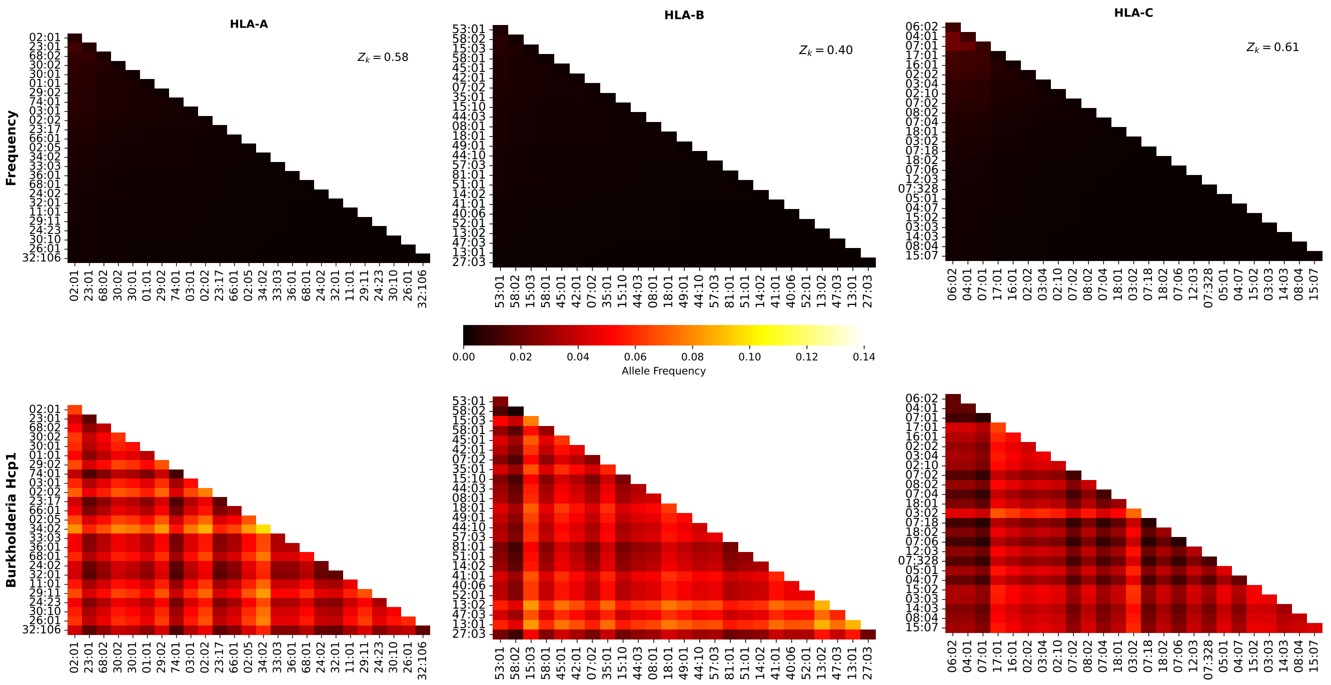

Figure S83. Frequencies and Burkholderia coverage scores for individuals in Sub-Saharan Africa. The 1st row corresponds to allele frequencies and the 2nd row to Burkholderia coverage score. The 1st column is associated with HLA-A alleles, the 2nd to HLA-B, and the 3rd to HLA-C. The sum of the individual frequencies for each allele type is indicated on the panels in the 1st row.

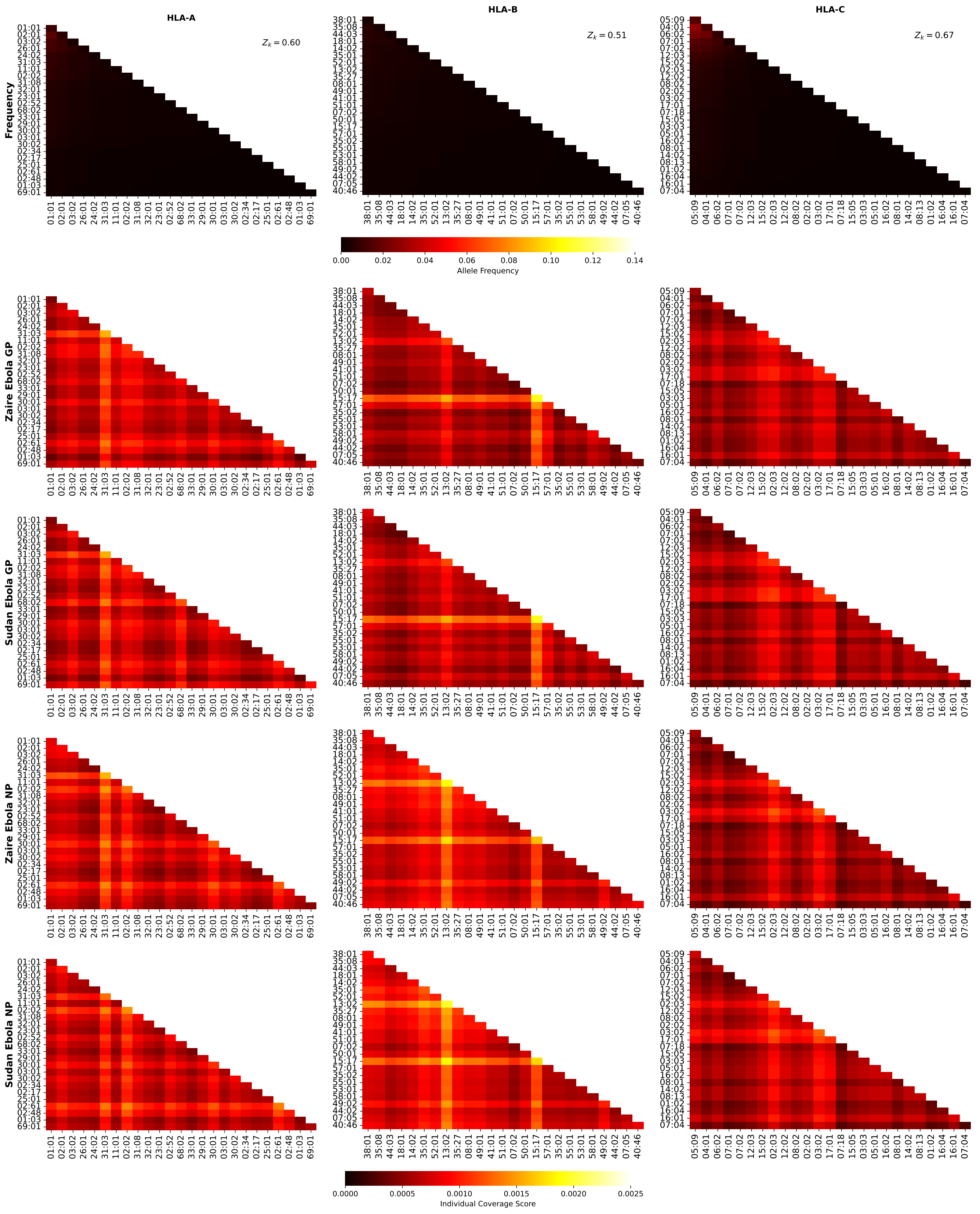

Figure S84. Frequencies and Ebola coverage scores for individuals in Western Asia. The 1st row corresponds to allele frequencies, the 2nd to GP1 Zaire, the 3rd to GP1 Sudan, the 4th to NP Zaire, and the 5th to NP Sudan. The 1st column is associated with HLA-A alleles, the 2nd to HLA-B, and the 3rd to HLA-C. The sum of the individual frequencies for each allele type is indicated on the panels in the 1st row.

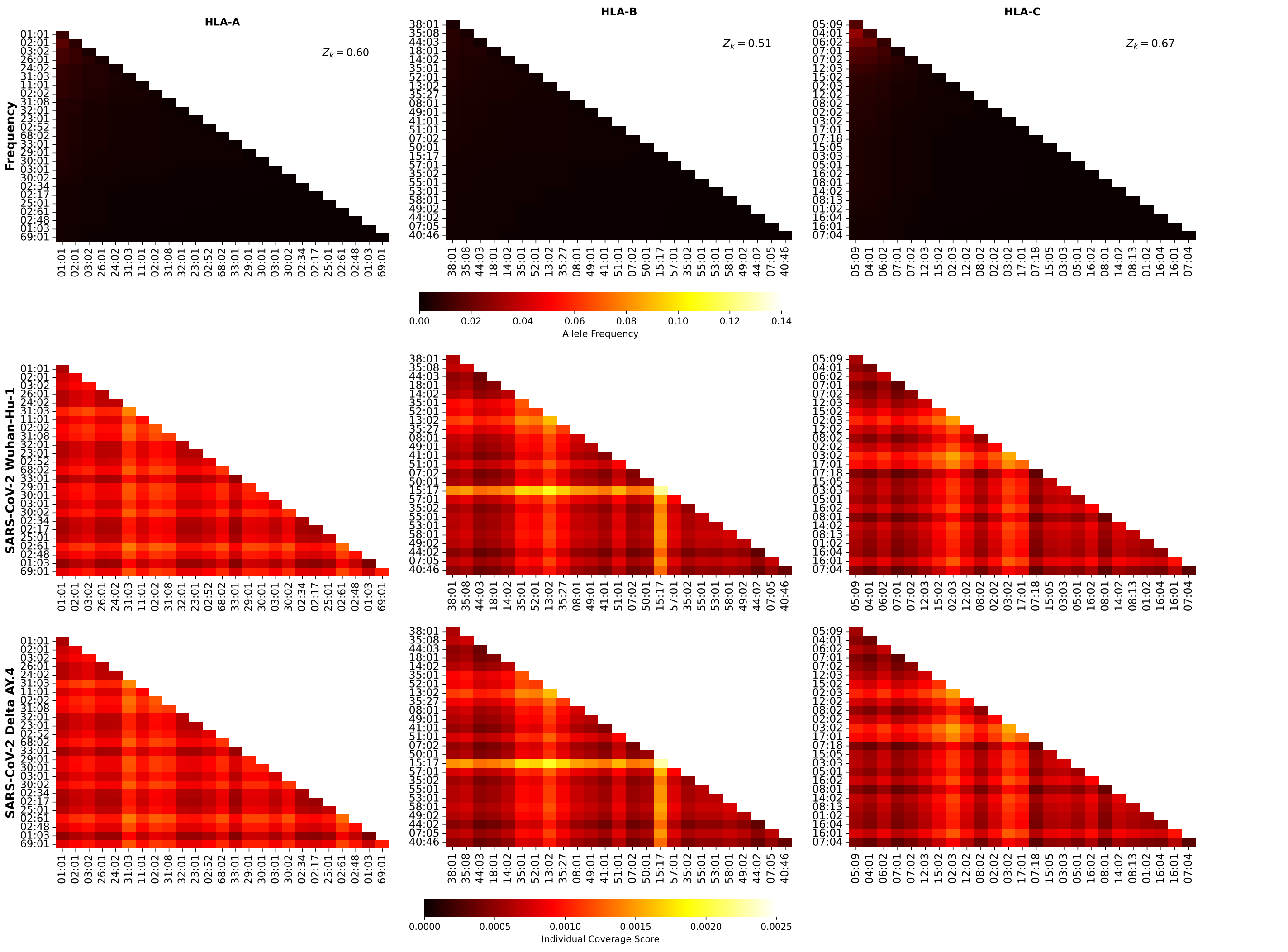

Figure S85. Frequencies and SARS-CoV-2 (Wuhan-Hu-1 and Delta AY.4 variants) coverage scores for individuals in Western Asia. The 1st row corresponds to allele frequencies, the 2nd to Wuhan-Hu-1, and the 3rd to Delta AY.4. The 1st column is associated with HLA-A alleles, the 2nd to HLA-B, and the 3rd to HLA-C. The sum of the individual frequencies for each allele type is indicated on the panels in the 1st row.

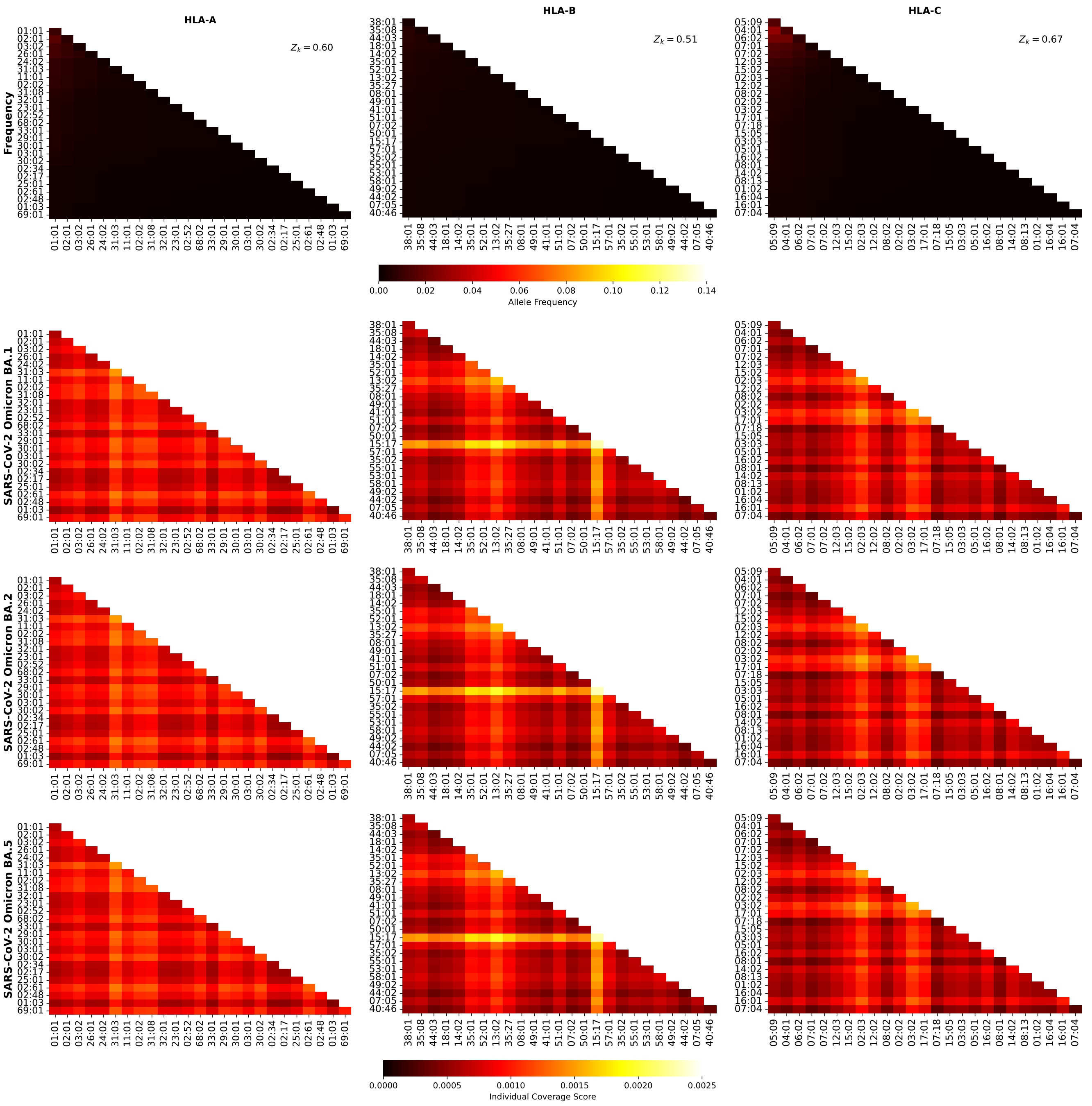

Figure S86. Frequencies and SARS-CoV-2 (Omicron variants) coverage scores for individuals in Western Asia. The 1st row corresponds to allele frequencies, the 2nd to BA.1, and the 3rd to BA.2, and the 4th to BA.5. The 1st column is associated with HLA-A alleles, the 2nd to HLA-B, and the 3rd to HLA-C. The sum of the individual frequencies for each allele type is indicated on the panels in the 1st row.

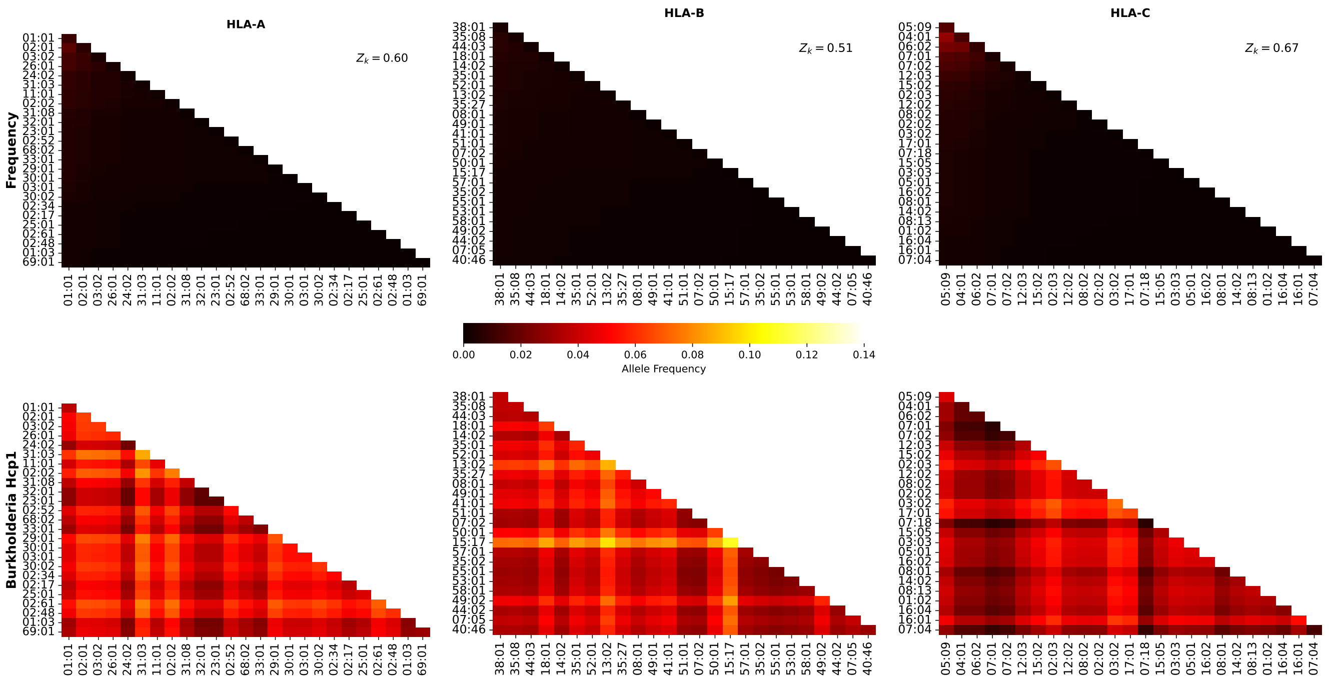

Figure S87. Frequencies and Burkholderia coverage scores for individuals in Western Asia. The 1st row corresponds to allele frequencies and the 2nd row to Burkholderia coverage score. The 1st column is associated with HLA-A alleles, the 2nd to HLA-B, and the 3rd to HLA-C. The sum of the individual frequencies for each allele type is indicated on the panels in the 1st row.

REFERENCES
